# A molecular array for 10-second diagnosis of common spinal tumor types with picosecond infrared laser mass spectrometry

**DOI:** 10.1093/neuonc/noaf047

**Published:** 2025-02-21

**Authors:** Alexa Fiorante, Michael Woolman, David G Munoz, Taira Kiyota, Lan Anna Ye, Yasmine Farahmand, Darah Vlaminck, Francis O Talbot, Sunit Das, Sorcha Kellett, Christine Giuffrida, Gelareh Zadeh, Howard J Ginsberg, Ahmed Aman, Arash Zarrine-Afsar

**Affiliations:** Department of Medical Biophysics, University of Toronto, Toronto, Ontario, Canada; Princess Margaret Cancer Centre, University Health Network, Toronto, Ontario, Canada; Department of Medical Biophysics, University of Toronto, Toronto, Ontario, Canada; Princess Margaret Cancer Centre, University Health Network, Toronto, Ontario, Canada; Keenan Research Center for Biomedical Science & The Li Ka Shing Knowledge Institute, St. Michael’s Hospital, Toronto, Ontario, Canada; Department of Laboratory Medicine and Pathobiology, University of Toronto, Toronto, Ontario, Canada; Drug Discovery Group, Ontario Institute for Cancer Research (OICR), Toronto, Ontario, Canada; Princess Margaret Cancer Centre, University Health Network, Toronto, Ontario, Canada; Princess Margaret Cancer Centre, University Health Network, Toronto, Ontario, Canada; Department of Medical Biophysics, University of Toronto, Toronto, Ontario, Canada; Princess Margaret Cancer Centre, University Health Network, Toronto, Ontario, Canada; Princess Margaret Cancer Centre, University Health Network, Toronto, Ontario, Canada; Keenan Research Center for Biomedical Science & The Li Ka Shing Knowledge Institute, St. Michael’s Hospital, Toronto, Ontario, Canada; Department of Surgery, University of Toronto, Toronto, Ontario, Canada; Keenan Research Center for Biomedical Science & The Li Ka Shing Knowledge Institute, St. Michael’s Hospital, Toronto, Ontario, Canada; Keenan Research Center for Biomedical Science & The Li Ka Shing Knowledge Institute, St. Michael’s Hospital, Toronto, Ontario, Canada; Department of Surgery, University of Toronto, Toronto, Ontario, Canada; Princess Margaret Cancer Centre, University Health Network, Toronto, Ontario, Canada; Keenan Research Center for Biomedical Science & The Li Ka Shing Knowledge Institute, St. Michael’s Hospital, Toronto, Ontario, Canada; Department of Surgery, University of Toronto, Toronto, Ontario, Canada; Department of Laboratory Medicine and Pathobiology, University of Toronto, Toronto, Ontario, Canada; Leslie Dan, Faculty of Pharmacy, University of Toronto, Toronto, Ontario, Canada; Drug Discovery Group, Ontario Institute for Cancer Research (OICR), Toronto, Ontario, Canada; Keenan Research Center for Biomedical Science & The Li Ka Shing Knowledge Institute, St. Michael’s Hospital, Toronto, Ontario, Canada; Department of Surgery, University of Toronto, Toronto, Ontario, Canada; Department of Medical Biophysics, University of Toronto, Toronto, Ontario, Canada; Princess Margaret Cancer Centre, University Health Network, Toronto, Ontario, Canada

**Keywords:** diagnostic biomarkers, lipidomics, mass spectrometry, non-subjective diagnosis, picosecond infrared laser mass spectrometry, rapid diagnosis, spinal tumors

## Abstract

**Background:**

Improving the surgical outcomes for commonly occurring spinal neoplasms of extradural and intradural extramedullary origins requires precise intraoperative diagnosis provided by highly trained neuropathologists.

**Methods:**

Through a retrospective study of *n* = 319 patient specimens, verified where appropriate by learning curve analysis to be sufficient for statistically significant observations, we aimed to assess the utility of 10-second picosecond infrared laser mass spectrometry (MS; PIRL-MS) for non-subjective diagnosis of major spinal tumor types of metastatic carcinoma, schwannoma, and meningiomas.

**Results:**

The sensitivity and specificity values of spinal tumor-type diagnosis (based on *n* = 182 independent specimens) were (93% ± 1)% and (97% ± 2)%, respectively. This classification utilizes *n* = 41 cellular lipids including phosphatidylcholines, sphingomyelins, phosphatidylethanolamines, and ceramides whose identities were established using high-resolution tandem MS. Furthermore, the accuracy of diagnosis of a model that contained *n* = 97 meningioma and *n* = 106 schwannoma was not drastically influenced by the presence of *n* = 54 additional intradural extramedullary spinal neoplasms of myxopapillary ependymoma, neurofibroma, paraganglioma, and solitary fibrous tumor types in the differential diagnosis, confirming the generalizability and robustness of the identified molecular array in rendering correct classification even in the presence of data not seen previously by the model.

**Conclusions:**

The identified lipids form a “molecular array” for robust diagnosis of meningioma and schwannoma tumors by non-pathologists in a manner similar to genomic, transcriptomic, or methylomic arrays used to diagnose brain cancer types, albeit on a much faster timescale of seconds as opposed to hours.

**Key Points**

1. A biomarker array of 41 cellular lipids allows a 10-second diagnosis of spinal tumors.
2. Lipidomics allows non-subjective, high sensitivity, and specificity diagnosis of spinal tumors.

**Importance of the Study**

Currently, intrasurgical diagnosis of spinal tumor types to guide the extent of surgical resection relies on an intraoperative consult with a highly trained neuropathologist on call. A molecular array comprised of 41 cellular lipids is shown to be sufficient for automated, non-subjective diagnosis of these tumor types using 10-second laser mass spectrometry with sensitivity and specificity of >93%, thus extending the best surgical care to settings understaffed in neuropathology.

Spinal tumors account for 20% of all central nervous system tumors^1^ with incidence rates of up to 70 per 100 000^2–5^ and are classified into 2 major classes: extradural neoplasms (dominated by metastatic cancers) and intradural extramedullary tumors.^6,7^ The third class of intradural intramedullary tumors only accounts for 5% of all spinal neoplasms.^6^ Of these classes, intradural extramedullary tumors (~40% of all spinal cancers^6,8^) pose a high morbidity risk due to the neurologic deficits they cause.^9^ Within this class, meningiomas (the most common central nervous system tumors^10^) and schwannomas are the 2 most frequently occurring types.^9^ Meningiomas, although largely benign, possess a high propensity to recur after surgical removal and evidence exists that they must be resected completely with dural excisions to improve the surgical outcomes.^11–16^ Comparatively, in schwannoma excisions, it is warranted that some element of nerve structure bearing the tumor be sacrificed to improve the surgical outcomes.^17,18^ Schwannoma tumors arise from Schwann cells, a component of the nerve structure itself that becomes a “capsule” for the tumor,^15,19,20^ requiring this different surgical method. As such, the neurosurgeon requires an accurate diagnosis of commonly occurring intradural extramedullary tumor types to plan the most suitable surgical approach, justifying the resection plan and its associated risks.

Since preoperative biopsies are uncommon in neurosurgery, the diagnosis of meningioma or schwannoma to allow a personalized resection extent commensurate with evidence-based improvement in outcomes^13^ relies on intraoperative pathology consultations, complementing preoperative imaging and intraoperative observations. Due to time constraints (patient awaiting pathology results under anesthesia), intraoperative pathology consults use “frozen section” analysis where thin slices of the surgical specimen are rapidly prepared and quickly stained to reveal morphometric differences between different tumor types towards a diagnosis. While the diagnosis of meningiomas is often relatively straightforward for a highly experienced neuropathologist,^21,22^ not all surgeries proceed with intraoperative pathology guidance due to human resource limitations in pathology,^23^ a bottleneck for many healthcare centres^24^ that is further compounded by poor(er) performance of general pathologists when tasked with neurosurgery consults.^25^ Additionally, the diagnosis rate for meningioma remains suboptimal,^26^ especially with much of the 27% reported discordance stemming from confusion between certain subtypes (eg, fibroblastic meningiomas that could be mimicking schwannomas during frozen section analysis^27,28^). These limitations have created an impetus for the development of a non-subjective method of intradural extramedullary tumor diagnosis (with a potential outreach domain of several hundred thousand patients worldwide based on current incidence rates) such that accurate differentiation between meningioma and schwannoma tumors becomes possible, uninfluenced by human factors stated above.^25^ Driven by this need, preoperative magnetic resonance imaging augmented with artificial intelligence analysis methods has been proposed to address the aforementioned limitations and reduce the subjectivity of diagnosis for meningioma tumors^29,30^ and their differentiation from schwannomas. In a recent study, through the use of select changes in MR signals in combination with deep learning methods, encouraging accuracies in the 80% range (over ~100 patient cohort study) for the differentiation of meningioma from schwannomas have been reported.^30^ However, a molecular method for rapid identification of frequently occurring spinal tumors using the fundamental observable changes in the tissue chemistry correlated to each tumor type is currently lacking.

To this end, we are developing a method of biological tissue type characterization that uses a fingerprint of tissue lipids both present in cellular membranes (to recapitulate morphometric differences in cellular shapes currently used in microscopy) and altered in tumorigenesis^31^ (to recapitulate certain molecular pathology indications) with only ~10 seconds of data collection and analysis time, utilizing defined, discrete and measurable cellular molecules as its diagnostic “features” to differentiate between various spinal tumor pathologies. The goal will be to provide the neurosurgeon with an accurate diagnosis of meningioma such that personalized resections with improved outcomes^12^ could be offered in the initial surgery. The method uses a picosecond infrared laser to desorb tissue lipids into the gas phase. Then, these gas-phase lipids will be subjected to mass spectrometry (MS) and data analysis to create a molecular fingerprint profile for tissue-classifying lipids. Subsequently, the lipid fingerprint profile of a query surgical specimen can be compared to a previously established (and validated) lipid fingerprint library for all relevant tumor types in differential diagnosis to find a match. This step utilizes multivariate statistical analyses performed in a fraction of a second. Our approach is entitled Picosecond Infrared Laser MS (PIRL-MS) and has been utilized to determine, with high (> 95%) sensitivity and specificity, select molecular subgroups as well as broader morphologically distinct classes of pediatric brain cancers,^32^ medulloblastoma subgroups,^33,34^ skin cancer types,^35^ and BRAF-V600E mutational status^36^ from correlated lipid markers, all upon only ~10 seconds of data collection and analysis time. The most time-consuming step in our workflow is the creation of the lipid fingerprint profile library for relevant tumor types in differential diagnosis. Upon the availability of this library, users have access to an appropriate PIRL-MS molecular [diagnosis] model for tumor types in differential diagnosis. PIRL-MS is an “ambient” MS analysis tool^37^ that like its counterparts that use plume of electrosurgery,^38^ lasers^39^ or gentle solvent extraction^40^ is capable of reliably obtaining tissue-specific (and thus tissue type-classifying) molecular profiles under ambient atmospheric conditions without sample preparation. Ambient MS has gained traction as an emerging field suited for rapid cancer diagnosis.^37^ While PIRL-MS is competent in both rapid classification of various morphologically distinct classes of tumors^32,35^ as well as those only molecularly distinct,^33,41^ this manuscript largely utilizes the non-subjective and rapid diagnosis features of the PIRL-MS method to enable differential diagnosis of meningioma and schwannoma tumors where a clear statement of need and clinical utility could be articulated, as laid out above.

## Materials and Methods

### Tissues and Samples

#### Ethics statement.—

Flash frozen banked patient specimens were sourced from local biobanks as approved per the institutional review board and subsequent authorization by the research ethics board of the University Health Network. The authorization for specimen use for research purposes was obtained through *written informed consent* secured at the biobanking stage, and then evaluated by the ethics board for use in the context of the specific aims of this publication (authorization UHN REB 21-5929). This study used *n* = 319 specimens in total. Here, metastatic carcinomas (*n* = 62), meningioma (*n* = 97), schwannoma (*n* = 106), myxopapillary ependymoma (*n* = 18), neurofibroma (*n* = 18), paraganglioma (*n* = 9), solitary fibrous tumors also known as hemangiopericytoma (*n* = 9) were subjected to PIRL-MS analysis. See SI section “Additional Information: Experimental methods” for how ground truth pathology for these specimens were established.

### PIRL-MS Data Collection and Analysis

The molecular profiles (100–1000 Da) were collected on a Xevo G2-XS quadrupole Time-of-Flight mass spectrometer (Waters) in the negative ion mode. Desorption was performed by a PIRL system (Light-Matter Interaction) in a hand-held mode through a 0.4 mm sapphire fiber at 1 kHz (300–400 mW of average power). The actual average signal duration for sampling was 13 ± 2 seconds, and the plume of the laser-ablated material was transported to the qTOF instrument using ~ 2 m Tygon tubing interfaced with the metallic extension of the ion block (heated to 150 °C). This used an ion block extension provided with DESI-MS source (Waters), utilized for convenience. The act of sampling resulted in the collection of multiple PIRL-MS spectra from the surface of each specimen. Each mass spectrum forms a single sampling event comprised of *m/z* values (100–1000 Da) averaged from an ~10-second data collection. Additional details regarding PIRL-MS data analysis and identification of discriminating ions with liquid chromatography are included in the SI section “Additional Information: Experimental methods.”

## Results

### Creation of a Molecular Model for 10-second Classification of Common Spinal Tumor Types With PIRL-MS

**Figure 1.**
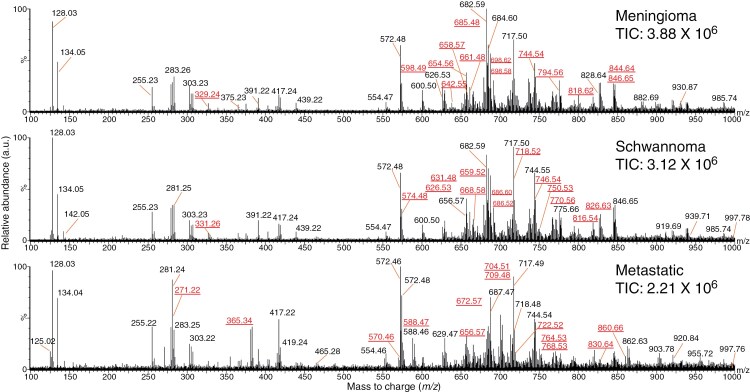
Representative 10-second PIRL-MS spectra of meningioma, schwannoma, and metastatic cancers. In this figure, we are providing the representative PIRL-MS spectra of the major types of spinal tumors alongside the total ion count (TIC) values for these spectra. Here, we have highlighted the abundant *m/z* values in black font. As can be seen here, PIRL-MS profiling across the mass range of 100–1000 Da (Daltons) reveals distinct molecular profiles for these 3 tumor types. The *n* = 41 ions important for discrimination (Table 2) were visible in these representative spectra (highlighted in red font / underlined). A qualitative comparison of Figure 1 and Table 2 results with desorption electrospray ionization mass spectrometry (DESI-MS) spectra of meningiomas^43^suggests complementary molecular information between PIRL-MS and DESI-MS as previously reported using murine models of human medulloblastoma brain cancers.^44^

**Figure 2.**
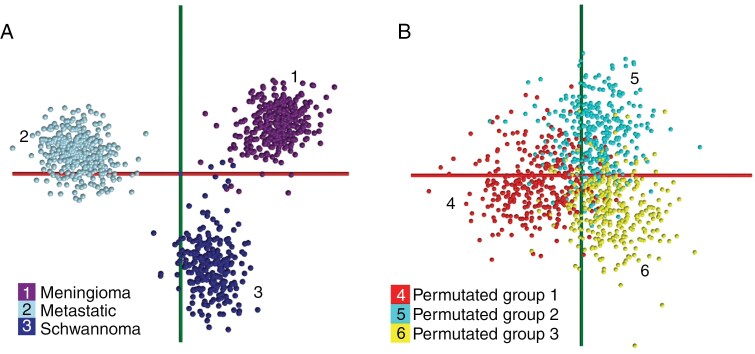
Multivariate PCA-LDA modeling of major spinal tumor types. Here we show 2 molecular models built from *n* = 122 independent patient specimens (*n* = 39 meningiomas, *n* = 41 schwannoma, and *n* = 42 metastatic tumors) generating the *n* = 959 sampling events illustrated in the plot. (A) PCA-LDA model for true annotations wherein we have grouped each tumor type into their correct pathology class as verified by a neuropathologist. (B) A permutated PCA-LDA model bearing mixed class annotations wherein each group (class) is comprised of ~ equal numbers of sampling events from all other participating classes. As can be seen, the true annotation model (panel A) exhibits a clustering of each tumor type data, supported by cross-validation results (Table S2) resulting in accuracies of 98.84% (for 20% leave-out) and 96.27% (for full group leave-out). The permutated model of Panel B on the other hand does not show any group separations, consistent with its poor cross-validation statistics (Table S3) of 41.32% (for 20% leave-out) and 35.54% (for full group leave-out).

To assess the utility of PIRL-MS analysis for accurate diagnosis of major spinal tumors, we first subjected meningiomas (most common) to molecular profiling with PIRL-MS over the mass range of 100–1000 Da where the majority of membrane and metabolic lipids (both altered during tumorigenesis) are routinely seen.^32–35,41,42^ To assess the feasibility of diagnosis solely based on PIRL-MS molecular profile (to warrant complete resection in order to reduce local recurrence), it was crucial to first establish that the PIRL-MS profile of meningiomas captured sufficient molecular information to enable their discrimination from the other two most frequently occurring spinal tumors of schwannomas and metastatic cancers, the latter class included as a control. While metastatic tumors are almost always extradural (hence, not in the formal differential diagnosis with schwannomas and meningiomas that are intradural and extramedullary), it was important to demonstrate that gross differentiation between these very different dominant classes of spinal tumors was possible with PIRL-MS. As such, this study does not utilize normal spinal cord tissue as a control and metastatic cancers are utilized to serve this function. To this end, Figure 1 shows representative 10-second PIRL-MS spectra of meningioma, schwannoma, and metastatic tumors, suggesting that their molecular profiles indeed contained mass-to-charge (*m/z*) features with characteristic abundances unique to each tumor type. To assess whether these unique fingerprints had utility in distinguishing meningiomas from the said two other types, we subjected the PIRL-MS profiles of *n* = 122 independent patient samples (*n* = 39 meningiomas, *n* = 41 schwannoma, and *n* = 42 metastatic tumors generating *n* = 959 spectra) to multivariate modeling using principal component analysis, linear discriminant analysis (PCA-LDA) with previously demonstrated utility in PIRL-MS mediated diagnosis of cancer.^32,33,35,41^ This dataset possessed average signal duration and signal intensities of 13 ± 2 seconds and (3 ± 2)· 10^6^, respectively (Table S1). Figure 2A shows the PCA-LDA “scores plot” of this dataset. In this model, separate clustering of the PIRL-MS data is seen for each of the meningioma, schwannoma, and metastatic tumor types. In this plot, we deliberately included the PIRL-MS data from multiple sampling points across each independent specimen’s surface such that the impact of intratumoral heterogeneity could be simultaneously captured in the model alongside inter-specimen heterogeneity to make the PCA-LDA model more generalizable and thus more competent in the classification of query specimens.^45^ Additionally, as shown in SI, Specimens and Histology section, the meningioma samples included in the model contained a variety of histologic subclasses across grades 1 and 2. A molecular model that includes (and thus tolerates) biological variations across various histological types, grades, and both inter- and intraspecimen heterogeneities is likely to provide a more robust prediction regardless of the local heterogeneity at the sampled site on a query specimen.^45^ Consistent with the separation (i.e. distance between clusters) shown in Figure 2A, metastatic cancers were most distinct from schwannoma or meningioma tumors. However, the latter two intradural extramedullary tumor types could also be separated from one another based on their unique PIRL-MS molecular profiles in the same multivariate PCA-LDA space. It must be noted that the metastatic cancers used in Figure 2A model originated from a variety of distantly related cancers including breast, lung, esophagus, kidney, colon, prostate, ovary, and uterus (See SI, Specimens and Histology section). Therefore, the use of metastatic tumors as a control helped us contextualize the significance of the extent of discrimination seen between schwannoma and meningioma specimen clusters in this model. In this comparison, the meningioma specimens used belonged to a variety of grade 1 and 2 and diverse histologic subtypes, including transitional, fibrous, and meningothelial (See SI, Specimens and Histology section). Therefore, the molecular model created is expected to encompass signal variations across these common subtypes, in keeping with recommendations to include a diverse set of specimens at the model-building step.^45^

### Validation of the Molecular Model for Classification of Common Spinal Tumor Types With PIRL-MS

To add more precision to the qualitative picture presented in Figure 2A, Table S2 reports the cross-validation statistics for this model. For this purpose, we used both “20%” and “full group” leave-out tests wherein a series of PCA-LDA models were built using 80% of the data and iteratively tested using the remaining 20%. Cross-validation accuracies of 98.84% and 96.27% were obtained for the 20% leave-out and the full group leave-out tests, respectively. In a 20% leave-out test, it is possible that independent measurements from the same specimen’s surface populate both model and test sets. This, however, does not happen in a full group leave-out test. While the 20% leave-out accuracies are clearly biased and thus cannot be rigorously interpreted, a comparison between the accuracy values obtained with these 2 approaches allows insights into the relative importance of inter- versus intraspecimen heterogeneities. This is important, especially for schwannomas wherein intraspecimen heterogeneity must be considered for accurate diagnosis.^46^ The concordance between these accuracy values suggests that the Figure 2A model has likely captured sufficient levels of both intraspecimen as well as interspecimen heterogeneities to allow robust recognition of an independent validation test set addressing both local heterogeneities at the sampling site as well as and interspecimen variance. In addition, the cross-validation tests yield a confusion matrix (also shown in Table S2) that details the origins of datapoints misclassified during cross-validation, which further highlights their distribution across each tumor type in the model. Table S2 results reveal that only a few (*n* = 11 out of *n* = 959 total spectra) misclassifications occurred predominantly between schwannoma and meningiomas during cross-validation.

To ensure that the *n* = 122 independent samples utilized in Figure 2A were sufficient in numbers to capture a suitable level of interspecimen biological variance enabling robust multivariate modeling, we investigated the learning curves of the model. In Figure S1, we demonstrate a saturation of the model’s cross-validation statistics at ~50% of the total data usage. This plateau is maintained all the way to 100% data usage, further validating the cohort size. Therefore, the Figure 2A model (comprised of *n* = 122 independent specimens at 100% data usage) is likely generalizable and can offer statistically meaningful classifications without the need to include additional specimens to boost its performance. To better account for population-level variance in PIRL-MS profiles; however, a larger specimen cohort is required. Capturing this biological variance is important to address potential “late-stage” non-idealities in the performance of the model when faced with previously unseen query surgical specimens outside the laboratory validation setting.^47^

To examine whether the separation between the PIRL-MS profiles of meningioma, schwannoma, and metastatic cancers seen in Figure 2A were statistically significant, we additionally created a permutated PCA-LDA model.^45^ False annotations were used to create a 3-component model with mixed-class representations in each of its 3 groups. This model (Figure 2B) did not exhibit any group separation. Consistent with this, the model’s cross-validation statistics (Table S3) were considerably poorer compared to those of the Figure 2A model with true annotations reported in Table S2 (41.32% vs 98.84% and 35.54% vs 96.27% for 20% and full group leave-out tests, respectively). This suggested that the distinctions between PIRL-MS profiles of *n* = 122 tumors shown in Figure 2A were unlikely to have been influenced by data overfitting, which can take place in supervised multivariate analyses such as PCA-LDA as recently reviewed.^45^

### Examining the Model Performance and Generalizability With Blind Sample Tests: What is the Sensitivity and Specificity of Rapid Classification of Common Spinal Tumor Types With PIRL-MS?

To validate the utility of the Figure 2A model we performed blind test predictions utilizing data previously not seen by the model. We subjected an additional set of 60 independent specimens (*n* = 20 meningioma, *n* = 20 schwannoma, and *n* = 20 metastatic tumors) to PIRL-MS profiling and classification against the model to assess model’s performance and generalizability.^45^ This assessment utilized cluster overlap (within a predetermined standard deviation) between Figure 2A model and blind test query data (for determination of class), and Mahalanobis distance measurements^48^ (for calculations of the probability of said class predictions), previously used for the determination of probability of classification in multivariate analysis.^32,33,35,41^Table S4 provides a list of expected tumor types (from hematoxylin and eosin (H&E) pathology), predicted classes (from cluster overlap of PIRL-MS spectra), alongside a probability of prediction value associated with each mass spectral prediction (from Mahalanobis distance maps). In this table, we have also listed select MS metrics such as spectral intensity and the duration of the PIRL-MS signal in seconds. A total of *n* = 487 PIRL-MS measurements were scored in this table (in a blind manner) across *n* = 60 independent specimens detailed above. The average signal duration (± 1 standard deviation) and signal intensity values were (10 ± 3) s and (4 ± 3) · 10^6^, respectively, and in line with those of the model (Table S1). Out of the *n* = 487 measurements, only n = 16 (3% of the total) sampling events were unclassifiable (outlier) and *n* = 24 (~5% of the total sampling attempts) resulted in “bad” data (defined as the signal duration of less than or equal to 3 seconds). Both of these sets were excluded from the calculations as no clinical decision will be made based on them, only affecting the duty cycle of the method. The total number of classifiable events were therefore *n* = 447, used as the basis for our calculations. Additionally, consistent with the model cross-validation results (Table S2) suggesting that a sufficient level of intraspecimen variance in signal has been captured by the Figure 2A model, 92.34% of all the classifiable *n* = 447 blind sample predictions resulted in spatially invariant (concordant) correct classification results irrespective of the sampled site across the specimen’s surface. It is worth mentioning that the major contributors to the reduction in spatially concordant classification value were 2 specimens in which none of the sampling events had been classified correctly. Nevertheless, this high concordance is encouraging for eventual clinical deployment as it suggests that robust diagnosis by a few PIRL-MS measurements using the Figure 2A model is largely possible despite intraspecimen heterogeneity. Here, only 25 measurements possessed probability in prediction values of less than 95% (underlined in Table S4), suggesting that most of the measurements yielded robust predictions with high confidence using Mahalanobis distances. The application of a 95% prediction in probable threshold thus results in a dataset of *n* = 422 sampling events across n = 60 blind specimens. The implications of these findings in terms of accuracies of predictions are discussed below.

Table 1 reformulates the blind test validation results of Table S4 (presented on a per-sampling event basis) in terms of the sensitivity and specificity for meningioma, schwannoma, and metastatic tumor diagnosis with PIRL-MS profiling based on Figure 2A model. The sensitivity and specificity values in this table are provided in 2 ways (1) without “thresholding” (ie, without taking into consideration the prediction probability from Mahalanobis distance mapping^32,33,48^) and (2) upon only considering predictions that had > 95% probability as positive hits. The unthresholded data (*n* = 447 data points) possessed an average sensitivity & specificity value of (92% ± 5%) and (96% ± 2%), respectively. Upon application of a 95% prediction probability threshold (*n* = 422 data points); however, the average sensitivity & specificity values were not drastically improved. The new values of (93% ± 5%) and (97% ± 2%) for specificity and sensitivity within the error (shown as ± standard deviation) were identical to those obtained without thresholding. This observation suggests that the Figure 2A PCA-LDA model could deliver a highly robust diagnosis of major spinal tumor types. Overall, the proposed PIRL-MS assay remained least sensitive for meningioma diagnosis (with 87.59% sensitivity for unthresholded data or 89.13% sensitivity at 95% confidence threshold) yet was still highly specific for such a discrimination with the specificity values of 97.35% and 97.54% for unthresholded and at 95% confidence threshold (these values are reported from the calculations in Table 1 without applying scientific figures of merit conventions). As shown in this table, metastatic cancers were most easily distinguished from intradural extramedullary schwannoma and meningioma tumors with sensitivity & specificity values of at least ~98%. It must be highlighted that the metastatic cancers used in this study were extradural and drastically different in chemical composition spanning diverse cancer types from several organs of origin (See SI, Specimens and Histology section). They served as a control to give context to the statistical discrimination between schwannoma and meningioma seen in Figure 2A. As shown in this figure, the statistical separation between meningioma and schwannoma is significant, and on the order of the distinctions seen with the control metastatic group which is expected to be drastically different in molecular composition.

**Table 1.** The Sensitivity and Specificity of Non-subjective Diagnosis of Major Spinal Tumor Types With PIRL-MS

	95% Threshold				No threshold	
	Predicted class				Predicted class	
	+			–	+	–
Schwannoma						
Actual	+		116	9	121	14
			92.80%		89.63%	
	–		17	280	21	291
				94.28%		93.27%
Meningioma						
Actual		+	123	15	127	18
			89.13%		87.59%	
		–	7	277	8	294
				97.54%		97.35%
Metastatic						
Actual		+	157	2	164	3
			98.74%		98.20%	
		–	2	261	6	274
				99.24%		97.86%

In this table, we have shown the sensitivity and specificity of blind classification (using Figure 2A PCA-LDA model) of the *n* = 60 independent unknown specimens equally distributed across meningioma, schwannoma, and metastatic cancers. These *n* = 60 independent samples resulted in 487 independent classification attempts (Table S4) out of which *n* = 447 resulted in classifiable data. The sensitivity and specificity calculations used the standard definition of true positive rates and true negative rates, respectively. We have presented the results of blind sample classifications across 2 probability values determined from Mahalanobis distance mapping.^48^ The raw (unthresholded) probability values are listed in Table S4 and the application of >95% probability in the prediction threshold reduced the size of the dataset to *n* = 422 as *n* = 25 sampling events (from the *n* = 477 classifiable total) possessed <95% prediction probability. PIRL-MS had the largest challenge in high sensitivity detection of meningiomas (with 89.13% sensitivity at a 95% probability threshold) yet it could deliver this diagnosis in a highly specific manner (with 97.54% specificity). Here, metastatic cancers were most robustly identifiable from PIRL-MS profiling. The +, + denotes true positive. The -,- cell shows the true negative predictions and combinations of + and—represent false positive–negative.

### Determining the Molecular Identities of the Most Important Ions Responsible for Differentiation of Common Spinal Tumors With PIRL-MS

To determine the molecular identities of the *m/z* values that enable discrimination between meningioma, schwannoma, and metastatic cancers as the top 3 most frequently occurring spinal tumors with 10-second PIRL-MS profiling, we examined the loading plots of the Figure 2A model. The loading plots (Figure S2) provide a rank order with which each *m/z* value contributes to the statistical discriminations seen. We prepared lipid extracts and attempted to determine the molecular identities of the top 100 most discriminating *m/z* values (Table S5) with high-resolution tandem mass spectrometry (MS^2^) after chromatography as previously established in PIRL-MS research.^33,35^ We were able to identify *n* = 41 of these highly discriminating *m/z* values. The molecular identities are provided in Table 2 and suggest the involvement of a variety of lipid classes such as ceramides, phosphatidylcholines, phosphatidylethanolamines, and sphingomyelins. Some of these lipids are common to cellular membranes,^50^ altered in abundance between morphologically different tumors, and many possess signaling roles with established alterations in many tumor types.^50–52^ Therefore, our observation of their involvement is not entirely surprising, and further supports their stipulated role as potential therapeutic targets.^50^ Aside from such long-term implications, the Table 2 results effectively report on a “molecular lipidomic array” that could be used for rapid discrimination between meningioma, schwannoma, and metastatic tumors through a sparse (low complexity) classification of the same unknown specimens subjected to blind test classification and reported in Table S4. The rationale for the use of these identified lipids (previously seen in other biological systems) was to reduce the chance of false discoveries mediated by artifactual exogenous signals of unknown origin potentially having contaminated our spectra as previously seen.^45^

**Table 2.** Biomarker Ions Identified by High-Resolution Tandem Mass Spectrometry With Chromatography

PIRL-MS spectral bin	Target *m/z* (PIRL-MS)	LC-MS (*m/z*)	RT (min)	Theoretical *m/z*	Error (ppm)	Adduct form	Assignment	Predicted formula (in ionic form)	Distinguishing fragments (ms/ms)
860.65	860.6646	860.6566	14.72	860.6539	−3.1	[M−H]^−^	PE(O−46:6)	C_51_H_92_NO_7_P	*m/z* 574, *m/z* 556, *m/z* 303, *m/z* 259, *m/z* 140
846.65	846.6531	846.6578	14.27	846.6595	2.0	[M + Cl]^−^	HexCer(d42:1)	C_48_H_93_NO_8_	*m/z* 810, *m/z* 648, *m/z* 630, *m/z* 408, *m/z* 392, *m/z* 367, *m/z* 366, *m/z* 263, *m/z* 237
844.65	844.6404	844.6431	12.73	844.6439	0.9	[M + Cl]^−^	HexCer(d42:2)	C_48_H_91_NO_8_	*m/z* 808, *m/z* 646, *m/z* 628, *m/z* 406, *m/z* 390, *m/z* 365, *m/z* 364, *m/z* 263, *m/z* 237
830.65	830.6498	830.6085	12.08	830.6069	−1.9	[M−H]^−^	PE(O−44:7)	C_49_H_86_NO_7_P	*m/z* 526, *m/z* 544, *m/z* 303, *m/z* 259, *m/z* 140
826.65	826.6313	826.6769	13.90	826.6778	1.1	[M−H]^−^	HexCer(d42:1(OH))	C_48_H_93_NO_9_	*m/z* 826, *m/z* 664, *m/z* 646, *m/z* 408, *m/z* 383
818.65	818.6290	818.6276	12.84	818.6282	0.7	[M + Cl]^−^	HexCer(d40:1)	C_46_H_89_NO_8_	*m/z* 782, *m/z* 620, *m/z* 380, *m/z* 364, *m/z* 339, *m/z* 338, *m/z* 263, *m/z* 237
816.55	816.5457	816.5822	11.6	816.5760	−7.6	[M + H]^−^	PS(38:1)	C_44_H_84_NO_10_P	*m/z* 816, *m/z* 729, *m/z* 465, *m/z* 447, *m/z* 417, *m/z* 311, *m/z* 281, *m/z* 152, *m/z* 79
794.55	794.5651	794.5680	9.70	794.5705	3.1	[M−CH3]^−^	PC(38:4)	C_46_H_84_NO_8_P	*m/z* 794, *m/z* 508, *m/z* 303, *m/z* 283, *m/z* 259, *m/z* 168, *m/z* 79
770.55	770.5630	770.5697	9.78	770.5705	1.0	[M−CH3]^−^	PC(36:2)	C_43_H_81_NO_8_P	*m/z* 770, *m/z* 506, *m/z* 488, *m/z* 281, *m/z* 224, *m/z* 168, *m/z* 79
768.55	768.5399	768.5540	8.76	768.5549	1.2	[M−CH3]^−^	PC(36:3)	C_44_H_82_NO_8_P	*m/z* 768, *m/z* 506, *m/z* 504, *m/z* 281, *m/z* 279, *m/z* 224, *m/z* 168, *m/z* 79
764.55	764.5359	764.5236	8.82	764.5236	0.0	[M−H]^−^	PE(38:5)	C_43_H_76_NO_8_P	*m/z* 764, *m/z* 478, *m/z* 460, *m/z* 303, *m/z* 281, *m/z* 259, *m/z* 140, *m/z* 79
750.55	750.5397	750.543	10.24	750.5443	1.7	[M + H]^−^	PE(O−38:5)	C_43_H_77_NO_7_P	*m/z* 750, *m/z* 436, *m/z* 418, *m/z* 331, *m/z* 287, *m/z 140*, *m/z* 79
746.55	746.5496	746.5135	8.91	746.5130	−0.7	[M−H]^−^	PE(O−38:7)	C_43_H_74_NO_7_P	*m/z* 746, *m/z* 436, *m/z* 418, *m/z* 327, *m/z* 283, *m/z* 140, *m/z* 79
744.55	744.5493	744.5534	9.7	744.5549	2.0	[M−CH3]^−^	PC(34:1)	C_42_H_82_NO_8_P	*m/z* 744, *m/z* 480, *m/z* 462, *m/z* 281, *m/z* 255, *m/z* 224, *m/z* 168, *m/z* 79
722.55	722.5268	722.5126	8.67	722.5130	0.6	[M−H]^−^	PE(O−36:5)	C_41_H_77_NO_7_P	*m/z* 722, *m/z* 438, *m/z* 420, *m/z* 301, *m/z* 257
718.55	718.5203	718.5391	9.59	718.5392	0.1	[M−CH3]^−^	PC(32:0)	C_40_H_80_NO_8_P	*m/z* 718, *m/z* 480, *m/z* 462m/z, *m/z* 255, *m/z* 224, *m/z* 168, *m/z* 79
709.45	709.4800	709.5060	7.19	709.5057	−0.4	[M + Cl]^−^	SM(d32:1)	C_37_H_75_N_2_O_6_P	*m/z* 588, *m/z* 570, *m/z* 421, *m/z* 168, *m/z* 79
704.55	704.5112	704.5238	8.96	704.5236	−0.3	[M−CH3]^−^	PC(31:0)	C_39_H_78_NO_8_P	*m/z* 704, *m/z* 255, *m/z* 241, *m/z* 224, *m/z* 168, *m/z* 79
698.65	698.6232	698.6223	15.01	698.6224	0.1	[M + Cl]^−^	Cer(d43:1)	C_43_H_85_ClNO_3_	*m/z* 662, *m/z* 420, *m/z* 404, *m/z* 379, *m/z* 378, *m/z* 239
698.55	698.5864	698.5563	8.70	698.5576	1.9	[M−H]^−^	HexCer(d34:1)	C_40_H_77_NO_8_	*m/z* 698, *m/z* 536, *m/z* 518, *m/z* 296, *m/z* 280, *m/z* 263, *m/z* 255, *m/z* 254, *m/z* 237, *m/z* 79
686.65	686.6032	686.6219	15.84	686.6224	0.7	[M + Cl]^−^	Cer(d42:0)	C_42_H_85_NO_3_	*m/z* 650 *m/z* 632, *m/z* 408, *m/z* 392, *m/z* 367, *m/z* 366, *m/z* 239
686.55	686.5285	686.5132	10.00	686.5130	−0.3	[M−H]^−^	PE(O−33:2)	C_38_H_74_NO_7_P	*m/z* 686, *m/z* 436, *m/z* 267, *m/z* 152, *m/z* 140, *m/z* 122, *m/z* 79
685.45	685.4817	685.5272	7.31	685.5290	2.6	[M−CH3]^−^	SM(d34:2)	C_39_H_77_N_2_O_6_P	*m/z* 614, *m/z* 596, *m/z* 447, *m/z* 168, *m/z* 79
672.55	672.5754	672.6065	15.15	672.6067	0.3	[M + Cl]^−^	Cer(d41:0)	C_41_H_83_NO_3_	*m/z* 636, *m/z* 618, *m/z* 394, *m/z* 378, *m/z* 353, *m/z* 352, *m/z* 239
668.55	668.5808	668.5752	13.08	668.5754	0.3	[M + Cl]^−^	Cer(d41:2)	C_41_H_79_NO_3_	*m/z* 632, *m/z* 406, *m/z* 392, *m/z* 390, *m/z* 376, *m/z* 365, *m/z* 364, *m/z* 351, *m/z* 350, *m/z* 249, *m/z* 237, *m/z* 223
661.45	661.4802	661.5271	7.58	661.5290	2.9	[M−CH3]^−^	SM(d32:0)	C_37_H_77_N_2_O_6_P	*m/z* 590, *m/z* 451, *m/z* 168, *m/z* 79
659.55	659.5202	659.5125	7.19	659.5134	1.4	[M−CH3]^−^	SM(d32:1)	C_37_H_75_N_2_O_6_P	*m/z* 588, *m/z* 570, *m/z* 421, *m/z* 168, *m/z* 79
658.55	658.5789	658.5911	14.44	658.5911	0.0	[M + Cl]^−^	Cer(d40:0)	C_40_H_81_NO_3_	*m/z* 622, *m/z* 604, *m/z* 380, *m/z* 364, *m/z* 339, *m/z* 338, *m/z* 265, *m/z* 239
656.55	656.5726	656.5745	13.91	656.5754	0.5	[M + Cl]^−^	Cer(d40:1)	C_40_H_79_NO_3_	*m/z* 620, *m/z* 602, *m/z* 380, *m/z* 364, *m/z* 339, *m/z* 338, *m/z* 263, *m/z* 237
654.55	654.5626	654.5583	12.38	654.5598	2.3	[M + Cl]^−^	Cer(d40:2)	C_40_H_77_NO_3_	*m/z* 618, *m/z* 406, *m/z* 390, *m/z* 378, *m/z* 365, *m/z* 364, *m/z* 362, *m/z* 337, *m/z* 336, *m/z* 263, *m/z* 237, *m/z* 235, *m/z* 209
642.55	642.5573	642.5597	13.2	642.5598	0.2	[M + Cl]^−^	Cer(d39:1)	C_39_H_77_NO_3_	*m/z* 606, *m/z* 394, *m/z* 380, *m/z* 378, *m/z* 364, *m/z* 353, *m/z* 352, *m/z* 339, *m/z* 338, *m/z* 249, *m/z* 235, *m/z*, 223, *m/z* 209
631.45	631.4893	631.4814	6.23	631.4821	1.1	[M−CH3]^−^	SM(d30:1)	C_35_H_71_N_2_O_6_P	*m/z* 168, *m/z* 79
626.55	626.5344	626.5284	11.12	626.5285	0.2	[M + Cl]^−^	Cer(d38:2)	C_38_H_73_NO_3_	*m/z* 590, *m/z* 572, *m/z* 352, *m/z* 336, *m/z* 336, *m/z* 311, *m/z* 310, *m/z* 261, *m/z* 235
598.45	598.4959	598.4965	9.76	598.4972	1.2	[M + Cl]^−^	Cer(d36:2)	C_36_H_69_NO_3_	*m/z* 562, m/z 324, *m/z* 322, *m/z* 308, *m/z* 306, *m/z* 283, *m/z* 282, *m/z* 281, *m/z* 280, *m/z* 263, *m/z* 261, *m/z* 237, *m/z* 235
588.45	588.4729	588.4750	7.74	588.4764	2.4	[M + Cl]^−^	Cer(d34:1(OH))	C_34_H_67_NO_4_	*m/z* 552, *m/z* 534, *m/z* 312, *m/z* 296, *m/z* 271, *m/z* 270, *m/z* 263, *m/z* 237
574.55	574.4844	574.4964	10.1	574.4972	1.4	[M + Cl]^−^	Cer(d34:0)	C_34_H_69_NO_3_	*m/z* 538, *m/z* 520, *m/z* 296, *m/z* 280, *m/z* 265, *m/z* 255, *m/z* 254, *m/z* 239
570.45	570.4614	570.4662	8.45	570.4659	−0.5	[M + Cl]^−^	Cer(d34:2)	C_34_H_65_NO_3_	*m/z* 534, *m/z* 296, *m/z* 280, *m/z* 261, *m/z* 255, *m/z* 254, *m/z* 235
365.35	365.3411	365.3413	8.00	365.3425	3.3	[M−H]^−^	FA 24:1	C_24_H_46_O_2_	Exact mass only
331.25	331.2614	331.2632	5.12	331.2643	3.3	[M−H]^−^	FA 22:4	C_22_H_36_O_2_	Exact mass only
329.25	329.2461	329.2482	4.54	329.2486	1.2	[M−H]^−^	FA 22:5	C_22_H_34_O_2_	Exact mass only
271.25	271.2226	271.2272	3.89	271.2279	2.6	[M−H]^−^	FA 16:0;O	C_16_H_32_O_3_	Exact mass only

From the list of top 100 most important *m/z* values provided in Table S5, a total of *n* = 41 ions were identified and are reported here from lipid extracts created from 3 representative meningioma, schwannoma, and metastatic cancers (1 each). This table lists the PIRL-MS spectral bins at 0.1 Da used to determine the Table S5 leads from loading plot analysis of Figure 2A PCA-LDA model, the target mass from PIRL-MS (locked-mass data) collected on Xevo G2-XS time-of-flight mass spectrometer), the observed LC-MS accurate mass from UPLC liquid chromatography (on Synapt G2-XS time-of-flight mass spectrometer), retention time (RT) in minutes (from UPLC analysis) as well as theoretical mass of the identified compounds (positive hits) and error in mass (observed versus theoretical) in parts per million (ppm), as well as adduct ion form, molecular assignments and diagnostic fragments observed in LipidMaps^49^ database search following protocols detailed in the materials and methods section and reported previously.^33^ These ions form a lipidomic array based on which (in Table 3) the same unknowns subjected to recognition using the entire mass spectral features of 100 to 1000 Da in Table S4 were reevaluated. This was done to avoid potential false discovery associated with the utilization of many uncharacterized mass spectral features in multivariate modeling results shown in Table 1. The ions identified in this table will form the basis for future studies to determine the molecular pathways involved in spinal cancer-type differentiations. For this, high-throughput lipidomics studies may be required to identify additional ions for a more rigorous definition of the pathways involved.

The sensitivity and specificity values for this feature-based list of classifier molecules are presented in Table 3. The predictions were made using a sparse or low complexity analysis in which the PIRL-MS signals for the identified *m/z* ions of the Table 2 molecular array were used as only classifiers. As can be seen in Table 3, for the differentiation of meningioma, schwannoma, and metastatic tumors based on the *n* = 41 identified lipid array classifiers, average sensitivity and specificity values of (93% ± 1%) and (97% ± 2%; at 95% confidence in prediction threshold) and (90% ± 2%) and (95% ± 3%; for unthresholded data) resulted. These values are comparable to those reported in Table 1 utilizing many uncharacterized *m/z* features. Encouragingly, a ~5% improvement in the sensitivity of meningioma detection at 95% confidence was seen (from 89.13% to 94.24%). Furthermore, the improvement brought about by this molecular array is not accompanied by a loss in the specificity of meningioma detection which remains at 97.76% (Table 3), virtually unchanged from 97.54% of Table 1 for the full-length *m/z* model. Here, the sensitivity and the specificity of schwannoma detection based on this molecular array are unchanged compared to Table 1 results that utilized many *m/z* features as input for dimensionality reduction. However, the molecular array of Table 2 appears to be less sensitive for metastatic cancer detection compared to the full mass range model (Table 1 results), albeit as specific. Interestingly, however, the use of this array resulted in the welcome elimination of all of the *n* = 16 unclassifiable events (Table S4). This, however, came at the cost of an increase in the number of predictions with less than 95% confidence (from *n* = 25 to *n* = 56), the clinical implications of which cannot be envisioned at the present time. The use of the said array, on the other hand, did not drastically improve the classification of unknowns 20 and 37 which were entirely misclassified. Therefore, the ~5% improvement in the sensitivity of meningioma detection discussed above are likely coincidental and due to the conversion of several unclassifiable events from unknowns 12, 14, 18 (one event each), and unknown 4 (3 events) to classifiable, and correct (re)classification of several previously misclassified data points from unknown 5 (Table S4). Here, we did not notice a drastic drop in the classification accuracies by way of sparse analysis using Table 2 ion list. This is encouraging in that when the model is generalized by way of implementation at the population level,^47^ a similar sparse analysis may enable utilization of a subset of cancer-type classifying *m/z* features least influenced by population noise,^47^ as recommended.^45^ Therefore, additional comparisons between Table 1 and Table 3 results suggest that the reduction in the predictive power of metastatic cancers (~4% drop in sensitivity at 95% confidence in prediction) seen by virtue of only using the feature list in Table 2 may overall prove to be beneficial, especially in the context of meningioma vs. schwannoma classification that is improved. Future studies are nevertheless required to establish which subset of important features are most robustly present in large population studies to reliably drive the classification. As such, we prefer to utilize sparse analysis to potentially shield against population noise that could negatively affect the performance of multivariate models that use many uncharacterized features each of which carries some level of innate experimental or biological variance or noise during classification.

**Table 3.** The Sensitivity and Specificity of Blind Sample Predictions Based on the Biomarker Molecular Array Reported in Table 2

	95% Threshold				No threshold	
	Predicted class				Predicted class	
	+			–	+	–
Schwannoma						
Actual	+		113	10	119	17
			91.87%		87.50%	
	–		15	269	26	301
				94.72%		92.05%
Meningioma						
Actual		+	131	8	139	14
			94.24%		90.85%	
		–	6	262	9	301
				97.76%		97.10%
Metastatic						
Actual		+	137	8	158	16
			94.48%		90.80%	
		–	5	257	12	277
				98.09%		95.85%

Here, the same blind specimens reported in Table S4 were subjected to the sparse (low-complexity) feature-based classification on AMX^53^ only using the *n* = 41 identified ions reported in Table 2. Compared to Table 1 results that utilized many features across the entire experimental mass range of 100–1000 Da whose identities could not be molecularly determined, this sparse analysis used the fully characterized members of the Table 2 array, resulting in average sensitivity and specificity values of (93% ± 1) % and (97% ± 2) % (at 95% confidence in prediction threshold), respectively. These values were largely comparable to the performance of the full mass range model reported in Table 1.

## Discussion, Caveats, and Future Directions

### Summary

We have shown that a molecular array comprised of *n* = 41 lipids is able to classify meningioma, schwannoma, and metastatic tumor types with average sensitivity and specificity values of (93% ± 1%) and (97% ± 2%; at 95% confidence in prediction threshold) respectively (Table 3). While these values may fall short of the stringent regulatory accuracies needed for future clinical utility,^54^ they certainly showcase the potential of PIRL-MS as a robust method for non-subjective rapid diagnosis of major spinal tumor types in the absence of intraoperative consults. We have tried to generate hypotheses to rationalize model failures utilizing histology (See SI Specimens and Histology section and Figure S3) and a variety of mass spectral analysis methods (See SI, rationalization of model failures and Figure S4). However, we have not eliminated any misclassified data from the study even if rationalizable to bias the statistics.

### Generalizability of Our Observations

**Figure 3.**
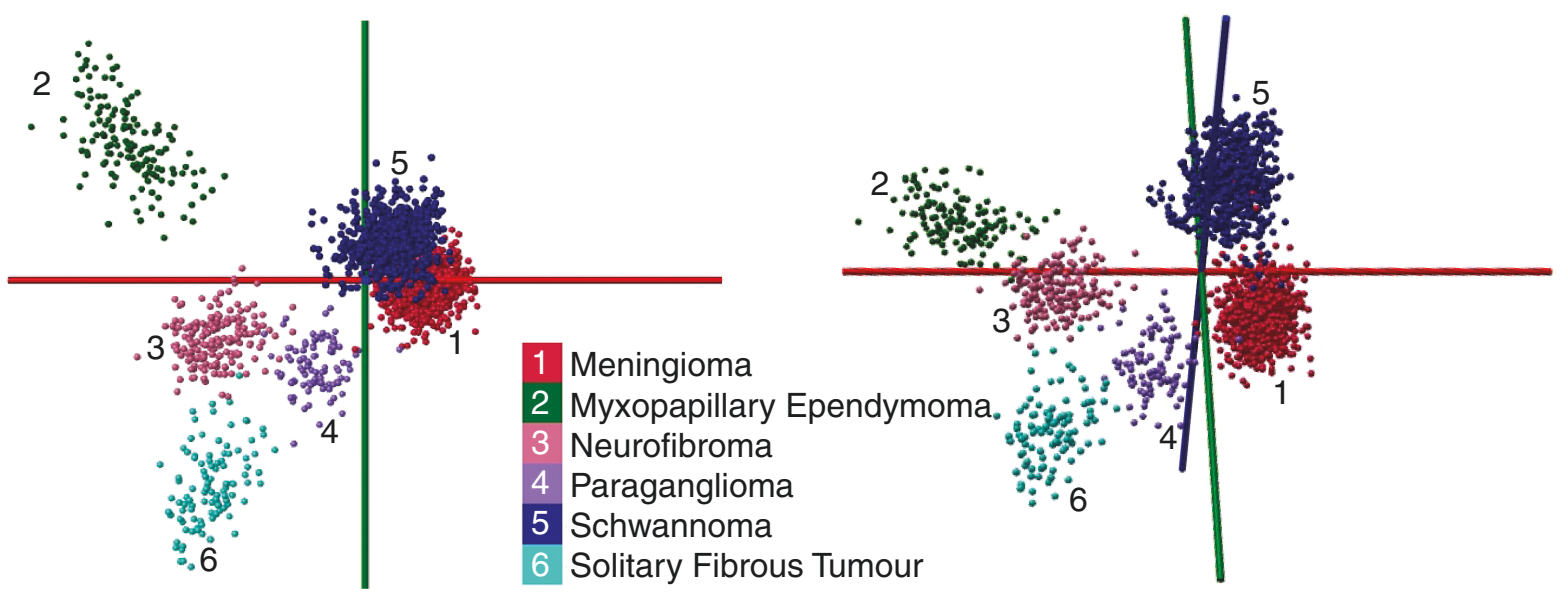
PCA-LDA models for additional spinal tumor types in the differential diagnosis. To generate this figure, we subjected *n* = 2148, PIRL-MS spectra from *n* = 257 specimens to PCA-LDA modeling: meningioma (*n* = 97), schwannoma (*n* = 106), myxopapillary ependymoma (*n* = 18), neurofibroma (*n* = 18), paraganglioma (*n* = 9), solitary fibrous tumors also known as hemangiopericytoma (*n* = 9). The cross-validation (20% and full-group leave-out) statistics for these 2 PCA-LDA models are provided in Table S6 (98.75% and 96.38%, respectively), suggesting separation between classes. The confusion matrix in Table S6 suggests minimal misclassification between meningioma and schwannoma is taking place with the additional intradural extramedullary tumors included. For clarity, 2 views of the same model are shown. It must be noted that the 3-dimensional projections shown here do not fully represent the class separations seen in other dimensions. Therefore, the cross-validation results of Table S6 must be consulted.

While including the metastatic tumors in “apparent” differential diagnosis with meningioma and schwannomas as representatives for the top 3 most frequently occurring spinal tumors may be warranted solely as a control to contextualize the obtained diagnostic power of PIRL-MS in schwannoma versus meningioma distinctions, metastatic cancers are almost always extradural. Therefore, the performance of PIRL-MS for meningioma and schwannoma differentiations must be additionally evaluated in the context of other intradural extramedullary tumors in formal differential diagnosis with them based on the WHO handbook.^7,55^ To address this caveat, Figure 3 shows a PCA-LDA model created from *n* = 257 independent spinal tumors across 6 classes of meningioma (*n* = 97), schwannoma (*n* = 106), neurofibroma (*n* = 18), paraganglioma (*n* = 9), myxopapillary ependymoma (*n* = 18), and solitary fibrous tumors, previously known as hemangiopericytoma (*n* = 9). This PCA-LDA model represents the intradural extramedullary tumors in differential diagnosis and possesses the cross-validation statistics (Table S6) of 98.75% and 96.38%, respectively, across both 20% and full-group leave-out tests. In Figure 3’s intradural extramedullary model, according to Table S6 confusion matrix, except for 2 schwannoma sampling events classifying as neurofibroma, most misclassifications were with meningiomas. In a similar vein, meningiomas are only misclassified as schwannoma, unaffected by the presence of other intradural extramedullary neoplasms. In other words, meningioma and schwannomas did not show preferential misclassifications with the newly incorporated tumor data. This likely stems from the fact that the additional tumor types added to the model were molecularly significantly distinct from meningioma and schwannoma tumor types. A caveat with this analysis, however, is that the Figure 3 dataset is highly imbalanced in terms of independent specimen numbers per class, containing rare tumors banked at much lower numbers compared to the prevalent cases. Since the utility of supervised PCA-LDA modeling using such a large dataset comprised of imbalanced data is not well established in our laboratory, we are not currently prepared to provide a more in-depth interpretation of Figure 3 (or Table S6) results beyond the summary provided above. However, in keeping with Table S6 results, a preliminary sparse analysis of the Figure 3 model using the identified n = 41 ions (Table 2) suggests reasonable prediction accuracies of >90% from cross-validation assessment (Table S7). This observation attests to the robustness of Table 2 markers discovered in a different molecular context now having shown promise in formal differential diagnosis of intradural extramedullary neoplasms. In a similar vein, these results largely validate the generalizability of the Table 2 array in performing the classification of schwannoma from meningioma in the presence of additional tumor-type data previously not seen by the model.

### Future Directions

While the accuracy value of ~ 90% reported in Table S7 is encouraging, further work is required to elucidate the molecular determinants of neurofibroma, paraganglioma myxopapillary ependymoma, and solitary fibrous tumor distinction with PIRL-MS such that further blind sample assessment could be used to assess the model’s predictive power. This assessment, however, must utilize novel data analysis methods suitable to multiclass imbalanced datasets such as Figure 3 data,^45^ and will be revisited as the next phase of this project towards a comprehensive molecular model for spinal tumor diagnosis, and a suitable classifier list of ions that could potentially meet the stringent sensitivity and specificity requirements needed for clinical decision-making. In this quest, consistent with Figure 3, Tables S6 and S7 results, however, an unsupervised clustering of our intradural extramedullary spinal tumor type dataset based on Euclidian distance measurements visualized with Uniform Manifold Approximation and Projection shows an encouraging distinction between intradural extramedullary classes of spinal tumor types analyzed in this study (Figure S5). These results are indicative of the high potential of PIRL-MS in delivering robust differential diagnosis as decision support to neuropathologists of all spinal tumor types. Here, the utilization of other data analysis methods, especially those involving unsupervised data-driven or hierarchical approaches may result in improved performance by alleviating the need to form supervised clusters comprised of distinct subclasses as done here for metastatic cancers of different organs of origin. To complement the use of novel analytics as discussed above, we will also incorporate additional intradural intramedullary neoplasms such as gliomas and lymphomas among others to assess the impact of biological variance (due to new tumor types) on the model. We envision the lipidomic array developed in this work; however, to enable personalized resections of spinal tumor types by delivering non-subjective diagnoses (potentially including molecular [pathology] differentiations), a feat that is unmatched in current standard of care methods that heavily utilize morphometric and subjective methods for intraoperative diagnosis. En route to clinical utility, the results presented here must be validated using fresh surgical specimens and the cost and footprint of the PIRL-MS system must be reduced. Early indications, nevertheless, exist that at least in murine brain model^42^ and human skin^35^ fresh and frozen specimens produce concordant and cross-classifiable PIRL-MS signatures. However, clinical trials are required to validate the utility of our models with fresh tissue, and this will be a clear next step for us. In a similar vein, currently, expert mass spectrometers are required to perform data processing. We intend to incorporate data quality checks such that non-mass spectrometrists aided by said automated quality checks could operate the platform independently. This requires mining our historic data for measures that indicate poor quality spectra and a deeper understanding of potential hardware and operational failures. As such, in this light, our observations and results herein should be considered preliminary.

## Supplementary Material

noaf047_suppl_Supplementary_Materials

## Data Availability

Data will be available upon request per institutional policies.
