# Supplementary material for: A molecular array for 10-second diagnosis of common spinal tumor types with picosecond infrared laser mass spectrometry
Source: Neuro Oncol. 2025 Feb 21;27(8):2060–72. doi: 10.1093/neuonc/noaf047 (PMC12448866; doi:10.1093/neuonc/noaf047)
Supplement: noaf047_suppl_Supplementary_Materials [file noaf047_suppl_supplementary_materials.pdf]

**A Molecular Array For 10-second Diagnosis of Common Spinal Tumour Types with Picosecond Infrared Laser Mass Spectrometry**

Alexa Fiorante[1,2], Michael Woolman[1,2], David G. Munoz[3,6], Taira Kiyota[4], Lan Anna Ye[1], Yasmine Farahmand[1], Darah Vlaminck[1,2], Francis O. Talbot[1], Sunit Das[5,6], Sorcha Kellett[6], Christine Giuffrida[6], Gelareh Zadeh[1,5], Howard J. Ginsberg [3,5,6], Ahmed Aman[4,7,#] and Arash Zarrine-Afsar [1,2,5,6,*]

[1] Princess Margaret Cancer Centre, University Health Network, 101 College Street, Toronto, ON, M5G 1L7, Canada
[2] Department of Medical Biophysics, University of Toronto, 101 College Street, Toronto, ON, M5G 1L7, Canada
[3]Department of Laboratory Medicine and Pathobiology, University of Toronto, 1 King's College Circle, Toronto, ON, M5S 1A8, Canada
[4] Ontario Institute for Cancer Research (OICR), 661 University Ave Suite 510, Toronto, ON, M5G 0A3, Canada
[5] Department of Surgery, University of Toronto, 149 College Street, Toronto, ON, M5T 1P5, Canada
[6] Keenan Research Center for Biomedical Science & the Li Ka Shing Knowledge Institute, St. Michael's Hospital, 30 Bond Street, Toronto, ON, M5B 1W8, Canada
[7] Leslie Dan, Faculty of Pharmacy, University of Toronto, 144 College St, Toronto, ON, M5S 3M2, Canada

*Corresponding author: Arash Zarrine-Afsar
Email: arash.zarrine.afsar@utoronto.ca
101 College Street, Room 7-207,
MaRS Building, Princess Margaret Cancer Research Tower, 7th floor (STTARR)
Toronto, ON M5G 1L7
TEL (Office): ++1-416-581-8473
# inquiries with respect to high-resolution analysis of lipids can be directed to AAman@oicr.on.ca

**Conflict of interest statement**

MW, HJG and AZA are inventors of soft ionization utilized in this study and are consultants with Point Surgical Inc. with financial interest.

**Author contributions statement**

*Study design:* AZA, HG, DGM, AA, TK, FOT, DV
*Acquisition, curation, analysis & interpretation of data:* AF, TK, L-AY, MW, FOT, DV, DGM, SK, CJ, YF
*Drafting the manuscript:* AZA
*Access to resources:* SD, GZ, AA, SK, CJ
*Editing the manuscript:* All authors

**Table of content**


Table S1……………………………………………………………………………………………Pages S3-S12
Table S2……………………………………………………………………………………………Page S13
Table S3……………………………………………………………………………………………Page S14
Table S4……………………………………………………………………………………………Pages S15-S20
Table S5……………………………………………………………………………………………Page S21
Table S6……………………………………………………………………………………………Page S22
Table S7……………………………………………………………………………………………Page S23
Figure S1……………………………………………………………………………………………Page S24
Figure S2……………………………………………………………………………………………Page S25
Specimens and histology…………………………………………………………………………Pages S26-S30
Figure S3……………………………………………………………………………………………Page S30
Rationalization of model failures…………………………………………………………………Pages S31-S33
Figure S4……………………………………………………………………………………………Pages S34-S35
Figure S5……………………………………………………………………………………………Pages S35
Additional information: Experimental methods…………………………………………………Pages S36-S38
References……………………………………………………………………………………………Page S39

| Specimen ID | Class | Signal Duration | Total Ion Count (TIC) |
|---|---|---|---|
| ABT SMH-118 | Schwannoma | 9 | 1.59E+06 |
| ABT SMH-118 | Schwannoma | 10 | 2.69E+06 |
| ABT SMH-118 | Schwannoma | 13 | 5.29E+06 |
| ABT SMH-121 | Schwannoma | 13 | 4.91E+06 |
| ABT SMH-121 | Schwannoma | 12 | 4.56E+06 |
| ABT SMH-121 | Schwannoma | 13 | 3.11E+06 |
| ABT SMH-121 | Schwannoma | 13 | 8.11E+06 |
| ABT SMH-121 | Schwannoma | 12 | 7.81E+06 |
| ABT SMH-121 | Schwannoma | 12 | 8.41E+06 |
| ABT SMH-121 | Schwannoma | 11 | 7.37E+06 |
| ABT SMH-122 | Schwannoma | 13 | 4.42E+06 |
| ABT SMH-122 | Schwannoma | 10 | 6.79E+06 |
| ABT SMH-122 | Schwannoma | 12 | 2.49E+06 |
| ABT SMH-122 | Schwannoma | 12 | 1.42E+06 |
| ABT SMH-122 | Schwannoma | 14 | 7.01E+06 |
| ABT SMH-122 | Schwannoma | 12 | 5.58E+06 |
| ABT SMH-122 | Schwannoma | 12 | 4.12E+06 |
| ABT SMH-122 | Schwannoma | 12 | 4.94E+06 |
| ABT SMH-122 | Schwannoma | 13 | 5.73E+06 |
| ABT SMH-122 | Schwannoma | 12 | 3.82E+06 |
| ABT SMH-123 | Schwannoma | 13 | 1.75E+06 |
| ABT SMH-123 | Schwannoma | 14 | 2.08E+06 |
| ABT SMH-123 | Schwannoma | 14 | 2.78E+06 |
| ABT SMH-123 | Schwannoma | 11 | 2.16E+06 |
| ABT SMH-123 | Schwannoma | 13 | 2.87E+06 |
| ABT SMH-123 | Schwannoma | 13 | 2.84E+06 |
| ABT SMH-123 | Schwannoma | 14 | 3.47E+06 |
| ABT SMH-123 | Schwannoma | 14 | 4.71E+06 |
| ABT SMH-125 | Schwannoma | 15 | 6.21E+05 |
| ABT SMH-126B | Schwannoma | 13 | 9.30E+06 |
| ABT SMH-126B | Schwannoma | 13 | 1.34E+07 |
| ABT SMH-126B | Schwannoma | 13 | 5.37E+06 |
| ABT SMH-126B | Schwannoma | 12 | 9.30E+06 |
| ABT SMH-126B | Schwannoma | 15 | 6.79E+06 |
| ABT SMH-126B | Schwannoma | 13 | 8.28E+06 |
| ABT SMH-126B | Schwannoma | 13 | 5.63E+06 |
| ABT SMH-126B | Schwannoma | 15 | 8.42E+06 |
| ABT SMH-128 | Schwannoma | 10 | 7.16E+06 |
| ABT SMH-128 | Schwannoma | 12 | 1.86E+07 |
| ABT SMH-128 | Schwannoma | 13 | 1.61E+07 |
| ABT SMH-129B | Schwannoma | 9 | 2.21E+06 |
| ABT SMH-129B | Schwannoma | 14 | 5.45E+06 |
| ABT SMH-131 | Schwannoma | 11 | 5.40E+06 |
| ABT SMH-131 | Schwannoma | 14 | 4.95E+06 |
| ABT SMH-131B | Schwannoma | 13 | 6.02E+06 |
| ABT SMH-131B | Schwannoma | 12 | 4.97E+06 |
| ABT SMH-131B | Schwannoma | 12 | 3.10E+06 |
| ABT SMH-131B | Schwannoma | 13 | 6.46E+06 |
| ABT SMH-133B | Schwannoma | 15 | 4.33E+06 |
| ABT SMH-133B | Schwannoma | 14 | 3.30E+06 |
| ABT SMH-133B | Schwannoma | 15 | 3.39E+06 |
| ABT SMH-133B | Schwannoma | 15 | 1.55E+06 |
| ABT SMH-133B | Schwannoma | 13 | 2.28E+06 |
| ABT SMH-133B | Schwannoma | 16 | 2.83E+06 |
| ABT SMH-133B | Schwannoma | 13 | 5.21E+06 |
| ABT SMH-133B | Schwannoma | 12 | 2.42E+06 |
| ABT SMH-133B | Schwannoma | 13 | 3.04E+06 |
| ABT SMH-133B | Schwannoma | 14 | 2.48E+06 |
| ABT SMH-133B | Schwannoma | 16 | 3.07E+06 |
| ABT SMH-133B | Schwannoma | 21 | 2.20E+06 |
| ABT SMH-134 | Schwannoma | 13 | 3.43E+06 |
| ABT SMH-134 | Schwannoma | 13 | 7.16E+06 |
| ABT SMH-134 | Schwannoma | 11 | 5.92E+06 |
| ABT SMH-134 | Schwannoma | 13 | 6.76E+06 |
| ABT SMH-134 | Schwannoma | 12 | 6.46E+06 |
| ABT SMH-134 | Schwannoma | 15 | 2.45E+06 |
| ABT SMH-134 | Schwannoma | 12 | 3.71E+06 |
| ABT SMH-136 | Schwannoma | 13 | 2.21E+06 |
| ABT SMH-136 | Schwannoma | 11 | 5.96E+05 |
| ABT SMH-136 | Schwannoma | 13 | 1.62E+06 |
| ABT SMH-136 | Schwannoma | 13 | 3.97E+06 |
| ABT SMH-136 | Schwannoma | 10 | 5.83E+06 |
| ABT SMH-136 | Schwannoma | 14 | 3.04E+06 |
| ABT SMH-136 | Schwannoma | 11 | 1.70E+06 |
| ABT SMH-137 | Schwannoma | 7 | 1.37E+06 |
| ABT SMH-137 | Schwannoma | 16 | 3.16E+06 |
| ABT SMH-139 | Schwannoma | 14 | 3.83E+06 |
| ABT SMH-139 | Schwannoma | 17 | 2.91E+06 |
| ABT SMH-139 | Schwannoma | 10 | 1.48E+06 |
| ABT SMH-139 | Schwannoma | 11 | 1.85E+06 |
| ABT SMH-139 | Schwannoma | 7 | 1.77E+06 |
| ABT SMH-139 | Schwannoma | 14 | 1.72E+06 |
| ABT SMH-139 | Schwannoma | 11 | 2.71E+06 |
| ABT SMH-141 | Schwannoma | 15 | 3.59E+06 |
| ABT SMH-141 | Schwannoma | 12 | 4.65E+06 |
| ABT SMH-141 | Schwannoma | 12 | 3.17E+06 |
| ABT SMH-141 | Schwannoma | 15 | 2.86E+06 |
| ABT SMH-141 | Schwannoma | 14 | 4.11E+06 |
| ABT SMH-142B | Schwannoma | 13 | 4.33E+06 |
| ABT SMH-142B | Schwannoma | 15 | 7.24E+06 |
| ABT SMH-142B | Schwannoma | 15 | 5.17E+06 |
| ABT SMH-142B | Schwannoma | 15 | 5.81E+06 |
| ABT SMH-142B | Schwannoma | 12 | 2.28E+06 |
| ABT SMH-142B | Schwannoma | 15 | 6.51E+06 |
| ABT SMH-142B | Schwannoma | 13 | 1.06E+07 |
| ABT SMH-142B | Schwannoma | 13 | 5.31E+06 |
| ABT SMH-142B | Schwannoma | 14 | 1.05E+07 |
| ABT SMH-142BR | Schwannoma | 13 | 6.45E+06 |
| ABT SMH-142BR | Schwannoma | 11 | 3.52E+06 |

| | | | |
|---|---|---|---|
| ABT SMH-142BR | Schwannoma | 12 | 4.68E+06 |
| ABT SMH-142BR | Schwannoma | 11 | 3.19E+06 |
| ABT SMH-142BR | Schwannoma | 15 | 5.36E+06 |
| ABT SMH-142BR | Schwannoma | 15 | 1.07E+07 |
| ABT SMH-144 | Schwannoma | 14 | 5.49E+06 |
| ABT SMH-144 | Schwannoma | 16 | 5.75E+06 |
| ABT SMH-144 | Schwannoma | 12 | 4.97E+06 |
| ABT SMH-144 | Schwannoma | 11 | 5.88E+06 |
| ABT SMH-144 | Schwannoma | 12 | 4.52E+06 |
| ABT SMH-144 | Schwannoma | 11 | 2.26E+06 |
| ABT SMH-145B | Schwannoma | 12 | 1.55E+06 |
| ABT SMH-145B | Schwannoma | 12 | 2.85E+06 |
| ABT SMH-145B | Schwannoma | 16 | 3.83E+06 |
| ABT SMH-145B | Schwannoma | 11 | 5.81E+06 |
| ABT SMH-145B | Schwannoma | 13 | 4.03E+06 |
| ABT SMH-145B | Schwannoma | 13 | 4.40E+06 |
| ABT SMH-145B | Schwannoma | 12 | 3.07E+06 |
| ABT SMH-145B | Schwannoma | 14 | 3.88E+06 |
| ABT SMH-145B | Schwannoma | 12 | 3.05E+06 |
| ABT SMH-145B | Schwannoma | 16 | 4.37E+06 |
| ABT SMH-146B | Schwannoma | 14 | 4.11E+06 |
| ABT SMH-146B | Schwannoma | 15 | 2.89E+06 |
| ABT SMH-146B | Schwannoma | 13 | 1.70E+06 |
| ABT SMH-146B | Schwannoma | 17 | 1.09E+06 |
| ABT SMH-146B | Schwannoma | 12 | 1.44E+06 |
| ABT SMH-147B | Schwannoma | 13 | 3.21E+06 |
| ABT SMH-147B | Schwannoma | 13 | 4.77E+05 |
| ABT SMH-147B | Schwannoma | 14 | 3.74E+05 |
| ABT SMH-147B | Schwannoma | 12 | 1.63E+06 |
| ABT SMH-147B | Schwannoma | 11 | 1.21E+06 |
| ABT SMH-147B | Schwannoma | 15 | 1.55E+06 |
| ABT SMH-147B | Schwannoma | 14 | 1.38E+06 |
| ABT SMH-147B | Schwannoma | 13 | 1.90E+06 |
| ABT SMH-147B | Schwannoma | 11 | 1.30E+06 |
| ABT SMH-147B | Schwannoma | 15 | 3.07E+06 |
| ABT SMH-149B | Schwannoma | 18 | 4.08E+06 |
| ABT SMH-149B | Schwannoma | 12 | 3.52E+06 |
| ABT SMH-149B | Schwannoma | 11 | 4.74E+06 |
| ABT SMH-149B | Schwannoma | 15 | 7.41E+06 |
| ABT SMH-149B | Schwannoma | 14 | 4.82E+06 |
| ABT SMH-149B | Schwannoma | 15 | 3.17E+06 |
| ABT SMH-149B | Schwannoma | 14 | 3.08E+06 |
| ABT SMH-149B | Schwannoma | 14 | 2.42E+06 |
| ABT SMH-149B | Schwannoma | 11 | 1.75E+06 |
| ABT SMH-149R | Schwannoma | 13 | 5.03E+06 |
| ABT SMH-149R | Schwannoma | 12 | 7.25E+06 |
| ABT SMH-149R | Schwannoma | 13 | 1.17E+07 |
| ABT SMH-150B | Schwannoma | 13 | 2.30E+06 |
| ABT SMH-150B | Schwannoma | 13 | 6.23E+05 |
| ABT SMH-150B | Schwannoma | 13 | 1.06E+06 |
| ABT SMH-150B | Schwannoma | 12 | 6.45E+05 |
| ABT SMH-150B | Schwannoma | 16 | 1.02E+06 |
| ABT SMH-150B | Schwannoma | 15 | 8.01E+05 |
| ABT SMH-150B | Schwannoma | 17 | 1.30E+06 |
| ABT SMH-150B | Schwannoma | 14 | 1.74E+06 |
| ABT SMH-150B | Schwannoma | 12 | 1.11E+06 |
| ABT SMH-150B | Schwannoma | 13 | 3.35E+06 |
| ABT SMH-150B | Schwannoma | 16 | 2.43E+06 |
| ABT SMH-150B | Schwannoma | 14 | 2.72E+06 |
| ABT SMH-29 | Schwannoma | 10 | 4.09E+06 |
| ABT SMH-29 | Schwannoma | 15 | 6.08E+06 |
| ABT SMH-29 | Schwannoma | 9 | 3.67E+06 |
| ABT SMH-29 | Schwannoma | 10 | 1.87E+06 |
| ABT SMH-29 | Schwannoma | 15 | 1.41E+06 |
| ABT SMH-29 | Schwannoma | 16 | 3.99E+06 |
| ABT SMH-29 | Schwannoma | 15 | 6.11E+06 |
| ABT SMH-29 | Schwannoma | 13 | 6.33E+06 |
| ABT SMH-31 | Schwannoma | 12 | 1.92E+06 |
| ABT SMH-31 | Schwannoma | 12 | 2.66E+06 |
| ABT SMH-31 | Schwannoma | 11 | 7.83E+05 |
| ABT SMH-31 | Schwannoma | 10 | 9.26E+05 |
| ABT SMH-31 | Schwannoma | 11 | 1.58E+06 |
| ABT SMH-31 | Schwannoma | 13 | 3.00E+06 |
| ABT SMH-31 | Schwannoma | 12 | 2.03E+06 |
| ABT SMH-31 | Schwannoma | 12 | 2.79E+06 |
| ABT SMH-33 | Schwannoma | 11 | 2.86E+06 |
| ABT SMH-33 | Schwannoma | 12 | 5.91E+06 |
| ABT SMH-33 | Schwannoma | 12 | 6.87E+06 |
| ABT SMH-33 | Schwannoma | 13 | 1.60E+06 |
| ABT SMH-33 | Schwannoma | 13 | 3.62E+06 |
| ABT SMH-33 | Schwannoma | 13 | 5.87E+06 |
| ABT SMH-33 | Schwannoma | 12 | 3.58E+06 |
| ABT SMH-34A | Schwannoma | 12 | 2.19E+06 |
| ABT SMH-34A | Schwannoma | 10 | 1.92E+06 |
| ABT SMH-34A | Schwannoma | 11 | 2.22E+06 |
| ABT SMH-34A | Schwannoma | 13 | 3.74E+06 |
| ABT SMH-34A | Schwannoma | 12 | 4.38E+06 |
| ABT SMH-34A | Schwannoma | 12 | 4.75E+06 |
| ABT SMH-34A | Schwannoma | 12 | 6.03E+06 |
| ABT SMH-35 | Schwannoma | 13 | 1.15E+06 |
| ABT SMH-35 | Schwannoma | 10 | 1.45E+06 |
| ABT SMH-35 | Schwannoma | 11 | 2.24E+06 |
| ABT SMH-35 | Schwannoma | 12 | 3.65E+06 |
| ABT SMH-38 | Schwannoma | 10 | 3.26E+06 |
| ABT SMH-38 | Schwannoma | 11 | 2.43E+06 |
| ABT SMH-38 | Schwannoma | 9 | 7.75E+05 |
| ABT SMH-38 | Schwannoma | 14 | 2.82E+06 |
| ABT SMH-38 | Schwannoma | 17 | 5.90E+06 |
| ABT SMH-38 | Schwannoma | 12 | 3.69E+06 |
| ABT SMH-38 | Schwannoma | 12 | 4.92E+06 |

| | | | |
|---|---|---|---|
| ABT SMH-39 | Schwannoma | 16 | 9.16E+05 |
| ABT SMH-39 | Schwannoma | 27 | 2.88E+06 |
| ABT SMH-39 | Schwannoma | 14 | 1.05E+06 |
| ABT SMH-39 | Schwannoma | 15 | 3.95E+06 |
| ABT SMH-39 | Schwannoma | 13 | 3.65E+06 |
| ABT SMH-39 | Schwannoma | 13 | 1.32E+06 |
| ABT SMH-39 | Schwannoma | 19 | 4.01E+06 |
| ABT SMH-39 | Schwannoma | 14 | 1.84E+06 |
| ABT SMH-39 | Schwannoma | 14 | 4.07E+06 |
| ABT SMH-39 | Schwannoma | 17 | 3.04E+06 |
| ABT SMH-42 | Schwannoma | 13 | 3.45E+06 |
| ABT SMH-42 | Schwannoma | 11 | 3.15E+06 |
| ABT SMH-42 | Schwannoma | 14 | 3.26E+06 |
| ABT SMH-42 | Schwannoma | 13 | 2.13E+06 |
| ABT SMH-42 | Schwannoma | 17 | 5.00E+06 |
| ABT SMH-42 | Schwannoma | 13 | 9.24E+06 |
| ABT SMH-42 | Schwannoma | 13 | 7.22E+06 |
| ABT SMH-43 | Schwannoma | 16 | 1.44E+06 |
| ABT SMH-43 | Schwannoma | 15 | 1.66E+06 |
| ABT SMH-43 | Schwannoma | 13 | 2.65E+06 |
| ABT SMH-43 | Schwannoma | 14 | 1.53E+06 |
| ABT SMH-43 | Schwannoma | 12 | 1.65E+06 |
| ABT SMH-43 | Schwannoma | 14 | 1.80E+06 |
| ABT SMH-43 | Schwannoma | 15 | 4.86E+06 |
| ABT SMH-84 | Schwannoma | 10 | 1.29E+06 |
| ABT SMH-84 | Schwannoma | 12 | 4.33E+06 |
| ABT SMH-84 | Schwannoma | 10 | 2.17E+06 |
| ABT SMH-84 | Schwannoma | 12 | 1.20E+06 |
| ABT SMH-84 | Schwannoma | 12 | 2.87E+06 |
| ABT SMH-84 | Schwannoma | 14 | 1.92E+06 |
| ABT SMH-84 | Schwannoma | 11 | 2.82E+06 |
| ABT SMH-85 | Schwannoma | 13 | 1.48E+07 |
| ABT SMH-85 | Schwannoma | 14 | 1.12E+07 |
| ABT SMH-87 | Schwannoma | 12 | 5.01E+06 |
| ABT SMH-87 | Schwannoma | 10 | 6.09E+06 |
| ABT SMH-87 | Schwannoma | 13 | 8.52E+06 |
| ABT SMH-87 | Schwannoma | 13 | 7.59E+06 |
| ABT SMH-89 | Schwannoma | 15 | 4.98E+06 |
| ABT SMH-89 | Schwannoma | 13 | 7.06E+06 |
| ABT SMH-89 | Schwannoma | 13 | 9.01E+06 |
| ABT SMH-89 | Schwannoma | 14 | 5.33E+06 |
| ABT SMH-89 | Schwannoma | 14 | 3.51E+06 |
| ABT SMH-90 | Schwannoma | 11 | 5.10E+06 |
| ABT SMH-90 | Schwannoma | 10 | 2.87E+06 |
| ABT SMH-90 | Schwannoma | 9 | 2.78E+06 |
| ABT SMH-90 | Schwannoma | 11 | 3.36E+06 |
| ABT SMH-90 | Schwannoma | 13 | 4.41E+06 |
| ABT SMH-90 | Schwannoma | 11 | 1.43E+06 |
| ABT SMH-90 | Schwannoma | 14 | 6.73E+06 |
| ABT SMH-90 | Schwannoma | 10 | 8.34E+05 |
| ABT SMH-90 | Schwannoma | 13 | 4.37E+06 |
| ABT SMH-92 | Schwannoma | 9 | 4.98E+06 |
| ABT SMH-92 | Schwannoma | 9 | 1.58E+06 |
| ABT SMH-92 | Schwannoma | 16 | 3.21E+06 |
| ABT SMH-92 | Schwannoma | 13 | 2.86E+06 |
| ABT SMH-92 | Schwannoma | 13 | 3.37E+06 |
| ABT SMH-93 | Schwannoma | 9 | 1.54E+06 |
| ABT SMH-93 | Schwannoma | 11 | 2.21E+06 |
| ABT SMH-93 | Schwannoma | 11 | 3.14E+06 |
| ABT SMH-93 | Schwannoma | 11 | 4.11E+06 |
| ABT SMH-104 | Meningioma | 15 | 2.25E+06 |
| ABT SMH-104 | Meningioma | 11 | 5.26E+06 |
| ABT SMH-104 | Meningioma | 13 | 3.45E+06 |
| ABT SMH-104 | Meningioma | 13 | 4.45E+06 |
| ABT SMH-104 | Meningioma | 14 | 2.15E+06 |
| ABT SMH-104 | Meningioma | 13 | 2.80E+06 |
| ABT SMH-104 | Meningioma | 14 | 6.68E+06 |
| ABT SMH-104 | Meningioma | 18 | 3.50E+06 |
| ABT SMH-104 | Meningioma | 12 | 1.96E+06 |
| ABT SMH-105 | Meningioma | 11 | 4.61E+06 |
| ABT SMH-105 | Meningioma | 10 | 4.92E+06 |
| ABT SMH-105 | Meningioma | 13 | 5.94E+06 |
| ABT SMH-105 | Meningioma | 14 | 6.34E+06 |
| ABT SMH-105 | Meningioma | 14 | 7.35E+06 |
| ABT SMH-105 | Meningioma | 12 | 7.22E+06 |
| ABT SMH-105 | Meningioma | 15 | 9.44E+06 |
| ABT SMH-105 | Meningioma | 13 | 7.69E+06 |
| ABT SMH-107 | Meningioma | 14 | 1.01E+07 |
| ABT SMH-107 | Meningioma | 13 | 9.50E+06 |
| ABT SMH-107 | Meningioma | 13 | 6.88E+06 |
| ABT SMH-108 | Meningioma | 15 | 2.92E+06 |
| ABT SMH-108 | Meningioma | 13 | 2.97E+06 |
| ABT SMH-108 | Meningioma | 12 | 4.74E+06 |
| ABT SMH-108 | Meningioma | 12 | 3.76E+06 |
| ABT SMH-108 | Meningioma | 18 | 7.30E+05 |
| ABT SMH-108 | Meningioma | 22 | 3.89E+06 |
| ABT SMH-109 | Meningioma | 18 | 1.43E+06 |
| ABT SMH-109 | Meningioma | 16 | 2.66E+06 |
| ABT SMH-109 | Meningioma | 13 | 2.39E+06 |
| ABT SMH-109 | Meningioma | 17 | 3.01E+06 |
| ABT SMH-109 | Meningioma | 14 | 2.91E+06 |
| ABT SMH-109 | Meningioma | 15 | 2.91E+06 |
| ABT SMH-109 | Meningioma | 13 | 1.60E+06 |
| ABT SMH-109 | Meningioma | 10 | 6.82E+05 |
| ABT SMH-109 | Meningioma | 17 | 1.10E+06 |
| ABT SMH-109 | Meningioma | 19 | 2.10E+06 |
| ABT SMH-109 | Meningioma | 19 | 2.36E+06 |
| ABT SMH-110 | Meningioma | 13 | 3.26E+06 |
| ABT SMH-110 | Meningioma | 14 | 9.44E+06 |
| ABT SMH-110 | Meningioma | 11 | 4.76E+06 |

| | | | |
|---|---|---|---|
| ABT SMH-110 | Meningioma | 12 | 6.13E+06 |
| ABT SMH-111 | Meningioma | 15 | 5.63E+06 |
| ABT SMH-111 | Meningioma | 13 | 2.95E+06 |
| ABT SMH-111 | Meningioma | 18 | 3.32E+06 |
| ABT SMH-111 | Meningioma | 13 | 2.07E+06 |
| ABT SMH-111 | Meningioma | 17 | 5.29E+06 |
| ABT SMH-111 | Meningioma | 14 | 3.00E+06 |
| ABT SMH-111 | Meningioma | 19 | 2.71E+06 |
| ABT SMH-112 | Meningioma | 16 | 3.95E+06 |
| ABT SMH-112 | Meningioma | 13 | 3.24E+06 |
| ABT SMH-112 | Meningioma | 13 | 2.31E+06 |
| ABT SMH-112 | Meningioma | 16 | 2.68E+06 |
| ABT SMH-112 | Meningioma | 17 | 3.87E+06 |
| ABT SMH-112 | Meningioma | 16 | 5.45E+06 |
| ABT SMH-112 | Meningioma | 14 | 1.61E+06 |
| ABT SMH-112 | Meningioma | 16 | 2.71E+06 |
| ABT SMH-113 | Meningioma | 14 | 1.75E+06 |
| ABT SMH-113 | Meningioma | 12 | 1.40E+06 |
| ABT SMH-113 | Meningioma | 10 | 1.56E+06 |
| ABT SMH-113 | Meningioma | 10 | 6.37E+05 |
| ABT SMH-113 | Meningioma | 12 | 1.48E+06 |
| ABT SMH-113 | Meningioma | 11 | 1.28E+06 |
| ABT SMH-113 | Meningioma | 12 | 3.15E+06 |
| ABT SMH-113 | Meningioma | 13 | 9.38E+05 |
| ABT SMH-113 | Meningioma | 18 | 4.55E+06 |
| ABT SMH-113 | Meningioma | 11 | 1.91E+06 |
| ABT SMH-154 | Meningioma | 14 | 3.65E+06 |
| ABT SMH-154 | Meningioma | 12 | 5.86E+06 |
| ABT SMH-154 | Meningioma | 15 | 6.93E+05 |
| ABT SMH-154 | Meningioma | 14 | 6.38E+06 |
| ABT SMH-154 | Meningioma | 16 | 3.81E+06 |
| ABT SMH-154 | Meningioma | 17 | 3.24E+06 |
| ABT SMH-154 | Meningioma | 14 | 3.39E+06 |
| ABT SMH-154 | Meningioma | 14 | 4.07E+06 |
| ABT SMH-157 | Meningioma | 13 | 4.94E+06 |
| ABT SMH-157 | Meningioma | 14 | 3.76E+06 |
| ABT SMH-157 | Meningioma | 10 | 1.57E+06 |
| ABT SMH-157 | Meningioma | 14 | 3.64E+06 |
| ABT SMH-157 | Meningioma | 13 | 4.51E+06 |
| ABT SMH-157 | Meningioma | 14 | 2.64E+06 |
| ABT SMH-157 | Meningioma | 13 | 1.84E+06 |
| ABT SMH-157 | Meningioma | 15 | 2.21E+06 |
| ABT SMH-157 | Meningioma | 11 | 3.05E+06 |
| ABT SMH-158 | Meningioma | 11 | 3.28E+06 |
| ABT SMH-158 | Meningioma | 14 | 8.98E+05 |
| ABT SMH-158 | Meningioma | 14 | 1.05E+06 |
| ABT SMH-158 | Meningioma | 10 | 2.80E+06 |
| ABT SMH-158 | Meningioma | 11 | 2.98E+06 |
| ABT SMH-158 | Meningioma | 11 | 2.09E+06 |
| ABT SMH-158 | Meningioma | 7 | 7.35E+05 |
| ABT SMH-158 | Meningioma | 15 | 6.15E+06 |
| ABT SMH-158 | Meningioma | 14 | 5.43E+06 |
| ABT SMH-158 | Meningioma | 12 | 2.35E+06 |
| ABT SMH-164 | Meningioma | 12 | 7.76E+06 |
| ABT SMH-164 | Meningioma | 10 | 5.48E+06 |
| ABT SMH-164 | Meningioma | 12 | 7.12E+05 |
| ABT SMH-164 | Meningioma | 13 | 6.48E+06 |
| ABT SMH-164 | Meningioma | 16 | 3.66E+06 |
| ABT SMH-164 | Meningioma | 14 | 2.46E+06 |
| ABT SMH-164 | Meningioma | 13 | 2.19E+06 |
| ABT SMH-164 | Meningioma | 14 | 4.90E+06 |
| ABT SMH-164 | Meningioma | 12 | 4.10E+06 |
| ABT SMH-164 | Meningioma | 13 | 2.41E+06 |
| ABT SMH-166 | Meningioma | 13 | 1.89E+06 |
| ABT SMH-166 | Meningioma | 12 | 3.32E+06 |
| ABT SMH-166 | Meningioma | 14 | 2.30E+06 |
| ABT SMH-166 | Meningioma | 15 | 3.49E+06 |
| ABT SMH-166 | Meningioma | 13 | 1.99E+06 |
| ABT SMH-166 | Meningioma | 16 | 2.75E+06 |
| ABT SMH-166 | Meningioma | 15 | 3.40E+06 |
| ABT SMH-166 | Meningioma | 14 | 2.22E+06 |
| ABT SMH-166 | Meningioma | 15 | 3.04E+06 |
| ABT SMH-167 | Meningioma | 13 | 2.61E+06 |
| ABT SMH-167 | Meningioma | 12 | 4.27E+06 |
| ABT SMH-167 | Meningioma | 12 | 4.47E+06 |
| ABT SMH-167 | Meningioma | 13 | 7.27E+06 |
| ABT SMH-167 | Meningioma | 14 | 6.07E+06 |
| ABT SMH-167 | Meningioma | 13 | 7.18E+06 |
| ABT SMH-167 | Meningioma | 14 | 7.55E+06 |
| ABT SMH-167 | Meningioma | 12 | 4.79E+06 |
| ABT SMH-168 | Meningioma | 16 | 3.05E+06 |
| ABT SMH-168 | Meningioma | 14 | 3.08E+06 |
| ABT SMH-168 | Meningioma | 10 | 1.78E+06 |
| ABT SMH-168 | Meningioma | 10 | 2.38E+06 |
| ABT SMH-168 | Meningioma | 16 | 7.65E+06 |
| ABT SMH-168 | Meningioma | 13 | 4.72E+06 |
| ABT SMH-168 | Meningioma | 14 | 4.17E+06 |
| ABT SMH-168 | Meningioma | 16 | 7.31E+06 |
| ABT SMH-168 | Meningioma | 8 | 2.02E+06 |
| ABT SMH-173 | Meningioma | 15 | 3.05E+06 |
| ABT SMH-173 | Meningioma | 11 | 1.07E+06 |
| ABT SMH-173 | Meningioma | 13 | 2.17E+06 |
| ABT SMH-173 | Meningioma | 13 | 3.07E+06 |
| ABT SMH-173 | Meningioma | 14 | 5.54E+06 |
| ABT SMH-173 | Meningioma | 13 | 1.49E+06 |
| ABT SMH-173 | Meningioma | 10 | 7.45E+05 |
| ABT SMH-173 | Meningioma | 12 | 1.61E+06 |
| ABT SMH-173 | Meningioma | 10 | 2.45E+06 |
| ABT SMH-173 | Meningioma | 9 | 1.85E+06 |
| ABT SMH-173 | Meningioma | 14 | 5.77E+06 |

| | | | |
|---|---|---|---|
| ABT SMH-173 | Meningioma | 14 | 4.27E+06 |
| ABT SMH-174 | Meningioma | 13 | 4.05E+06 |
| ABT SMH-174 | Meningioma | 11 | 2.72E+06 |
| ABT SMH-174 | Meningioma | 15 | 5.70E+06 |
| ABT SMH-174 | Meningioma | 13 | 3.31E+06 |
| ABT SMH-174 | Meningioma | 14 | 3.80E+06 |
| ABT SMH-174 | Meningioma | 12 | 2.97E+06 |
| ABT SMH-174 | Meningioma | 12 | 3.55E+06 |
| ABT SMH-174 | Meningioma | 11 | 3.91E+06 |
| ABT SMH-174 | Meningioma | 11 | 2.14E+06 |
| ABT SMH-174 | Meningioma | 11 | 3.57E+06 |
| ABT SMH-174 | Meningioma | 13 | 2.30E+06 |
| ABT SMH-174 | Meningioma | 13 | 3.00E+06 |
| ABT SMH-175 | Meningioma | 15 | 2.70E+06 |
| ABT SMH-175 | Meningioma | 16 | 1.36E+06 |
| ABT SMH-175 | Meningioma | 13 | 1.67E+06 |
| ABT SMH-175 | Meningioma | 13 | 4.57E+06 |
| ABT SMH-175 | Meningioma | 13 | 7.62E+06 |
| ABT SMH-175 | Meningioma | 14 | 6.33E+06 |
| ABT SMH-175 | Meningioma | 11 | 3.72E+06 |
| ABT SMH-176 | Meningioma | 14 | 6.36E+06 |
| ABT SMH-176 | Meningioma | 12 | 3.67E+06 |
| ABT SMH-176 | Meningioma | 13 | 4.61E+06 |
| ABT SMH-176 | Meningioma | 11 | 1.51E+06 |
| ABT SMH-176 | Meningioma | 13 | 4.23E+06 |
| ABT SMH-176 | Meningioma | 9 | 2.12E+06 |
| ABT SMH-176 | Meningioma | 13 | 3.62E+06 |
| ABT SMH-176 | Meningioma | 11 | 3.08E+06 |
| ABT SMH-184 | Meningioma | 11 | 1.59E+06 |
| ABT SMH-184 | Meningioma | 14 | 3.37E+06 |
| ABT SMH-184 | Meningioma | 12 | 1.93E+06 |
| ABT SMH-184 | Meningioma | 13 | 1.76E+06 |
| ABT SMH-184 | Meningioma | 13 | 1.22E+06 |
| ABT SMH-184 | Meningioma | 14 | 1.02E+06 |
| ABT SMH-184 | Meningioma | 12 | 1.96E+06 |
| ABT SMH-184 | Meningioma | 13 | 1.85E+06 |
| ABT SMH-184 | Meningioma | 16 | 6.14E+05 |
| ABT SMH-184 | Meningioma | 11 | 1.41E+06 |
| ABT SMH-188 | Meningioma | 13 | 9.23E+06 |
| ABT SMH-188 | Meningioma | 4 | 1.41E+06 |
| ABT SMH-188 | Meningioma | 13 | 6.45E+06 |
| ABT SMH-188 | Meningioma | 12 | 6.02E+06 |
| ABT SMH-188 | Meningioma | 12 | 6.72E+06 |
| ABT SMH-188 | Meningioma | 12 | 5.12E+06 |
| ABT SMH-188 | Meningioma | 12 | 7.47E+06 |
| ABT SMH-189 | Meningioma | 11 | 5.93E+06 |
| ABT SMH-189 | Meningioma | 14 | 7.92E+06 |
| ABT SMH-189 | Meningioma | 14 | 8.41E+06 |
| ABT SMH-189 | Meningioma | 14 | 8.03E+06 |
| ABT SMH-189 | Meningioma | 14 | 9.85E+06 |
| ABT SMH-189 | Meningioma | 13 | 9.95E+06 |
| ABT SMH-189 | Meningioma | 14 | 4.45E+06 |
| ABT SMH-190 | Meningioma | 11 | 4.10E+06 |
| ABT SMH-190 | Meningioma | 13 | 7.16E+06 |
| ABT SMH-190 | Meningioma | 12 | 3.03E+06 |
| ABT SMH-190 | Meningioma | 12 | 7.10E+06 |
| ABT SMH-190 | Meningioma | 12 | 6.06E+06 |
| ABT SMH-190 | Meningioma | 13 | 4.74E+06 |
| ABT SMH-190 | Meningioma | 12 | 2.74E+06 |
| ABT SMH-190 | Meningioma | 12 | 3.81E+06 |
| ABT SMH-191 | Meningioma | 14 | 4.90E+06 |
| ABT SMH-191 | Meningioma | 13 | 6.53E+06 |
| ABT SMH-191 | Meningioma | 11 | 2.78E+06 |
| ABT SMH-191 | Meningioma | 12 | 5.49E+06 |
| ABT SMH-59 | Meningioma | 15 | 3.04E+06 |
| ABT SMH-59 | Meningioma | 12 | 4.03E+06 |
| ABT SMH-59 | Meningioma | 19 | 1.46E+06 |
| ABT SMH-59 | Meningioma | 14 | 3.81E+06 |
| ABT SMH-59 | Meningioma | 16 | 2.69E+06 |
| ABT SMH-59 | Meningioma | 15 | 6.99E+06 |
| ABT SMH-59 | Meningioma | 11 | 2.89E+06 |
| ABT SMH-59 | Meningioma | 14 | 6.72E+06 |
| ABT SMH-59 | Meningioma | 15 | 2.88E+06 |
| ABT SMH-59 | Meningioma | 21 | 6.92E+06 |
| ABT SMH-60 | Meningioma | 14 | 5.52E+06 |
| ABT SMH-60 | Meningioma | 11 | 2.83E+06 |
| ABT SMH-60 | Meningioma | 11 | 4.15E+06 |
| ABT SMH-60 | Meningioma | 12 | 2.10E+06 |
| ABT SMH-60 | Meningioma | 14 | 1.11E+06 |
| ABT SMH-60 | Meningioma | 13 | 6.29E+06 |
| ABT SMH-60 | Meningioma | 11 | 4.51E+06 |
| ABT SMH-60 | Meningioma | 13 | 4.94E+06 |
| ABT SMH-61 | Meningioma | 7 | 1.90E+06 |
| ABT SMH-61 | Meningioma | 13 | 4.51E+06 |
| ABT SMH-61 | Meningioma | 13 | 7.29E+06 |
| ABT SMH-61 | Meningioma | 12 | 5.74E+06 |
| ABT SMH-61 | Meningioma | 12 | 9.07E+06 |
| ABT SMH-61 | Meningioma | 12 | 9.24E+06 |
| ABT SMH-61 | Meningioma | 12 | 9.04E+06 |
| ABT SMH-61 | Meningioma | 12 | 8.70E+06 |
| ABT SMH-62 | Meningioma | 13 | 2.03E+06 |
| ABT SMH-62 | Meningioma | 13 | 4.22E+06 |
| ABT SMH-62 | Meningioma | 13 | 3.13E+06 |
| ABT SMH-62 | Meningioma | 15 | 4.45E+06 |
| ABT SMH-62 | Meningioma | 12 | 3.25E+06 |
| ABT SMH-62 | Meningioma | 13 | 2.09E+06 |
| ABT SMH-62 | Meningioma | 13 | 2.16E+06 |
| ABT SMH-62 | Meningioma | 13 | 2.08E+06 |
| ABT SMH-62 | Meningioma | 15 | 1.96E+06 |
| ABT SMH-62 | Meningioma | 13 | 1.57E+06 |

| | | | |
|---|---|---|---|
| ABT SMH-64 | Meningioma | 11 | 1.54E+06 |
| ABT SMH-64 | Meningioma | 13 | 2.81E+06 |
| ABT SMH-64 | Meningioma | 12 | 1.13E+06 |
| ABT SMH-64 | Meningioma | 14 | 6.27E+05 |
| ABT SMH-64 | Meningioma | 12 | 5.26E+05 |
| ABT SMH-64 | Meningioma | 13 | 3.38E+06 |
| ABT SMH-64 | Meningioma | 13 | 3.20E+06 |
| ABT SMH-64 | Meningioma | 12 | 4.07E+06 |
| ABT SMH-64 | Meningioma | 12 | 2.70E+06 |
| ABT SMH-64 | Meningioma | 13 | 1.37E+06 |
| ABT SMH-64 | Meningioma | 14 | 2.63E+06 |
| ABT SMH-65 | Meningioma | 14 | 2.09E+06 |
| ABT SMH-65 | Meningioma | 13 | 2.07E+06 |
| ABT SMH-65 | Meningioma | 11 | 2.26E+06 |
| ABT SMH-65 | Meningioma | 15 | 7.39E+05 |
| ABT SMH-65 | Meningioma | 14 | 9.52E+05 |
| ABT SMH-65 | Meningioma | 15 | 1.22E+06 |
| ABT SMH-65 | Meningioma | 15 | 6.35E+05 |
| ABT SMH-65 | Meningioma | 15 | 1.66E+06 |
| ABT SMH-65 | Meningioma | 14 | 9.49E+05 |
| ABT SMH-65 | Meningioma | 12 | 1.42E+06 |
| ABT SMH-66 | Meningioma | 12 | 2.39E+06 |
| ABT SMH-66 | Meningioma | 11 | 1.74E+06 |
| ABT SMH-66 | Meningioma | 10 | 6.07E+05 |
| ABT SMH-66 | Meningioma | 9 | 6.82E+05 |
| ABT SMH-66 | Meningioma | 10 | 4.22E+05 |
| ABT SMH-66 | Meningioma | 13 | 2.15E+06 |
| ABT SMH-66 | Meningioma | 12 | 9.41E+05 |
| ABT SMH-66 | Meningioma | 12 | 2.43E+06 |
| ABT SMH-67 | Meningioma | 12 | 6.41E+06 |
| ABT SMH-67 | Meningioma | 14 | 6.83E+06 |
| ABT SMH-67 | Meningioma | 13 | 1.18E+06 |
| ABT SMH-67 | Meningioma | 12 | 5.41E+06 |
| ABT SMH-67 | Meningioma | 11 | 5.19E+06 |
| ABT SMH-67 | Meningioma | 12 | 5.07E+06 |
| ABT SMH-67 | Meningioma | 13 | 3.93E+06 |
| ABT SMH-67 | Meningioma | 10 | 1.73E+06 |
| ABT SMH-67 | Meningioma | 11 | 1.14E+06 |
| ABT SMH-68 | Meningioma | 10 | 2.35E+06 |
| ABT SMH-68 | Meningioma | 13 | 2.94E+06 |
| ABT SMH-68 | Meningioma | 13 | 3.89E+06 |
| ABT SMH-68 | Meningioma | 14 | 1.48E+06 |
| ABT SMH-68 | Meningioma | 12 | 1.36E+06 |
| ABT SMH-68 | Meningioma | 12 | 1.95E+06 |
| ABT SMH-68 | Meningioma | 11 | 1.72E+06 |
| ABT SMH-68 | Meningioma | 12 | 2.00E+06 |
| ABT SMH-68 | Meningioma | 11 | 1.34E+06 |
| ABT SMH-68 | Meningioma | 14 | 1.55E+06 |
| ABT SMH-68 | Meningioma | 10 | 1.64E+06 |
| ABT SMH-68 | Meningioma | 13 | 2.03E+06 |
| ABT SMH-69 | Meningioma | 13 | 3.34E+06 |
| ABT SMH-69 | Meningioma | 12 | 3.44E+06 |
| ABT SMH-69 | Meningioma | 12 | 2.30E+06 |
| ABT SMH-69 | Meningioma | 13 | 6.65E+06 |
| ABT SMH-69 | Meningioma | 11 | 5.87E+06 |
| ABT SMH-69 | Meningioma | 12 | 4.79E+06 |
| ABT SMH-70 | Meningioma | 13 | 8.58E+06 |
| ABT SMH-70 | Meningioma | 14 | 5.89E+06 |
| ABT SMH-70 | Meningioma | 12 | 9.90E+06 |
| ABT SMH-71 | Meningioma | 12 | 2.80E+06 |
| ABT SMH-71 | Meningioma | 12 | 2.55E+06 |
| ABT SMH-71 | Meningioma | 13 | 1.64E+06 |
| ABT SMH-71 | Meningioma | 13 | 1.38E+06 |
| ABT SMH-71 | Meningioma | 15 | 2.63E+06 |
| ABT SMH-71 | Meningioma | 12 | 2.56E+06 |
| ABT SMH-71 | Meningioma | 12 | 1.86E+06 |
| ABT SMH-72 | Meningioma | 11 | 1.53E+06 |
| ABT SMH-72 | Meningioma | 12 | 2.81E+06 |
| ABT SMH-72 | Meningioma | 12 | 1.83E+06 |
| ABT SMH-72 | Meningioma | 13 | 2.64E+06 |
| ABT SMH-72 | Meningioma | 14 | 1.92E+06 |
| ABT SMH-72 | Meningioma | 13 | 2.65E+06 |
| ABT SMH-72 | Meningioma | 13 | 2.86E+06 |
| ABT SMH-73 | Meningioma | 15 | 3.85E+06 |
| ABT SMH-73 | Meningioma | 9 | 2.26E+06 |
| ABT SMH-73 | Meningioma | 11 | 4.71E+06 |
| ABT SMH-73 | Meningioma | 13 | 3.17E+06 |
| ABT SMH-73 | Meningioma | 12 | 5.10E+06 |
| ABT SMH-73 | Meningioma | 12 | 2.56E+06 |
| ABT SMH-73 | Meningioma | 12 | 3.46E+06 |
| ABT SMH-73 | Meningioma | 12 | 4.25E+06 |
| ABT SMH-73 | Meningioma | 11 | 2.68E+06 |
| ABT SMH-19 | Metastatic | 14 | 1.02E+06 |
| ABT SMH-19 | Metastatic | 7 | 6.75E+05 |
| ABT SMH-19 | Metastatic | 13 | 1.72E+06 |
| ABT SMH-19 | Metastatic | 10 | 1.75E+06 |
| ABT SMH-19 | Metastatic | 12 | 5.43E+05 |
| ABT SMH-19 | Metastatic | 12 | 4.24E+05 |
| ABT SMH-19 | Metastatic | 12 | 1.15E+06 |
| ABT SMH-19 | Metastatic | 11 | 1.63E+06 |
| ABT SMH-19 | Metastatic | 13 | 1.25E+06 |
| ABT SMH-19 | Metastatic | 12 | 2.96E+06 |
| ABT SMH-19 | Metastatic | 14 | 1.70E+06 |
| ABT SMH-195 | Metastatic | 15 | 4.84E+06 |
| ABT SMH-195 | Metastatic | 13 | 4.02E+06 |
| ABT SMH-195 | Metastatic | 15 | 3.10E+06 |
| ABT SMH-195 | Metastatic | 13 | 1.29E+06 |
| ABT SMH-195 | Metastatic | 15 | 1.61E+06 |
| ABT SMH-195 | Metastatic | 16 | 9.91E+05 |
| ABT SMH-195 | Metastatic | 13 | 8.70E+05 |

| | | | |
|---|---|---|---|
| ABT SMH-195 | Metastatic | 13 | 1.79E+06 |
| ABT SMH-195 | Metastatic | 13 | 1.69E+06 |
| ABT SMH-195 | Metastatic | 15 | 7.27E+05 |
| ABT SMH-195 | Metastatic | 13 | 5.43E+05 |
| ABT SMH-195 | Metastatic | 18 | 5.94E+05 |
| ABT SMH-195 | Metastatic | 15 | 1.81E+06 |
| ABT SMH-197 | Metastatic | 13 | 1.98E+06 |
| ABT SMH-197 | Metastatic | 13 | 2.35E+06 |
| ABT SMH-197 | Metastatic | 14 | 1.59E+06 |
| ABT SMH-197 | Metastatic | 13 | 2.15E+06 |
| ABT SMH-197 | Metastatic | 13 | 1.93E+06 |
| ABT SMH-197 | Metastatic | 16 | 1.77E+06 |
| ABT SMH-197 | Metastatic | 13 | 1.22E+06 |
| ABT SMH-197 | Metastatic | 12 | 9.09E+05 |
| ABT SMH-197 | Metastatic | 12 | 1.28E+06 |
| ABT SMH-197 | Metastatic | 17 | 9.54E+05 |
| ABT SMH-197 | Metastatic | 12 | 3.19E+05 |
| ABT SMH-197 | Metastatic | 9 | 8.60E+05 |
| ABT SMH-205 | Metastatic | 13 | 1.02E+06 |
| ABT SMH-205 | Metastatic | 15 | 2.40E+06 |
| ABT SMH-205 | Metastatic | 14 | 1.80E+06 |
| ABT SMH-205 | Metastatic | 15 | 2.26E+06 |
| ABT SMH-205 | Metastatic | 10 | 8.60E+05 |
| ABT SMH-205 | Metastatic | 12 | 3.16E+05 |
| ABT SMH-205 | Metastatic | 11 | 7.85E+05 |
| ABT SMH-205 | Metastatic | 13 | 8.58E+05 |
| ABT SMH-205 | Metastatic | 14 | 1.06E+06 |
| ABT SMH-205 | Metastatic | 16 | 8.06E+05 |
| ABT SMH-207 | Metastatic | 7 | 1.45E+06 |
| ABT SMH-207 | Metastatic | 11 | 4.16E+06 |
| ABT SMH-207 | Metastatic | 12 | 5.21E+06 |
| ABT SMH-207 | Metastatic | 12 | 5.49E+06 |
| ABT SMH-207 | Metastatic | 11 | 4.84E+06 |
| ABT SMH-207 | Metastatic | 12 | 3.49E+06 |
| ABT SMH-207 | Metastatic | 12 | 2.09E+06 |
| ABT SMH-207 | Metastatic | 14 | 2.00E+06 |
| ABT SMH-211 | Metastatic | 13 | 3.55E+06 |
| ABT SMH-211 | Metastatic | 10 | 1.70E+06 |
| ABT SMH-211 | Metastatic | 9 | 1.79E+06 |
| ABT SMH-211 | Metastatic | 11 | 2.63E+06 |
| ABT SMH-211 | Metastatic | 9 | 7.95E+05 |
| ABT SMH-211 | Metastatic | 11 | 1.50E+06 |
| ABT SMH-211 | Metastatic | 12 | 3.80E+06 |
| ABT SMH-211 | Metastatic | 11 | 1.98E+06 |
| ABT SMH-211 | Metastatic | 12 | 1.81E+06 |
| ABT SMH-211 | Metastatic | 12 | 1.33E+06 |
| ABT SMH-212 | Metastatic | 15 | 4.65E+06 |
| ABT SMH-212 | Metastatic | 12 | 3.17E+06 |
| ABT SMH-212 | Metastatic | 15 | 5.51E+06 |
| ABT SMH-212 | Metastatic | 9 | 3.85E+06 |
| ABT SMH-212 | Metastatic | 12 | 4.20E+06 |
| ABT SMH-212 | Metastatic | 16 | 5.76E+06 |
| ABT SMH-212 | Metastatic | 13 | 5.23E+06 |
| ABT SMH-212 | Metastatic | 10 | 2.27E+06 |
| ABT SMH-212 | Metastatic | 15 | 1.00E+07 |
| ABT SMH-212 | Metastatic | 13 | 6.71E+06 |
| ABT SMH-212 | Metastatic | 12 | 3.38E+06 |
| ABT SMH-212 | Metastatic | 17 | 3.56E+06 |
| ABT SMH-218 | Metastatic | 14 | 3.19E+06 |
| ABT SMH-218 | Metastatic | 12 | 2.57E+06 |
| ABT SMH-218 | Metastatic | 12 | 3.84E+06 |
| ABT SMH-218 | Metastatic | 13 | 1.92E+06 |
| ABT SMH-218 | Metastatic | 13 | 7.93E+06 |
| ABT SMH-218 | Metastatic | 12 | 1.93E+06 |
| ABT SMH-218 | Metastatic | 11 | 6.00E+06 |
| ABT SMH-220 | Metastatic | 14 | 5.85E+06 |
| ABT SMH-220 | Metastatic | 11 | 4.19E+06 |
| ABT SMH-220 | Metastatic | 12 | 3.56E+06 |
| ABT SMH-220 | Metastatic | 12 | 4.74E+06 |
| ABT SMH-220 | Metastatic | 13 | 3.75E+06 |
| ABT SMH-220 | Metastatic | 12 | 2.96E+06 |
| ABT SMH-220 | Metastatic | 10 | 9.86E+05 |
| ABT SMH-220 | Metastatic | 12 | 1.21E+06 |
| ABT SMH-220 | Metastatic | 13 | 5.40E+06 |
| ABT SMH-220 | Metastatic | 12 | 3.73E+06 |
| ABT SMH-220 | Metastatic | 10 | 2.90E+06 |
| ABT SMH-221 | Metastatic | 14 | 3.58E+06 |
| ABT SMH-221 | Metastatic | 12 | 5.07E+06 |
| ABT SMH-221 | Metastatic | 12 | 6.86E+06 |
| ABT SMH-221 | Metastatic | 12 | 7.31E+06 |
| ABT SMH-221 | Metastatic | 12 | 1.02E+07 |
| ABT SMH-221 | Metastatic | 12 | 9.58E+06 |
| ABT SMH-221 | Metastatic | 12 | 1.07E+07 |
| ABT SMH-222 | Metastatic | 13 | 6.41E+06 |
| ABT SMH-222 | Metastatic | 13 | 6.47E+06 |
| ABT SMH-222 | Metastatic | 9 | 3.00E+06 |
| ABT SMH-222 | Metastatic | 10 | 3.66E+06 |
| ABT SMH-222 | Metastatic | 11 | 4.89E+06 |
| ABT SMH-222 | Metastatic | 12 | 5.63E+06 |
| ABT SMH-222 | Metastatic | 11 | 4.04E+06 |
| ABT SMH-222 | Metastatic | 12 | 5.83E+06 |
| ABT SMH-229 | Metastatic | 11 | 5.52E+06 |
| ABT SMH-229 | Metastatic | 13 | 2.74E+06 |
| ABT SMH-229 | Metastatic | 12 | 3.01E+06 |
| ABT SMH-229 | Metastatic | 10 | 6.65E+06 |
| ABT SMH-229 | Metastatic | 13 | 4.84E+06 |
| ABT SMH-229 | Metastatic | 11 | 6.71E+06 |
| ABT SMH-229 | Metastatic | 12 | 3.57E+06 |
| ABT SMH-229 | Metastatic | 16 | 6.53E+06 |
| ABT SMH-229 | Metastatic | 11 | 3.74E+06 |

| | | | |
|---|---|---|---|
| ABT SMH-229 | Metastatic | 14 | 9.88E+06 |
| ABT SMH-23 | Metastatic | 12 | 1.34E+06 |
| ABT SMH-23 | Metastatic | 13 | 2.45E+06 |
| ABT SMH-23 | Metastatic | 10 | 1.64E+06 |
| ABT SMH-23 | Metastatic | 12 | 1.49E+06 |
| ABT SMH-23 | Metastatic | 13 | 1.69E+06 |
| ABT SMH-23 | Metastatic | 12 | 1.92E+06 |
| ABT SMH-23 | Metastatic | 13 | 1.51E+06 |
| ABT SMH-23 | Metastatic | 12 | 1.70E+06 |
| ABT SMH-23 | Metastatic | 12 | 1.35E+06 |
| ABT SMH-23 | Metastatic | 12 | 7.66E+05 |
| ABT SMH-23 | Metastatic | 13 | 8.79E+05 |
| ABT SMH-230 | Metastatic | 13 | 7.62E+06 |
| ABT SMH-230 | Metastatic | 12 | 3.72E+06 |
| ABT SMH-230 | Metastatic | 12 | 4.07E+06 |
| ABT SMH-230 | Metastatic | 13 | 2.68E+06 |
| ABT SMH-230 | Metastatic | 11 | 6.25E+06 |
| ABT SMH-230 | Metastatic | 13 | 8.07E+06 |
| ABT SMH-230 | Metastatic | 13 | 4.51E+06 |
| ABT SMH-230 | Metastatic | 10 | 3.86E+06 |
| ABT SMH-230 | Metastatic | 11 | 1.71E+06 |
| ABT SMH-230 | Metastatic | 12 | 4.42E+06 |
| ABT SMH-232 | Metastatic | 14 | 3.76E+06 |
| ABT SMH-232 | Metastatic | 13 | 2.66E+06 |
| ABT SMH-232 | Metastatic | 13 | 3.17E+06 |
| ABT SMH-232 | Metastatic | 13 | 3.47E+06 |
| ABT SMH-232 | Metastatic | 11 | 2.41E+06 |
| ABT SMH-232 | Metastatic | 12 | 3.10E+06 |
| ABT SMH-232 | Metastatic | 13 | 2.50E+06 |
| ABT SMH-232 | Metastatic | 13 | 3.85E+06 |
| ABT SMH-232 | Metastatic | 12 | 3.22E+06 |
| ABT SMH-232 | Metastatic | 12 | 1.37E+06 |
| ABT SMH-235 | Metastatic | 13 | 7.63E+06 |
| ABT SMH-235 | Metastatic | 12 | 6.41E+06 |
| ABT SMH-235 | Metastatic | 12 | 5.88E+06 |
| ABT SMH-235 | Metastatic | 12 | 4.99E+06 |
| ABT SMH-235 | Metastatic | 7 | 1.55E+06 |
| ABT SMH-235 | Metastatic | 10 | 3.17E+06 |
| ABT SMH-235 | Metastatic | 9 | 2.67E+06 |
| ABT SMH-235 | Metastatic | 11 | 3.68E+06 |
| ABT SMH-235 | Metastatic | 11 | 2.40E+06 |
| ABT SMH-239 | Metastatic | 12 | 6.26E+06 |
| ABT SMH-239 | Metastatic | 11 | 2.48E+06 |
| ABT SMH-239 | Metastatic | 13 | 4.87E+06 |
| ABT SMH-239 | Metastatic | 11 | 3.55E+06 |
| ABT SMH-239 | Metastatic | 13 | 2.50E+06 |
| ABT SMH-239 | Metastatic | 12 | 1.91E+06 |
| ABT SMH-239 | Metastatic | 10 | 1.63E+06 |
| ABT SMH-239 | Metastatic | 14 | 3.91E+06 |
| ABT SMH-239 | Metastatic | 9 | 8.87E+05 |
| ABT SMH-25 | Metastatic | 13 | 6.15E+06 |
| ABT SMH-25 | Metastatic | 13 | 6.29E+06 |
| ABT SMH-25 | Metastatic | 13 | 3.98E+06 |
| ABT SMH-25 | Metastatic | 12 | 5.70E+06 |
| ABT SMH-25 | Metastatic | 15 | 3.18E+06 |
| ABT SMH-25 | Metastatic | 16 | 3.42E+06 |
| ABT SMH-25 | Metastatic | 12 | 3.98E+06 |
| ABT SMH-25 | Metastatic | 13 | 4.42E+06 |
| ABT SMH-25 | Metastatic | 16 | 5.16E+06 |
| ABT SMH-25 | Metastatic | 13 | 1.49E+06 |
| ABT SMH-25 | Metastatic | 11 | 4.52E+06 |
| ABT SMH-253 | Metastatic | 15 | 2.69E+06 |
| ABT SMH-253 | Metastatic | 14 | 1.24E+06 |
| ABT SMH-253 | Metastatic | 10 | 1.53E+06 |
| ABT SMH-253 | Metastatic | 15 | 3.60E+06 |
| ABT SMH-255 | Metastatic | 13 | 9.70E+05 |
| ABT SMH-255 | Metastatic | 14 | 1.97E+06 |
| ABT SMH-255 | Metastatic | 12 | 2.60E+06 |
| ABT SMH-255 | Metastatic | 14 | 1.76E+06 |
| ABT SMH-255 | Metastatic | 14 | 1.49E+06 |
| ABT SMH-257 | Metastatic | 15 | 3.25E+06 |
| ABT SMH-257 | Metastatic | 9 | 2.94E+06 |
| ABT SMH-257 | Metastatic | 11 | 4.40E+06 |
| ABT SMH-257 | Metastatic | 13 | 1.56E+06 |
| ABT SMH-257 | Metastatic | 12 | 5.73E+06 |
| ABT SMH-257 | Metastatic | 12 | 2.84E+06 |
| ABT SMH-257 | Metastatic | 11 | 2.32E+06 |
| ABT SMH-257 | Metastatic | 12 | 6.09E+06 |
| ABT SMH-257 | Metastatic | 14 | 4.02E+06 |
| ABT SMH-257 | Metastatic | 11 | 1.87E+06 |
| ABT SMH-263 | Metastatic | 14 | 6.99E+06 |
| ABT SMH-263 | Metastatic | 13 | 3.01E+06 |
| ABT SMH-263 | Metastatic | 12 | 2.28E+06 |
| ABT SMH-263 | Metastatic | 12 | 1.63E+06 |
| ABT SMH-263 | Metastatic | 11 | 3.17E+06 |
| ABT SMH-263 | Metastatic | 18 | 6.65E+06 |
| ABT SMH-263 | Metastatic | 13 | 4.10E+06 |
| ABT SMH-263 | Metastatic | 12 | 6.76E+06 |
| ABT SMH-263 | Metastatic | 13 | 4.06E+06 |
| ABT SMH-266 | Metastatic | 15 | 4.89E+06 |
| ABT SMH-266 | Metastatic | 11 | 3.12E+06 |
| ABT SMH-266 | Metastatic | 11 | 2.54E+06 |
| ABT SMH-266 | Metastatic | 11 | 4.09E+06 |
| ABT SMH-266 | Metastatic | 13 | 2.56E+06 |
| ABT SMH-266 | Metastatic | 12 | 3.41E+06 |
| ABT SMH-266 | Metastatic | 12 | 4.59E+06 |
| ABT SMH-266 | Metastatic | 10 | 4.80E+06 |
| ABT SMH-266 | Metastatic | 12 | 2.84E+06 |
| ABT SMH-266 | Metastatic | 14 | 1.36E+06 |
| ABT SMH-266 | Metastatic | 10 | 2.16E+06 |

| | | | |
|---|---|---|---|
| ABT SMH-266 | Metastatic | 13 | 5.33E+06 |
| ABT SMH-268 | Metastatic | 14 | 3.89E+06 |
| ABT SMH-268 | Metastatic | 12 | 4.31E+06 |
| ABT SMH-268 | Metastatic | 11 | 1.95E+06 |
| ABT SMH-268 | Metastatic | 11 | 2.05E+06 |
| ABT SMH-268 | Metastatic | 12 | 2.72E+06 |
| ABT SMH-268 | Metastatic | 12 | 2.55E+06 |
| ABT SMH-268 | Metastatic | 10 | 2.49E+06 |
| ABT SMH-268 | Metastatic | 12 | 2.47E+06 |
| ABT SMH-268 | Metastatic | 10 | 1.65E+06 |
| ABT SMH-270 | Metastatic | 12 | 2.24E+06 |
| ABT SMH-270 | Metastatic | 13 | 1.84E+06 |
| ABT SMH-270 | Metastatic | 13 | 1.69E+06 |
| ABT SMH-270 | Metastatic | 12 | 2.72E+06 |
| ABT SMH-270 | Metastatic | 12 | 2.55E+06 |
| ABT SMH-270 | Metastatic | 14 | 2.03E+06 |
| ABT SMH-270 | Metastatic | 13 | 1.85E+06 |
| ABT SMH-270 | Metastatic | 14 | 2.93E+06 |
| ABT SMH-270 | Metastatic | 16 | 2.19E+06 |
| ABT SMH-270 | Metastatic | 13 | 1.61E+06 |
| ABT SMH-270 | Metastatic | 10 | 1.17E+06 |
| ABT SMH-270 | Metastatic | 13 | 1.86E+06 |
| ABT SMH-275 | Metastatic | 14 | 6.20E+06 |
| ABT SMH-275 | Metastatic | 12 | 5.01E+06 |
| ABT SMH-275 | Metastatic | 12 | 5.78E+06 |
| ABT SMH-275 | Metastatic | 12 | 5.79E+06 |
| ABT SMH-275 | Metastatic | 13 | 5.15E+06 |
| ABT SMH-277 | Metastatic | 13 | 6.55E+06 |
| ABT SMH-277 | Metastatic | 13 | 8.53E+06 |
| ABT SMH-277 | Metastatic | 13 | 9.15E+06 |
| ABT SMH-277 | Metastatic | 12 | 5.45E+06 |
| ABT SMH-277 | Metastatic | 13 | 6.29E+06 |
| ABT SMH-277 | Metastatic | 12 | 6.02E+06 |
| ABT SMH-277 | Metastatic | 13 | 5.10E+06 |
| ABT SMH-277 | Metastatic | 13 | 7.41E+06 |
| ABT SMH-277 | Metastatic | 12 | 8.28E+06 |
| ABT SMH-277 | Metastatic | 11 | 6.57E+06 |
| ABT SMH-28 | Metastatic | 13 | 4.89E+05 |
| ABT SMH-28 | Metastatic | 16 | 4.78E+05 |
| ABT SMH-28 | Metastatic | 12 | 1.27E+06 |
| ABT SMH-28 | Metastatic | 6 | 3.27E+05 |
| ABT SMH-28 | Metastatic | 15 | 8.48E+05 |
| ABT SMH-28 | Metastatic | 14 | 1.08E+06 |
| ABT SMH-28 | Metastatic | 13 | 9.02E+05 |
| ABT SMH-28 | Metastatic | 13 | 1.51E+06 |
| ABT SMH-28 | Metastatic | 13 | 1.72E+06 |
| ABT SMH-375 | Metastatic | 13 | 1.35E+06 |
| ABT SMH-375 | Metastatic | 11 | 1.75E+06 |
| ABT SMH-375 | Metastatic | 12 | 1.10E+06 |
| ABT SMH-375 | Metastatic | 13 | 1.88E+06 |
| ABT SMH-375 | Metastatic | 10 | 2.08E+06 |
| ABT SMH-375 | Metastatic | 14 | 1.94E+06 |
| ABT SMH-375 | Metastatic | 11 | 1.46E+06 |
| ABT SMH-375 | Metastatic | 15 | 9.89E+05 |
| ABT SMH-375 | Metastatic | 12 | 1.01E+06 |
| ABT SMH-380 | Metastatic | 10 | 2.03E+06 |
| ABT SMH-380 | Metastatic | 12 | 1.35E+06 |
| ABT SMH-380 | Metastatic | 13 | 2.45E+06 |
| ABT SMH-380 | Metastatic | 15 | 1.24E+06 |
| ABT SMH-380 | Metastatic | 13 | 1.09E+06 |
| ABT SMH-380 | Metastatic | 12 | 2.32E+06 |
| ABT SMH-380 | Metastatic | 16 | 2.68E+06 |
| ABT SMH-385 | Metastatic | 12 | 2.72E+06 |
| ABT SMH-385 | Metastatic | 11 | 1.87E+06 |
| ABT SMH-385 | Metastatic | 8 | 4.57E+05 |
| ABT SMH-385 | Metastatic | 10 | 1.09E+06 |
| ABT SMH-387 | Metastatic | 11 | 1.49E+06 |
| ABT SMH-387 | Metastatic | 13 | 2.72E+06 |
| ABT SMH-387 | Metastatic | 11 | 2.19E+06 |
| ABT SMH-387 | Metastatic | 12 | 2.93E+06 |
| ABT SMH-387 | Metastatic | 10 | 1.14E+06 |
| ABT SMH-387 | Metastatic | 14 | 3.40E+06 |
| ABT SMH-387 | Metastatic | 12 | 3.85E+06 |
| ABT SMH-387 | Metastatic | 8 | 2.69E+06 |
| ABT SMH-387 | Metastatic | 14 | 1.05E+06 |
| ABT SMH-400 | Metastatic | 13 | 2.23E+06 |
| ABT SMH-400 | Metastatic | 14 | 4.44E+06 |
| ABT SMH-400 | Metastatic | 14 | 3.22E+06 |
| ABT SMH-400 | Metastatic | 12 | 2.24E+06 |
| ABT SMH-400 | Metastatic | 11 | 2.44E+06 |
| ABT SMH-400 | Metastatic | 14 | 1.23E+06 |
| ABT SMH-400 | Metastatic | 13 | 2.66E+06 |
| ABT SMH-400 | Metastatic | 13 | 4.72E+06 |
| ABT SMH-402 | Metastatic | 11 | 3.68E+06 |
| ABT SMH-402 | Metastatic | 13 | 5.33E+06 |
| ABT SMH-402 | Metastatic | 8 | 1.83E+06 |
| ABT SMH-402 | Metastatic | 10 | 2.80E+06 |
| ABT SMH-402 | Metastatic | 12 | 3.44E+06 |
| ABT SMH-402 | Metastatic | 10 | 9.98E+05 |
| ABT SMH-402 | Metastatic | 12 | 3.29E+06 |
| ABT SMH-402 | Metastatic | 11 | 1.91E+06 |
| ABT SMH-404 | Metastatic | 12 | 2.83E+06 |
| ABT SMH-404 | Metastatic | 12 | 2.72E+06 |
| ABT SMH-404 | Metastatic | 10 | 7.12E+05 |
| ABT SMH-404 | Metastatic | 12 | 2.60E+06 |
| ABT SMH-404 | Metastatic | 13 | 2.62E+06 |
| ABT SMH-404 | Metastatic | 12 | 3.41E+06 |
| ABT SMH-404 | Metastatic | 13 | 2.94E+06 |
| ABT SMH-404 | Metastatic | 10 | 7.89E+05 |
| ABT SMH-404 | Metastatic | 16 | 4.18E+06 |

| | | | |
|---|---|---|---|
| ABT SMH-404 | Metastatic | 14 | 4.53E+06 |
| ABT SMH-404 | Metastatic | 13 | 4.69E+06 |
| ABT SMH-407 | Metastatic | 11 | 8.17E+05 |
| ABT SMH-407 | Metastatic | 13 | 1.45E+06 |
| ABT SMH-407 | Metastatic | 14 | 1.85E+06 |
| ABT SMH-407 | Metastatic | 11 | 2.16E+06 |
| ABT SMH-407 | Metastatic | 11 | 3.51E+06 |
| ABT SMH-407 | Metastatic | 10 | 3.67E+06 |
| ABT SMH-407 | Metastatic | 13 | 3.92E+06 |
| ABT SMH-407 | Metastatic | 13 | 5.61E+06 |
| ABT SMH-407 | Metastatic | 12 | 4.77E+06 |
| ABT SMH-407 | Metastatic | 13 | 2.04E+06 |
| ABT SMH-408 | Metastatic | 13 | 6.90E+06 |
| ABT SMH-408 | Metastatic | 11 | 3.40E+06 |
| ABT SMH-408 | Metastatic | 13 | 6.05E+06 |
| ABT SMH-408 | Metastatic | 13 | 1.92E+06 |
| ABT SMH-408 | Metastatic | 11 | 1.57E+06 |
| ABT SMH-408 | Metastatic | 9 | 3.22E+06 |
| ABT SMH-408 | Metastatic | 13 | 2.25E+06 |
| ABT SMH-408 | Metastatic | 13 | 3.77E+06 |
| ABT SMH-408 | Metastatic | 11 | 2.42E+06 |
| ABT SMH-408 | Metastatic | 11 | 4.26E+06 |
| ABT SMH-408 | Metastatic | 16 | 3.68E+06 |
| ABT SMH-408 | Metastatic | 13 | 4.01E+06 |
| ABT SMH-477 | Metastatic | 13 | 2.55E+06 |
| ABT SMH-477 | Metastatic | 13 | 2.03E+06 |
| ABT SMH-477 | Metastatic | 13 | 2.13E+06 |
| ABT SMH-477 | Metastatic | 13 | 4.65E+06 |
| ABT SMH-477 | Metastatic | 12 | 2.35E+06 |
| ABT SMH-477 | Metastatic | 16 | 1.38E+06 |
| ABT SMH-477 | Metastatic | 12 | 8.95E+05 |
| ABT SMH-77 | Metastatic | 13 | 1.86E+06 |
| ABT SMH-77 | Metastatic | 10 | 9.47E+05 |
| ABT SMH-77 | Metastatic | 11 | 1.43E+06 |
| ABT SMH-77 | Metastatic | 13 | 2.09E+06 |
| ABT SMH-77 | Metastatic | 14 | 2.56E+06 |
| ABT SMH-77 | Metastatic | 14 | 1.23E+06 |
| ABT SMH-78 | Metastatic | 13 | 1.48E+06 |
| ABT SMH-78 | Metastatic | 13 | 1.12E+06 |
| ABT SMH-78 | Metastatic | 15 | 1.45E+06 |
| ABT SMH-78 | Metastatic | 14 | 2.75E+06 |
| ABT SMH-78 | Metastatic | 14 | 2.51E+06 |
| ABT SMH-78 | Metastatic | 13 | 1.53E+06 |
| ABT SMH-78 | Metastatic | 15 | 8.22E+05 |
| ABT SMH-78 | Metastatic | 16 | 2.34E+06 |
| ABT SMH-81 | Metastatic | 12 | 1.20E+06 |
| ABT SMH-81 | Metastatic | 11 | 2.56E+06 |
| ABT SMH-81 | Metastatic | 13 | 4.40E+06 |
| ABT SMH-81 | Metastatic | 13 | 3.64E+06 |
| ABT SMH-81 | Metastatic | 10 | 2.00E+06 |
| ABT SMH-81 | Metastatic | 13 | 2.43E+06 |
| ABT SMH-81 | Metastatic | 11 | 1.23E+06 |
| ABT SMH-83 | Metastatic | 13 | 2.32E+06 |
| ABT SMH-83 | Metastatic | 12 | 1.51E+06 |
| ABT SMH-83 | Metastatic | 11 | 2.17E+06 |
| ABT SMH-83 | Metastatic | 13 | 1.85E+06 |
| ABT SMH-83 | Metastatic | 12 | 2.53E+06 |
| ABT SMH-83 | Metastatic | 12 | 1.17E+06 |
| ABT SMH-83 | Metastatic | 12 | 2.23E+06 |
| ABT SMH-83 | Metastatic | 11 | 1.94E+06 |

**Table S1. The signal duration and signal intensity of specimens used in Fig. 2 PCA-LDA models.** Here, n=122 specimens with breakdowns listed across meningioma, schwannoma and metastatic cancers producing n=959, 10-second PIRL-MS sampling events possessed signal duration and signal intensities of 13±2s and $(3\pm2)\cdot10^6$, respectively (± 1 standard deviation).

**20% leave out**

| | Data point groups | | | | | Correct Classification Rate | |
|---|---|---|---|---|---|---|---|
| | PIRL-MS spectra data points | Correctly classified data points | Misclassified data points | Unclassifiable data points | Classifiable data points | Per classifiable data points | Per all (classifiable and unclassifiable) data points |
| **Total** | **959** | **938** | **11** | **10** | **949** | **98.84%** | **97.81%** |

| | Meningioma | Metastatic | Schwannoma | Unclassifiable | **Total** |
|---|---|---|---|---|---|
| Meningioma | 312 | 1 | 4 | 5 | **322** |
| Metastatic | 0 | 374 | 0 | 4 | **378** |
| Schwannoma | 6 | 0 | 252 | 1 | **259** |
| **Total** | **318** | **375** | **256** | **10** | **959** |

**Full group leave out**

| | Data point groups | | | | | Correct Classification Rate | |
|---|---|---|---|---|---|---|---|
| Group | PIRL-MS spectra data points | Correctly classified data points | Misclassified data points | Unclassifiable data points | Classifiable data points | Per classifiable data points | Per all (classifiable and unclassifiable) data points |
| 1 | 209 | 196 | 2 | 11 | 198 | 98.99% | 93.78% |
| 2 | 200 | 180 | 2 | 18 | 182 | 98.90% | 90.00% |
| 3 | 199 | 166 | 17 | 16 | 183 | 90.71% | 83.42% |
| 4 | 209 | 186 | 6 | 17 | 192 | 96.88% | 89.00% |
| 5 | 142 | 124 | 6 | 12 | 130 | 95.38% | 87.32% |
| **Total** | **959** | **852** | **33** | **74** | **885** | **96.27%** | **88.84%** |

| | Meningioma | Metastatic | Schwannoma | Unclassifiable | **Total** |
|---|---|---|---|---|---|
| Meningioma | 291 | 0 | 9 | 22 | **322** |
| Metastatic | 0 | 330 | 9 | 39 | **378** |
| Schwannoma | 10 | 5 | 231 | 13 | **259** |
| **Total** | **301** | **335** | **249** | **74** | **959** |

**Table S2. Cross-validation statistics of Fig. 2A PCA-LDA model.** Here we preformed cross-validation (using 20% leave-out tests) for the multivariate model of major spinal tumour types shown in Fig. 2A. The cross-validation results across both 20% leave-out and full group leave-out is listed alongside the confusion matrix for each test. As can be seen, strong cross-validation accuracies were obtained across both tests indicative of model robustness and lack of significant intraspecimen heterogeneities that would have otherwise resulted in a discordance between the two (i.e., 20% and full group leave-out) cross-validation results. As mentioned in the text, the 20% leave out test allows independent measurements from the surface of each independent specimen to populate both the model and test sets, and this does not happen in a full group leave-out wherein the sampling events from a particular specimen is only classified against measurements made from other independent specimens. Concordant cross-validation results seen (98.84% and 96.27%) stem from a generalizable model that captures both intra- and interspecimen heterogeneity appropriately. Analysis of the confusion matrices suggested that no class was exhibiting a drastic preferential misdiagnosis compared to others. The model's challenges in the accurate classification thus applied to all three tumour types with the model, however, exhibiting slightly poorer performance in identifying schwannomas and was most rigorous in identifying metastatic cancers.

**20% leave out**

| | Data point groups | | | | | | Correct Classification Rate | |
|---|---|---|---|---|---|---|---|---|
| | PIRL-MS spectra data points | Correctly classified data points | Misclassified data points | Unclassifiable data points | Classifiable data points | | Per classifiable data points | Per all (classifiable and unclassifiable) data points |
| **Total** | 122 | 50 | 71 | 1 | **121** | | **41.32%** | **40.98%** |

| | 1 | 2 | 3 | Unclassifiable | Total |
|---|---|---|---|---|---|
| Permutated 1 | 16 | 11 | 13 | 0 | 40 |
| Permutated 2 | 11 | 13 | 16 | 1 | 41 |
| Permutated 3 | 9 | 11 | 21 | 0 | 41 |
| **Total** | **36** | **35** | **50** | **1** | **122** |

**Full group leave out**

| Group | PIRL-MS spectra data points | Correctly classified data points | Misclassified data points | Unclassifiable data points | Classifiable data points | Per classifiable data points | Per all (classifiable and unclassifiable) data points |
|---|---|---|---|---|---|---|---|
| 1 | 26 | 13 | 13 | 0 | 26 | 50.00% | 50.00% |
| 2 | 25 | 9 | 16 | 0 | 25 | 36.00% | 36.00% |
| 3 | 27 | 12 | 15 | 0 | 27 | 44.44% | 44.44% |
| 4 | 25 | 6 | 19 | 0 | 25 | 24.00% | 24.00% |
| 5 | 19 | 3 | 15 | 1 | 18 | 16.67% | 15.79% |
| **Total** | **122** | **43** | **78** | **1** | **121** | **35.54%** | **35.25%** |

| | 1 | 2 | 3 | Unclassifiable | **Total** |
|---|---|---|---|---|---|
| Permutated 1 | 15 | 13 | 12 | 0 | **40** |
| Permutated 2 | 11 | 13 | 16 | 1 | **41** |
| Permutated 3 | 15 | 11 | 15 | 0 | **41** |
| **Total** | **41** | **37** | **43** | **1** | **122** |

**Table S3. Cross-validation statistics of Fig. 2B PCA-LDA model.** This table summarizes the cross-validation test results of the permutated (false annotation, mixed class groups) model across the same two types of cross-validation tests of 20% leave-out and full group leave-out described in the legend accompanying Table S2. Consistent with the PCA-LDA model presented in Fig. 2B, the low cross-validation accuracies across both cross-validation methods of 41.32% and 35.54% in this table (compared to 98.84% and 96.27% for true annotation model of Fig.2A/Table S2) suggest that the permutated model essentially possesses indistinguishable groups. This further validates the class separations seen in Fig. 2A for true annotations as being significant, unlikely influenced by overfitting. The permutated model used the same parameters as the true annotations including number of PCA components (as n=194) and standard deviation for cluster overlap assessment from (standard deviation of n=4), all evaluated using a 5-fold (20%) cross-validation test.

| | | | | Fig. 2A model - Full mass range | | | Fig. 2A model - Sparse Analysis | | | | |
|---|---|---|---|---|---|---|---|---|---|---|---|
| Specimen ID | True Class | Duration of Signal | Total Ion Count (TIC) | PCA-LDA Prediction (AMX Recognition) | Probability | % Spatially Invariant Correct Sampling Event | PCA-LDA Prediction (AMX Recognition) | Probability | % Spatially Invariant Correct Sampling Event | Sum of spectral coefficient of correlation (at 1 Da bins) | Sox10 IHC status |
| **Unknown 1** | Meningioma | 11 | 4.81E+06 | Meningioma | 73.73 | 100.00 | Meningioma | 95.96 | 100.00 | 3.00E+05 | |
| **Unknown 1** | Meningioma | 11 | 6.61E+06 | Meningioma | 98.77 | | Meningioma | 94.61 | | | |
| **Unknown 1** | Meningioma | 12 | 8.26E+06 | Meningioma | 96.17 | | Meningioma | 98.4 | | | |
| **Unknown 1** | Meningioma | 11 | 6.44E+06 | Meningioma | 95.11 | | Meningioma | 98.61 | | | |
| **Unknown 1** | Meningioma | 8 | 4.19E+06 | Meningioma | 99.37 | | Meningioma | 99.36 | | | |
| **Unknown 1** | Meningioma | 11 | 7.26E+06 | Meningioma | 99.36 | | Meningioma | 99.38 | | | |
| **Unknown 1** | Meningioma | 12 | 1.23E+07 | Meningioma | 97.29 | | Meningioma | 96.26 | | | |
| **Unknown 2** | Metastatic | 1 | 1.60E+05 | Bad Data | - | 100.00 | Bad Data | - | 100.00 | 2.91E+05 | |
| **Unknown 2** | Metastatic | 13 | 1.01E+06 | Metastatic | 99.33 | | Metastatic | 99.22 | | | |
| **Unknown 2** | Metastatic | 7 | 2.59E+06 | Metastatic | 98.89 | | Metastatic | 98.87 | | | |
| **Unknown 2** | Metastatic | 11 | 2.48E+06 | Metastatic | 98.6 | | Metastatic | 96.54 | | | |
| **Unknown 2** | Metastatic | 13 | 9.21E+05 | Metastatic | 99.33 | | Metastatic | 88.81 | | | |
| **Unknown 2** | Metastatic | 10 | 2.19E+06 | Metastatic | 99.31 | | Metastatic | 99.31 | | | |
| **Unknown 2** | Metastatic | 10 | 1.08E+06 | Metastatic | 98.93 | | Metastatic | 93.62 | | | |
| **Unknown 2** | Metastatic | 5 | 7.44E+05 | Metastatic | 99.33 | | Metastatic | 93.72 | | | |
| **Unknown 2** | Metastatic | 9 | 2.54E+06 | Metastatic | 99.24 | | Metastatic | 99.22 | | | |
| **Unknown 2** | Metastatic | 11 | 2.18E+06 | Metastatic | 99.2 | | Metastatic | 98.02 | | | |
| **Unknown 3** | Meningioma | 11 | 6.23E+06 | Meningioma | 99.26 | 100.00 | Meningioma | 99.28 | 100.00 | 4.54E+05 | |
| **Unknown 3** | Meningioma | 1 | 1.39E+05 | Bad Data | - | | Bad Data | - | | | |
| **Unknown 3** | Meningioma | 9 | 4.57E+06 | Meningioma | 99.35 | | Meningioma | 99.28 | | | |
| **Unknown 3** | Meningioma | 11 | 6.70E+06 | Meningioma | 99.15 | | Meningioma | 99.36 | | | |
| **Unknown 3** | Meningioma | 11 | 5.16E+06 | Meningioma | 99.32 | | Meningioma | 99.38 | | | |
| **Unknown 3** | Meningioma | 10 | 3.03E+06 | Meningioma | 99.31 | | Meningioma | 99.26 | | | |
| **Unknown 4** | Meningioma | 12 | 2.60E+06 | Meningioma | 99.18 | 100.00 | Meningioma | 99.29 | 100.00 | 2.65E+05 | |
| **Unknown 4** | Meningioma | 12 | 3.69E+06 | Meningioma | 99.01 | | Meningioma | 99.31 | | | |
| **Unknown 4** | Meningioma | 12 | 2.54E+06 | Meningioma | 99.23 | | Meningioma | 99.34 | | | |
| **Unknown 4** | Meningioma | 11 | 3.53E+06 | Meningioma | 99.18 | | Meningioma | 99.31 | | | |
| **Unknown 4** | Meningioma | 2 | 3.65E+05 | Bad Data | - | | Bad Data | - | | | |
| **Unknown 4** | Meningioma | 9 | 4.55E+06 | Meningioma | 99.22 | | Meningioma | 99.3 | | | |
| **Unknown 4** | Meningioma | 10 | 3.47E+06 | Meningioma | 99.24 | | Meningioma | 99.37 | | | |
| **Unknown 4** | Meningioma | 9 | 4.33E+06 | Meningioma | 99.13 | | Meningioma | 99.25 | | | |
| **Unknown 4** | Meningioma | 11 | 4.02E+06 | Meningioma | 99.17 | | Meningioma | 99.36 | | | |
| **Unknown 4** | Meningioma | 12 | 4.75E+06 | Unclassifiable | - | | Meningioma | 99.24 | | | |
| **Unknown 4** | Meningioma | 10 | 2.86E+06 | Unclassifiable | - | | Meningioma | 99.27 | | | |
| **Unknown 4** | Meningioma | 12 | 2.31E+06 | Unclassifiable | - | | Meningioma | 99.29 | | | |
| **Unknown 5** | Meningioma | 12 | 8.92E+06 | Schwannoma | 98.26 | 36.36 | Meningioma | 97.13 | 90.91 | 2.48E+05 | Negative |
| **Unknown 5** | Meningioma | 13 | 2.98E+06 | Schwannoma | 98.5 | | Meningioma | 86.65 | | | |
| **Unknown 5** | Meningioma | 10 | 2.67E+06 | Meningioma | 92.13 | | Meningioma | 99.37 | | | |
| **Unknown 5** | Meningioma | 12 | 5.69E+06 | Schwannoma | 91.66 | | Meningioma | 99.38 | | | |
| **Unknown 5** | Meningioma | 10 | 4.73E+06 | Meningioma | 75.32 | | Meningioma | 98.91 | | | |
| **Unknown 5** | Meningioma | 13 | 1.56E+06 | Meningioma | 96.82 | | Meningioma | 99.08 | | | |
| **Unknown 5** | Meningioma | 12 | 5.43E+06 | Schwannoma | 98.35 | | Schwannoma | 90.66 | | | |
| **Unknown 5** | Meningioma | 11 | 3.57E+06 | Meningioma | 63.99 | | Meningioma | 97.89 | | | |
| **Unknown 5** | Meningioma | 11 | 2.38E+06 | Schwannoma | 98.91 | | Meningioma | 95.98 | | | |
| **Unknown 5** | Meningioma | 9 | 6.02E+05 | Schwannoma | 98.87 | | Meningioma | 97.95 | | | |
| **Unknown 5** | Meningioma | 10 | 2.17E+06 | Schwannoma | 96.33 | | Meningioma | 99.28 | | | |
| **Unknown 6** | Schwannoma | 10 | 4.15E+06 | Schwannoma | 98.54 | 100.00 | Schwannoma | 99.29 | 100.00 | 2.92E+05 | |
| **Unknown 6** | Schwannoma | 7 | 1.24E+06 | Schwannoma | 98.52 | | Schwannoma | 98.84 | | | |
| **Unknown 6** | Schwannoma | 10 | 1.86E+06 | Schwannoma | 99.38 | | Schwannoma | 99.35 | | | |
| **Unknown 6** | Schwannoma | 5 | 1.79E+06 | Schwannoma | 98.91 | | Schwannoma | 99.37 | | | |
| **Unknown 6** | Schwannoma | 8 | 1.88E+06 | Schwannoma | 98.61 | | Schwannoma | 99.36 | | | |
| **Unknown 6** | Schwannoma | 11 | 5.36E+06 | Schwannoma | 99.02 | | Schwannoma | 99.38 | | | |
| **Unknown 6** | Schwannoma | 6 | 2.98E+06 | Schwannoma | 98.97 | | Schwannoma | 99.3 | | | |
| **Unknown 7** | Metastatic | 10 | 1.61E+07 | Unclassifiable | - | 100.00 | Metastatic | 99.16 | 100.00 | 5.44E+05 | |
| **Unknown 7** | Metastatic | 11 | 1.26E+07 | Unclassifiable | - | | Metastatic | 98.42 | | | |
| **Unknown 7** | Metastatic | 11 | 4.57E+06 | Metastatic | 99.31 | | Metastatic | 99.33 | | | |
| **Unknown 8** | Schwannoma | 11 | 8.42E+06 | Schwannoma | 99.32 | 100.00 | Metastatic | 85.73 | 0.00 | N/A | |
| **Unknown 8** | Schwannoma | 12 | 6.57E+06 | Schwannoma | 99.16 | | Metastatic | 96.32 | | | |
| **Unknown 9** | Metastatic | 7 | 1.77E+06 | Metastatic | 99.13 | 100.00 | Schwannoma | 98.35 | 91.67 | 2.67E+05 | |
| **Unknown 9** | Metastatic | 11 | 2.26E+06 | Metastatic | 99.26 | | Metastatic | 98.86 | | | |
| **Unknown 9** | Metastatic | 9 | 5.05E+06 | Metastatic | 99.29 | | Metastatic | 99.38 | | | |
| **Unknown 9** | Metastatic | 11 | 2.18E+06 | Metastatic | 99.25 | | Metastatic | 99.18 | | | |
| **Unknown 9** | Metastatic | 9 | 4.38E+06 | Metastatic | 99.33 | | Metastatic | 99.27 | | | |
| **Unknown 9** | Metastatic | 13 | 1.43E+06 | Metastatic | 99.34 | | Metastatic | 99.27 | | | |
| **Unknown 9** | Metastatic | 7 | 2.63E+06 | Metastatic | 99.22 | | Metastatic | 99.3 | | | |
| **Unknown 9** | Metastatic | 7 | 1.16E+06 | Metastatic | 99.21 | | Metastatic | 99.13 | | | |
| **Unknown 9** | Metastatic | 1 | 1.22E+05 | Bad Data | - | | Bad Data | - | | | |
| **Unknown 9** | Metastatic | 12 | 2.47E+06 | Metastatic | 99.32 | | Metastatic | 99.17 | | | |
| **Unknown 9** | Metastatic | 9 | 4.21E+06 | Metastatic | 99.27 | | Metastatic | 99.04 | | | |
| **Unknown 9** | Metastatic | 11 | 5.21E+06 | Metastatic | 99.36 | | Metastatic | 99.36 | | | |
| **Unknown 9** | Metastatic | 10 | 2.99E+06 | Metastatic | 74.42 | | Metastatic | 98.69 | | | |
| **Unknown 10** | Meningioma | 8 | 2.20E+06 | Schwannoma | 62.38 | 28.57 | Schwannoma | 93.25 | 14.29 | 3.25E+05 | Negative |
| **Unknown 10** | Meningioma | 11 | 6.33E+06 | Schwannoma | 96.22 | | Schwannoma | 98.72 | | | |
| **Unknown 10** | Meningioma | 10 | 4.57E+06 | Schwannoma | 98.05 | | Metastatic | 79.44 | | | |
| **Unknown 10** | Meningioma | 11 | 4.78E+06 | Schwannoma | 96.51 | | Meningioma | 81.51 | | | |
| **Unknown 10** | Meningioma | 11 | 6.94E+06 | Meningioma | 96.48 | | Metastatic | 96.76 | | | |
| **Unknown 10** | Meningioma | 10 | 1.05E+07 | Meningioma | 96.99 | | Metastatic | 82.01 | | | |
| **Unknown 10** | Meningioma | 10 | 7.64E+06 | Schwannoma | 96.73 | | Schwannoma | 95.68 | | | |
| **Unknown 11** | Meningioma | 12 | 2.55E+06 | Meningioma | 97.28 | 100.00 | Meningioma | 98.74 | 100.00 | 3.41E+05 | |
| **Unknown 11** | Meningioma | 11 | 2.22E+06 | Meningioma | 98.58 | | Meningioma | 98.93 | | | |
| **Unknown 11** | Meningioma | 12 | 5.40E+06 | Meningioma | 98.72 | | Meningioma | 98.14 | | | |
| **Unknown 11** | Meningioma | 11 | 6.33E+06 | Meningioma | 99.31 | | Meningioma | 99.11 | | | |
| **Unknown 11** | Meningioma | 12 | 4.82E+06 | Meningioma | 99.31 | | Meningioma | 98.03 | | | |
| **Unknown 11** | Meningioma | 11 | 7.94E+06 | Meningioma | 97.85 | | Meningioma | 91.41 | | | |
| **Unknown 12** | Meningioma | 11 | 3.89E+06 | Unclassifiable | - | 100.00 | Meningioma | 99.38 | 100.00 | 3.24E+05 | |
| **Unknown 12** | Meningioma | 9 | 2.73E+06 | Meningioma | 99.32 | | Meningioma | 99.38 | | | |
| **Unknown 12** | Meningioma | 8 | 1.41E+06 | Meningioma | 98.63 | | Meningioma | 99.06 | | | |
| **Unknown 12** | Meningioma | 7 | 1.17E+06 | Meningioma | 99.22 | | Meningioma | 99.2 | | | |
| **Unknown 12** | Meningioma | 2 | 2.60E+05 | Bad Data | - | | Bad Data | - | | | |
| **Unknown 12** | Meningioma | 4 | 4.70E+05 | Meningioma | 99.37 | | Meningioma | 99.15 | | | |
| **Unknown 12** | Meningioma | 10 | 1.68E+06 | Meningioma | 99.01 | | Meningioma | 99.22 | | | |
| **Unknown 13** | Metastatic | 12 | 8.14E+06 | Metastatic | 99.37 | 100.00 | Metastatic | 99.33 | 100.00 | 2.64E+05 | |
| **Unknown 13** | Metastatic | 11 | 9.03E+06 | Metastatic | 99.17 | | Metastatic | 99.28 | | | |
| **Unknown 13** | Metastatic | 11 | 4.78E+06 | Metastatic | 99.37 | | Metastatic | 99.35 | | | |
| **Unknown 13** | Metastatic | 11 | 1.00E+07 | Metastatic | 99.21 | | Metastatic | 99.32 | | | |
| **Unknown 13** | Metastatic | 10 | 1.30E+07 | Metastatic | 99.28 | | Metastatic | 99.31 | | | |
| **Unknown 13** | Metastatic | 11 | 1.24E+07 | Metastatic | 99.35 | | Metastatic | 99.25 | | | |
| **Unknown 13** | Metastatic | 12 | 2.31E+07 | Metastatic | 99.22 | | Metastatic | 99.35 | | | |
| **Unknown 13** | Metastatic | 11 | 8.77E+06 | Metastatic | 99.17 | | Metastatic | 99.28 | | | |
| **Unknown 13** | Metastatic | 11 | 9.37E+06 | Metastatic | 99.2 | | Metastatic | 99.36 | | | |
| **Unknown 13** | Metastatic | 11 | 1.11E+07 | Metastatic | 98.57 | | Metastatic | 99.22 | | | |

| | | | | | | | | | | | |
|---|---|---|---|---|---|---|---|---|---|---|---|
| **Unknown 14** | Meningioma | 11 | 5.77E+06 | Meningioma | 99.26 | 100.00 | Meningioma | 99.22 | 100.00 | 2.23E+05 | |
| **Unknown 14** | Meningioma | 12 | 3.36E+06 | Meningioma | 99.3 | | Meningioma | 99.3 | | | |
| **Unknown 14** | Meningioma | 6 | 1.67E+06 | Meningioma | 99.36 | | Meningioma | 99.37 | | | |
| **Unknown 14** | Meningioma | 6 | 1.29E+06 | Meningioma | 99.23 | | Meningioma | 99.3 | | | |
| **Unknown 14** | Meningioma | 11 | 3.17E+06 | Meningioma | 99.32 | | Meningioma | 99.23 | | | |
| **Unknown 14** | Meningioma | 10 | 4.95E+06 | Meningioma | 99.09 | | Meningioma | 98.76 | | | |
| **Unknown 14** | Meningioma | 9 | 6.35E+06 | Meningioma | 99.22 | | Meningioma | 99.31 | | | |
| **Unknown 14** | Meningioma | 10 | 5.53E+06 | Meningioma | 99.2 | | Meningioma | 99.27 | | | |
| **Unknown 14** | Meningioma | 12 | 4.82E+06 | Unclassifiable | - | | Meningioma | 99.11 | | | |
| **Unknown 14** | Meningioma | 11 | 7.39E+06 | Meningioma | 99.02 | | Meningioma | 99.32 | | | |
| **Unknown 14** | Meningioma | 11 | 4.82E+06 | Unclassifiable | - | | Meningioma | 99.33 | | | |
| **Unknown 14** | Meningioma | 10 | 5.69E+06 | Unclassifiable | - | | Meningioma | 99.22 | | | |
| **Unknown 15** | Meningioma | 11 | 2.86E+06 | Meningioma | 99.38 | 100.00 | Meningioma | 99.34 | 100.00 | 2.77E+05 | |
| **Unknown 15** | Meningioma | 11 | 1.87E+06 | Meningioma | 99.29 | | Meningioma | 88.27 | | | |
| **Unknown 15** | Meningioma | 12 | 2.74E+06 | Meningioma | 99.36 | | Meningioma | 99.12 | | | |
| **Unknown 15** | Meningioma | 12 | 3.98E+06 | Meningioma | 99.35 | | Meningioma | 99.31 | | | |
| **Unknown 15** | Meningioma | 8 | 2.01E+06 | Meningioma | 99.36 | | Meningioma | 99.28 | | | |
| **Unknown 15** | Meningioma | 11 | 4.00E+06 | Meningioma | 99.36 | | Meningioma | 99.27 | | | |
| **Unknown 15** | Meningioma | 11 | 7.02E+06 | Meningioma | 99.33 | | Meningioma | 99.3 | | | |
| **Unknown 16** | Schwannoma | 11 | 8.54E+06 | Schwannoma | 99.28 | 100.00 | Schwannoma | 99.17 | 100.00 | 3.94E+05 | |
| **Unknown 16** | Schwannoma | 9 | 7.79E+06 | Schwannoma | 99.35 | | Schwannoma | 99.35 | | | |
| **Unknown 16** | Schwannoma | 12 | 1.11E+07 | Schwannoma | 99.23 | | Schwannoma | 99.14 | | | |
| **Unknown 16** | Schwannoma | 10 | 7.57E+06 | Schwannoma | 99.39 | | Schwannoma | 99.11 | | | |
| **Unknown 17** | Metastatic | 10 | 9.20E+05 | Metastatic | 99.25 | 100.00 | Metastatic | 99.31 | 100.00 | 3.21E+05 | |
| **Unknown 17** | Metastatic | 6 | 9.99E+05 | Metastatic | 99.23 | | Metastatic | 98.65 | | | |
| **Unknown 17** | Metastatic | 7 | 6.04E+05 | Metastatic | 99.24 | | Metastatic | 98.52 | | | |
| **Unknown 17** | Metastatic | 1 | 2.16E+05 | Bad Data | - | | Bad Data | - | | | |
| **Unknown 17** | Metastatic | 13 | 1.21E+06 | Metastatic | 99.25 | | Metastatic | 99.21 | | | |
| **Unknown 17** | Metastatic | 13 | 1.02E+06 | Metastatic | 99.27 | | Metastatic | 99.02 | | | |
| **Unknown 17** | Metastatic | 10 | 1.66E+06 | Metastatic | 99.27 | | Metastatic | 98.94 | | | |
| **Unknown 18** | Meningioma | 11 | 3.76E+06 | Meningioma | 99.35 | 100.00 | Meningioma | 99.1 | 100.00 | 2.61E+05 | |
| **Unknown 18** | Meningioma | 10 | 2.08E+06 | Meningioma | 99.18 | | Meningioma | 99.32 | | | |
| **Unknown 18** | Meningioma | 8 | 8.61E+05 | Unclassifiable | - | | Meningioma | 78.01 | | | |
| **Unknown 18** | Meningioma | 10 | 2.42E+06 | Meningioma | 99.09 | | Meningioma | 99.18 | | | |
| **Unknown 18** | Meningioma | 12 | 3.76E+06 | Meningioma | 98.85 | | Meningioma | 99.24 | | | |
| **Unknown 18** | Meningioma | 10 | 1.58E+06 | Meningioma | 99.23 | | Meningioma | 99.03 | | | |
| **Unknown 18** | Meningioma | 12 | 2.39E+06 | Meningioma | 99.12 | | Meningioma | 99 | | | |
| **Unknown 18** | Meningioma | 11 | 3.26E+06 | Meningioma | 99.03 | | Meningioma | 98.76 | | | |
| **Unknown 18** | Meningioma | 8 | 1.31E+06 | Meningioma | 98.95 | | Meningioma | 98.65 | | | |
| **Unknown 18** | Meningioma | 13 | 4.90E+05 | Meningioma | 98.66 | | Meningioma | 99.17 | | | |
| **Unknown 18** | Meningioma | 12 | 3.38E+06 | Meningioma | 99.03 | | Meningioma | 99.01 | | | |
| **Unknown 18** | Meningioma | 2 | 2.23E+05 | Bad Data | - | | Bad Data | - | | | |
| **Unknown 19** | Meningioma | 11 | 4.34E+06 | Meningioma | 99.34 | 100.00 | Meningioma | 99.38 | 100.00 | 3.12E+05 | |
| **Unknown 19** | Meningioma | 7 | 1.69E+06 | Meningioma | 99.16 | | Meningioma | 99.34 | | | |
| **Unknown 19** | Meningioma | 6 | 1.09E+06 | Meningioma | 99.28 | | Meningioma | 99.12 | | | |
| **Unknown 19** | Meningioma | 9 | 1.23E+06 | Meningioma | 99.38 | | Meningioma | 99.24 | | | |
| **Unknown 19** | Meningioma | 11 | 3.09E+06 | Meningioma | 99.38 | | Meningioma | 99.38 | | | |
| **Unknown 19** | Meningioma | 11 | 2.42E+06 | Meningioma | 99.33 | | Meningioma | 99.33 | | | |
| **Unknown 19** | Meningioma | 2 | 2.58E+05 | Bad Data | - | | Bad Data | - | | | |
| **Unknown 19** | Meningioma | 8 | 7.72E+05 | Meningioma | 99.09 | | Meningioma | 99.28 | | | |
| **Unknown 20** | Meningioma | 3 | 3.20E+05 | Bad Data | - | 0.00 | Bad Data | - | 0.00 | 4.02E+05 | Negative |
| **Unknown 20** | Meningioma | 9 | 7.62E+05 | Schwannoma | 97.6 | | Schwannoma | 99.07 | | | |
| **Unknown 20** | Meningioma | 12 | 2.35E+06 | Schwannoma | 99.08 | | Schwannoma | 98.98 | | | |
| **Unknown 20** | Meningioma | 12 | 4.87E+06 | Schwannoma | 99.36 | | Schwannoma | 99.32 | | | |
| **Unknown 20** | Meningioma | 7 | 1.90E+06 | Schwannoma | 99.34 | | Schwannoma | 98.89 | | | |
| **Unknown 20** | Meningioma | 9 | 3.05E+06 | Schwannoma | 99.33 | | Schwannoma | 99.33 | | | |
| **Unknown 21** | Schwannoma | 8 | 5.06E+06 | Schwannoma | 99.35 | 90.00 | Schwannoma | 99.17 | 90.00 | 3.00E+05 | |
| **Unknown 21** | Schwannoma | 10 | 6.01E+06 | Schwannoma | 99.33 | | Schwannoma | 99.14 | | | |
| **Unknown 21** | Schwannoma | 11 | 5.24E+06 | Schwannoma | 99.34 | | Schwannoma | 99.35 | | | |
| **Unknown 21** | Schwannoma | 10 | 7.53E+06 | Schwannoma | 99.29 | | Schwannoma | 98.11 | | | |
| **Unknown 21** | Schwannoma | 12 | 9.35E+06 | Schwannoma | 99.35 | | Schwannoma | 99.07 | | | |
| **Unknown 21** | Schwannoma | 13 | 2.53E+06 | Schwannoma | 99.38 | | Schwannoma | 98.88 | | | |
| **Unknown 21** | Schwannoma | 4 | 5.17E+05 | Metastatic | 89.74 | | Meningioma | 82.22 | | | |
| **Unknown 21** | Schwannoma | 3 | 5.48E+05 | Bad Data | - | | Bad Data | - | | | |
| **Unknown 21** | Schwannoma | 6 | 7.33E+05 | Schwannoma | 97.01 | | Schwannoma | 99.27 | | | |
| **Unknown 21** | Schwannoma | 10 | 2.71E+06 | Schwannoma | 99.29 | | Schwannoma | 96.61 | | | |
| **Unknown 21** | Schwannoma | 12 | 9.09E+06 | Schwannoma | 99.34 | | Schwannoma | 99.29 | | | |
| **Unknown 22** | Schwannoma | 11 | 4.72E+06 | Schwannoma | 99.36 | 100.00 | Schwannoma | 99.29 | 100.00 | 2.79E+05 | |
| **Unknown 22** | Schwannoma | 12 | 4.87E+06 | Schwannoma | 99.38 | | Schwannoma | 98.67 | | | |
| **Unknown 22** | Schwannoma | 11 | 5.36E+06 | Schwannoma | 99.38 | | Schwannoma | 99.37 | | | |
| **Unknown 22** | Schwannoma | 12 | 9.15E+06 | Schwannoma | 99.33 | | Schwannoma | 98.75 | | | |
| **Unknown 22** | Schwannoma | 12 | 3.31E+06 | Schwannoma | 99.25 | | Schwannoma | 99.29 | | | |
| **Unknown 22** | Schwannoma | 2 | 4.58E+05 | Bad Data | - | | Bad Data | - | | | |
| **Unknown 22** | Schwannoma | 12 | 1.01E+07 | Schwannoma | 99.37 | | Schwannoma | 99.35 | | | |
| **Unknown 22** | Schwannoma | 12 | 7.66E+06 | Schwannoma | 99.36 | | Schwannoma | 99.36 | | | |
| **Unknown 22** | Schwannoma | 10 | 6.46E+06 | Schwannoma | 99.38 | | Schwannoma | 99.11 | | | |
| **Unknown 23** | Meningioma | 11 | 1.17E+07 | Meningioma | 98.6 | 88.89 | Meningioma | 93.62 | 88.89 | 2.49E+05 | |
| **Unknown 23** | Meningioma | 10 | 3.73E+06 | Meningioma | 99.1 | | Meningioma | 98.97 | | | |
| **Unknown 23** | Meningioma | 10 | 5.02E+06 | Meningioma | 98.06 | | Meningioma | 98.94 | | | |
| **Unknown 23** | Meningioma | 12 | 5.58E+06 | Meningioma | 99.16 | | Meningioma | 98.87 | | | |
| **Unknown 23** | Meningioma | 11 | 4.04E+06 | Meningioma | 99.32 | | Meningioma | 99.37 | | | |
| **Unknown 23** | Meningioma | 11 | 4.12E+06 | Meningioma | 97.17 | | Meningioma | 98.56 | | | |
| **Unknown 23** | Meningioma | 11 | 3.29E+06 | Schwannoma | 90.12 | | Metastatic | 59.06 | | | |
| **Unknown 23** | Meningioma | 11 | 6.87E+06 | Meningioma | 99.22 | | Meningioma | 98.78 | | | |
| **Unknown 23** | Meningioma | 12 | 1.38E+07 | Meningioma | 99.35 | | Meningioma | 99.33 | | | |
| **Unknown 24** | Metastatic | 12 | 7.67E+06 | Metastatic | 99.38 | 100.00 | Metastatic | 99.29 | 90.91 | 2.32E+05 | |
| **Unknown 24** | Metastatic | 11 | 8.35E+06 | Metastatic | 99.36 | | Metastatic | 99.35 | | | |
| **Unknown 24** | Metastatic | 10 | 6.10E+06 | Metastatic | 99.37 | | Metastatic | 79.64 | | | |
| **Unknown 24** | Metastatic | 10 | 4.06E+06 | Metastatic | 99.37 | | Metastatic | 85.32 | | | |
| **Unknown 24** | Metastatic | 10 | 1.86E+06 | Metastatic | 99.34 | | Metastatic | 99.24 | | | |
| **Unknown 24** | Metastatic | 10 | 2.17E+06 | Metastatic | 99.35 | | Metastatic | 99.01 | | | |
| **Unknown 24** | Metastatic | 11 | 4.65E+06 | Metastatic | 99.33 | | Metastatic | 86.5 | | | |
| **Unknown 24** | Metastatic | 11 | 7.65E+06 | Metastatic | 99.38 | | Metastatic | 90.98 | | | |
| **Unknown 24** | Metastatic | 10 | 2.94E+06 | Metastatic | 99.38 | | Metastatic | 99.04 | | | |
| **Unknown 24** | Metastatic | 11 | 7.64E+06 | Metastatic | 99.34 | | Metastatic | 93.27 | | | |
| **Unknown 24** | Metastatic | 12 | 9.64E+06 | Metastatic | 99.37 | | Schwannoma | 76.27 | | | |
| **Unknown 25** | Schwannoma | 11 | 7.10E+06 | Schwannoma | 80.94 | 100.00 | Schwannoma | 83.47 | 100.00 | 3.69E+05 | |
| **Unknown 25** | Schwannoma | 11 | 4.54E+06 | Schwannoma | 96.54 | | Schwannoma | 85.16 | | | |
| **Unknown 25** | Schwannoma | 9 | 5.26E+06 | Schwannoma | 99.36 | | Schwannoma | 99.3 | | | |
| **Unknown 25** | Schwannoma | 8 | 2.00E+06 | Schwannoma | 99.34 | | Schwannoma | 99.22 | | | |
| **Unknown 25** | Schwannoma | 12 | 7.06E+06 | Schwannoma | 99.07 | | Schwannoma | 99.38 | | | |

| | | | | | | | | | | | |
|---|---|---|---|---|---|---|---|---|---|---|---|
| **Unknown 26** | Schwannoma | 9 | 1.90E+06 | Schwannoma | 98.14 | 83.33 | Schwannoma | 96.55 | 100.00 | 2.77E+05 | |
| **Unknown 26** | Schwannoma | 7 | 7.36E+05 | Metastatic | 82.4 | | Schwannoma | 96.64 | | | |
| **Unknown 26** | Schwannoma | 8 | 1.88E+06 | Schwannoma | 98.26 | | Schwannoma | 97.97 | | | |
| **Unknown 26** | Schwannoma | 3 | 6.06E+05 | Bad Data | - | | Bad Data | - | | | |
| **Unknown 26** | Schwannoma | 4 | 6.60E+05 | Schwannoma | 98.06 | | Schwannoma | 96.78 | | | |
| **Unknown 26** | Schwannoma | 8 | 1.07E+06 | Schwannoma | 96.64 | | Schwannoma | 95.71 | | | |
| **Unknown 26** | Schwannoma | 3 | 3.17E+05 | Bad Data | - | | Bad Data | - | | | |
| **Unknown 26** | Schwannoma | 10 | 2.59E+06 | Schwannoma | 97.48 | | Schwannoma | 98.23 | | | |
| **Unknown 27** | Schwannoma | 10 | 1.20E+06 | Schwannoma | 99.33 | 100.00 | Schwannoma | 99.37 | 100.00 | 3.03E+05 | |
| **Unknown 27** | Schwannoma | 10 | 1.98E+06 | Schwannoma | 99.35 | | Schwannoma | 99.13 | | | |
| **Unknown 27** | Schwannoma | 11 | 7.42E+06 | Schwannoma | 99.31 | | Schwannoma | 99.27 | | | |
| **Unknown 27** | Schwannoma | 11 | 5.25E+06 | Schwannoma | 99.09 | | Schwannoma | 99.23 | | | |
| **Unknown 27** | Schwannoma | 11 | 7.15E+06 | Schwannoma | 99.33 | | Schwannoma | 99.29 | | | |
| **Unknown 27** | Schwannoma | 12 | 6.29E+06 | Schwannoma | 99.2 | | Schwannoma | 98.26 | | | |
| **Unknown 27** | Schwannoma | 13 | 5.46E+06 | Schwannoma | 99.18 | | Schwannoma | 99.04 | | | |
| **Unknown 28** | Schwannoma | 12 | 5.14E+06 | Schwannoma | 98.35 | 66.67 | Metastatic | 94.24 | 44.44 | 2.75E+05 | |
| **Unknown 28** | Schwannoma | 11 | 2.59E+06 | Schwannoma | 98.52 | | Metastatic | 87.02 | | | |
| **Unknown 28** | Schwannoma | 11 | 3.79E+06 | Schwannoma | 98.71 | | Metastatic | 97.43 | | | |
| **Unknown 28** | Schwannoma | 11 | 2.98E+06 | Metastatic | 96 | | Metastatic | 98.79 | | | |
| **Unknown 28** | Schwannoma | 10 | 1.40E+06 | Schwannoma | 92.49 | | Schwannoma | 67.31 | | | |
| **Unknown 28** | Schwannoma | 13 | 3.17E+06 | Schwannoma | 93.55 | | Metastatic | 95.02 | | | |
| **Unknown 28** | Schwannoma | 11 | 1.70E+06 | Metastatic | 99.07 | | Schwannoma | 97.96 | | | |
| **Unknown 28** | Schwannoma | 8 | 8.72E+05 | Metastatic | 88.97 | | Schwannoma | 97.98 | | | |
| **Unknown 28** | Schwannoma | 11 | 2.17E+06 | Schwannoma | 98.31 | | Schwannoma | 98.45 | | | |
| **Unknown 29** | Metastatic | 11 | 1.64E+06 | Metastatic | 99.24 | 100.00 | Metastatic | 98.19 | 100.00 | 2.26E+05 | |
| **Unknown 29** | Metastatic | 12 | 6.75E+05 | Metastatic | 99.28 | | Metastatic | 91.34 | | | |
| **Unknown 29** | Metastatic | 6 | 1.09E+06 | Metastatic | 99.27 | | Metastatic | 97.92 | | | |
| **Unknown 29** | Metastatic | 13 | 1.51E+06 | Metastatic | 99.3 | | Metastatic | 99.28 | | | |
| **Unknown 29** | Metastatic | 3 | 6.10E+05 | Bad Data | - | | Bad Data | - | | | |
| **Unknown 29** | Metastatic | 5 | 7.62E+05 | Metastatic | 99.33 | | Metastatic | 99.36 | | | |
| **Unknown 29** | Metastatic | 3 | 5.45E+05 | Bad Data | - | | Bad Data | - | | | |
| **Unknown 29** | Metastatic | 3 | 5.25E+05 | Bad Data | - | | Bad Data | - | | | |
| **Unknown 29** | Metastatic | 9 | 2.27E+06 | Metastatic | 98.75 | | Metastatic | 98.87 | | | |
| **Unknown 29** | Metastatic | 8 | 1.48E+06 | Metastatic | 99.35 | | Metastatic | 72.54 | | | |
| **Unknown 29** | Metastatic | 7 | 1.17E+06 | Metastatic | 99.38 | | Metastatic | 95.63 | | | |
| **Unknown 29** | Metastatic | 9 | 1.54E+06 | Metastatic | 99.3 | | Metastatic | 99.18 | | | |
| **Unknown 30** | Schwannoma | 8 | 8.20E+05 | Schwannoma | 98.99 | 100.00 | Schwannoma | 98.92 | 100.00 | 2.49E+05 | |
| **Unknown 30** | Schwannoma | 10 | 1.12E+06 | Schwannoma | 93.77 | | Schwannoma | 99.24 | | | |
| **Unknown 30** | Schwannoma | 11 | 3.25E+06 | Schwannoma | 99.37 | | Schwannoma | 99.37 | | | |
| **Unknown 30** | Schwannoma | 7 | 1.45E+06 | Schwannoma | 99.36 | | Schwannoma | 99.21 | | | |
| **Unknown 30** | Schwannoma | 10 | 3.96E+06 | Schwannoma | 99.28 | | Schwannoma | 99.32 | | | |
| **Unknown 30** | Schwannoma | 10 | 2.25E+06 | Schwannoma | 99.34 | | Schwannoma | 99.18 | | | |
| **Unknown 30** | Schwannoma | 12 | 4.16E+06 | Schwannoma | 99.35 | | Schwannoma | 99.25 | | | |
| **Unknown 30** | Schwannoma | 12 | 2.35E+06 | Schwannoma | 99.3 | | Schwannoma | 99.03 | | | |
| **Unknown 30** | Schwannoma | 11 | 3.60E+06 | Schwannoma | 99.32 | | Schwannoma | 99.39 | | | |
| **Unknown 31** | Schwannoma | 12 | 4.94E+06 | Schwannoma | 99.33 | 100.00 | Schwannoma | 99.3 | 83.33 | 3.12E+05 | |
| **Unknown 31** | Schwannoma | 11 | 4.64E+06 | Schwannoma | 96.99 | | Schwannoma | 99.29 | | | |
| **Unknown 31** | Schwannoma | 12 | 3.64E+06 | Schwannoma | 98.99 | | Schwannoma | 99.17 | | | |
| **Unknown 31** | Schwannoma | 5 | 1.18E+06 | Schwannoma | 97.5 | | Meningioma | 96.52 | | | |
| **Unknown 31** | Schwannoma | 10 | 1.77E+06 | Schwannoma | 98.91 | | Schwannoma | 98.16 | | | |
| **Unknown 31** | Schwannoma | 11 | 9.02E+06 | Schwannoma | 98.68 | | Schwannoma | 98.15 | | | |
| **Unknown 32** | Schwannoma | 11 | 3.02E+06 | Schwannoma | 99.35 | 100.00 | Schwannoma | 99.3 | 100.00 | 2.81E+05 | |
| **Unknown 32** | Schwannoma | 11 | 3.05E+06 | Schwannoma | 98.69 | | Schwannoma | 99.33 | | | |
| **Unknown 32** | Schwannoma | 11 | 3.47E+06 | Schwannoma | 99.32 | | Schwannoma | 99.31 | | | |
| **Unknown 32** | Schwannoma | 10 | 1.55E+06 | Schwannoma | 99.31 | | Schwannoma | 99.28 | | | |
| **Unknown 32** | Schwannoma | 10 | 1.79E+06 | Schwannoma | 98.64 | | Schwannoma | 84.54 | | | |
| **Unknown 32** | Schwannoma | 8 | 9.53E+05 | Schwannoma | 99.25 | | Schwannoma | 95.31 | | | |
| **Unknown 32** | Schwannoma | 10 | 1.70E+06 | Schwannoma | 99.33 | | Schwannoma | 97.88 | | | |
| **Unknown 32** | Schwannoma | 11 | 4.00E+06 | Schwannoma | 99.13 | | Schwannoma | 99.14 | | | |
| **Unknown 33** | Metastatic | 11 | 4.34E+06 | Unclassifiable | - | 100.00 | Metastatic | 69.41 | 90.00 | 2.42E+05 | |
| **Unknown 33** | Metastatic | 5 | 1.29E+06 | Metastatic | 99.34 | | Metastatic | 98.41 | | | |
| **Unknown 33** | Metastatic | 7 | 2.98E+06 | Metastatic | 98.79 | | Schwannoma | 77.44 | | | |
| **Unknown 33** | Metastatic | 11 | 7.14E+06 | Metastatic | 81.44 | | Metastatic | 97.94 | | | |
| **Unknown 33** | Metastatic | 10 | 5.34E+06 | Metastatic | 97.33 | | Metastatic | 99.19 | | | |
| **Unknown 33** | Metastatic | 11 | 6.46E+06 | Metastatic | 92.57 | | Metastatic | 99.37 | | | |
| **Unknown 33** | Metastatic | 9 | 4.78E+06 | Metastatic | 97.96 | | Metastatic | 97.93 | | | |
| **Unknown 33** | Metastatic | 11 | 7.86E+06 | Metastatic | 77.52 | | Metastatic | 99.32 | | | |
| **Unknown 33** | Metastatic | 10 | 7.94E+06 | Metastatic | 98.29 | | Metastatic | 75.6 | | | |
| **Unknown 33** | Metastatic | 11 | 8.79E+06 | Metastatic | 97.69 | | Metastatic | 98.52 | | | |
| **Unknown 34** | Schwannoma | 8 | 3.87E+06 | Schwannoma | 99.36 | 100.00 | Schwannoma | 99.05 | 100.00 | N/A | |
| **Unknown 34** | Schwannoma | 10 | 1.06E+07 | Schwannoma | 99.32 | | Schwannoma | 97.86 | | | |
| **Unknown 35** | Metastatic | 13 | 2.28E+06 | Metastatic | 99.14 | 100.00 | Metastatic | 98.71 | 100.00 | 3.09E+05 | |
| **Unknown 35** | Metastatic | 10 | 1.83E+06 | Metastatic | 98.33 | | Metastatic | 98.03 | | | |
| **Unknown 35** | Metastatic | 1 | 1.73E+05 | Bad Data | - | | Bad Data | - | | | |
| **Unknown 35** | Metastatic | 8 | 1.42E+06 | Metastatic | 98.68 | | Metastatic | 96.35 | | | |
| **Unknown 35** | Metastatic | 8 | 1.18E+06 | Metastatic | 99.28 | | Metastatic | 98.03 | | | |
| **Unknown 35** | Metastatic | 7 | 1.76E+06 | Metastatic | 99.25 | | Metastatic | 98.26 | | | |
| **Unknown 35** | Metastatic | 8 | 1.28E+06 | Metastatic | 99.07 | | Metastatic | 99.13 | | | |
| **Unknown 35** | Metastatic | 8 | 1.08E+06 | Metastatic | 99.25 | | Metastatic | 99.3 | | | |
| **Unknown 36** | Schwannoma | 11 | 1.31E+06 | Schwannoma | 91.47 | 100.00 | Metastatic | 94.36 | 100.00 | 3.21E+05 | |
| **Unknown 36** | Schwannoma | 8 | 3.85E+06 | Schwannoma | 99.29 | | Schwannoma | 98.52 | | | |
| **Unknown 36** | Schwannoma | 11 | 4.67E+06 | Schwannoma | 99.25 | | Schwannoma | 99.04 | | | |
| **Unknown 36** | Schwannoma | 11 | 2.73E+06 | Schwannoma | 98.61 | | Schwannoma | 98.44 | | | |
| **Unknown 36** | Schwannoma | 10 | 1.51E+06 | Schwannoma | 99.28 | | Schwannoma | 98.78 | | | |
| **Unknown 36** | Schwannoma | 11 | 2.82E+06 | Schwannoma | 98.99 | | Schwannoma | 98.72 | | | |
| **Unknown 36** | Schwannoma | 7 | 1.25E+06 | Schwannoma | 98.94 | | Schwannoma | 99.33 | | | |
| **Unknown 36** | Schwannoma | 10 | 1.36E+06 | Schwannoma | 99.37 | | Schwannoma | 98.83 | | | |
| **Unknown 36** | Schwannoma | 13 | 4.03E+06 | Unclassifiable | - | | Schwannoma | 97.76 | | | |
| **Unknown 37** | Schwannoma | 10 | 2.57E+06 | Meningioma | 98.55 | 0.00 | Meningioma | 99.34 | 12.50 | 3.01E+05 | Positive |
| **Unknown 37** | Schwannoma | 10 | 4.40E+06 | Meningioma | 98.99 | | Meningioma | 99.29 | | | |
| **Unknown 37** | Schwannoma | 10 | 3.57E+06 | Meningioma | 97.39 | | Meningioma | 98.28 | | | |
| **Unknown 37** | Schwannoma | 10 | 2.11E+06 | Meningioma | 98.16 | | Meningioma | 93.24 | | | |
| **Unknown 37** | Schwannoma | 10 | 3.10E+06 | Meningioma | 95.75 | | Meningioma | 77.28 | | | |
| **Unknown 37** | Schwannoma | 11 | 2.78E+06 | Meningioma | 96.47 | | Meningioma | 98.82 | | | |
| **Unknown 37** | Schwannoma | 12 | 3.39E+06 | Meningioma | 98.97 | | Meningioma | 98.81 | | | |
| **Unknown 37** | Schwannoma | 9 | 1.39E+06 | Meningioma | 94.37 | | Schwannoma | 88.01 | | | |

| | | | | | | | | | | |
|---|---|---|---|---|---|---|---|---|---|---|
| **Unknown 38** | Metastatic | 4 | 5.88E+05 | Schwannoma | 96.33 | 66.67 | Metastatic | 98.58 | 33.33 | 2.65E+05 |
| **Unknown 38** | Metastatic | 10 | 1.81E+06 | Metastatic | 77.35 | | Schwannoma | 98.6 | | |
| **Unknown 38** | Metastatic | 12 | 2.71E+06 | Schwannoma | 97.66 | | Schwannoma | 96.53 | | |
| **Unknown 38** | Metastatic | 10 | 3.15E+06 | Schwannoma | 82 | | Schwannoma | 99.13 | | |
| **Unknown 38** | Metastatic | 11 | 5.22E+06 | Metastatic | 93.27 | | Schwannoma | 98.23 | | |
| **Unknown 38** | Metastatic | 11 | 4.62E+06 | Metastatic | 97.24 | | Schwannoma | 96.98 | | |
| **Unknown 38** | Metastatic | 11 | 2.79E+06 | Metastatic | 88 | | Metastatic | 85.94 | | |
| **Unknown 38** | Metastatic | 11 | 3.70E+06 | Metastatic | 95.81 | | Metastatic | 60.11 | | |
| **Unknown 38** | Metastatic | 11 | 2.55E+06 | Metastatic | 96.18 | | Schwannoma | 68.28 | | |
| **Unknown 39** | Schwannoma | 11 | 4.23E+06 | Schwannoma | 98.33 | 100.00 | Schwannoma | 98.95 | 100.00 | 2.76E+05 |
| **Unknown 39** | Schwannoma | 11 | 3.55E+06 | Schwannoma | 97.94 | | Schwannoma | 99.33 | | |
| **Unknown 39** | Schwannoma | 11 | 1.48E+06 | Schwannoma | 99.03 | | Schwannoma | 99.29 | | |
| **Unknown 39** | Schwannoma | 13 | 2.22E+06 | Schwannoma | 98.6 | | Schwannoma | 99.08 | | |
| **Unknown 39** | Schwannoma | 9 | 2.00E+06 | Schwannoma | 99.09 | | Schwannoma | 99.12 | | |
| **Unknown 39** | Schwannoma | 8 | 4.91E+05 | Schwannoma | 98.21 | | Schwannoma | 98.58 | | |
| **Unknown 39** | Schwannoma | 11 | 3.40E+06 | Schwannoma | 98.62 | | Schwannoma | 99.16 | | |
| **Unknown 39** | Schwannoma | 11 | 4.00E+06 | Schwannoma | 99.19 | | Schwannoma | 99.11 | | |
| **Unknown 39** | Schwannoma | 11 | 2.94E+06 | Schwannoma | 98.45 | | Schwannoma | 99.29 | | |
| **Unknown 40** | Schwannoma | 10 | 3.75E+06 | Schwannoma | 98.91 | 100.00 | Schwannoma | 98.84 | 100.00 | 2.71E+05 |
| **Unknown 40** | Schwannoma | 11 | 3.39E+06 | Schwannoma | 98.9 | | Schwannoma | 98.96 | | |
| **Unknown 40** | Schwannoma | 5 | 9.50E+05 | Schwannoma | 99.29 | | Schwannoma | 98.86 | | |
| **Unknown 40** | Schwannoma | 12 | 4.85E+06 | Schwannoma | 98.85 | | Schwannoma | 98.25 | | |
| **Unknown 40** | Schwannoma | 12 | 4.45E+06 | Schwannoma | 99.03 | | Schwannoma | 98.85 | | |
| **Unknown 40** | Schwannoma | 11 | 2.91E+06 | Schwannoma | 98.94 | | Schwannoma | 98.79 | | |
| **Unknown 40** | Schwannoma | 11 | 2.96E+06 | Schwannoma | 98.9 | | Schwannoma | 98.64 | | |
| **Unknown 40** | Schwannoma | 11 | 3.18E+06 | Schwannoma | 98.98 | | Schwannoma | 99.08 | | |
| **Unknown 40** | Schwannoma | 12 | 5.39E+06 | Schwannoma | 99.23 | | Schwannoma | 98.56 | | |
| **Unknown 41** | Metastatic | 10 | 2.26E+06 | Metastatic | 99.27 | 100.00 | Metastatic | 98.76 | 100.00 | 2.54E+05 |
| **Unknown 41** | Metastatic | 11 | 1.77E+06 | Metastatic | 99.27 | | Metastatic | 98.65 | | |
| **Unknown 41** | Metastatic | 12 | 2.61E+06 | Metastatic | 99.24 | | Metastatic | 98.71 | | |
| **Unknown 41** | Metastatic | 12 | 1.62E+06 | Unclassifiable | - | | Metastatic | 99.07 | | |
| **Unknown 41** | Metastatic | 11 | 2.03E+06 | Metastatic | 99.25 | | Metastatic | 99.04 | | |
| **Unknown 41** | Metastatic | 7 | 7.07E+05 | Metastatic | 99.35 | | Metastatic | 99.16 | | |
| **Unknown 41** | Metastatic | 8 | 2.17E+06 | Metastatic | 99.25 | | Metastatic | 99 | | |
| **Unknown 41** | Metastatic | 8 | 2.90E+06 | Metastatic | 99.38 | | Metastatic | 98.54 | | |
| **Unknown 41** | Metastatic | 9 | 2.13E+06 | Metastatic | 99.33 | | Metastatic | 98.52 | | |
| **Unknown 41** | Metastatic | 9 | 1.93E+06 | Metastatic | 99.37 | | Metastatic | 98.46 | | |
| **Unknown 41** | Metastatic | 6 | 1.02E+06 | Metastatic | 99.32 | | Metastatic | 99.05 | | |
| **Unknown 42** | Meningioma | 11 | 4.74E+06 | Meningioma | 99.22 | 100.00 | Meningioma | 99.09 | 100.00 | 2.51E+05 |
| **Unknown 42** | Meningioma | 11 | 4.18E+06 | Meningioma | 99.31 | | Meningioma | 99.02 | | |
| **Unknown 42** | Meningioma | 7 | 1.40E+06 | Meningioma | 99.36 | | Meningioma | 99.16 | | |
| **Unknown 42** | Meningioma | 11 | 3.47E+06 | Meningioma | 99.29 | | Meningioma | 99.19 | | |
| **Unknown 42** | Meningioma | 10 | 3.91E+06 | Meningioma | 99.32 | | Meningioma | 99.19 | | |
| **Unknown 42** | Meningioma | 13 | 2.92E+06 | Meningioma | 99.35 | | Meningioma | 99.34 | | |
| **Unknown 42** | Meningioma | 13 | 1.61E+06 | Meningioma | 99.27 | | Meningioma | 99.34 | | |
| **Unknown 42** | Meningioma | 10 | 4.97E+06 | Meningioma | 99.33 | | Meningioma | 99.11 | | |
| **Unknown 42** | Meningioma | 11 | 6.20E+06 | Meningioma | 99.26 | | Meningioma | 99.06 | | |
| **Unknown 42** | Meningioma | 8 | 1.10E+06 | Meningioma | 99.22 | | Meningioma | 99.22 | | |
| **Unknown 43** | Meningioma | 12 | 4.95E+06 | Meningioma | 99.29 | 100.00 | Meningioma | 98.81 | 100.00 | 3.17E+05 |
| **Unknown 43** | Meningioma | 12 | 7.82E+06 | Meningioma | 99.27 | | Meningioma | 99.17 | | |
| **Unknown 43** | Meningioma | 13 | 3.36E+06 | Meningioma | 99.31 | | Meningioma | 99.28 | | |
| **Unknown 43** | Meningioma | 13 | 6.51E+06 | Meningioma | 99.38 | | Meningioma | 98.9 | | |
| **Unknown 43** | Meningioma | 9 | 6.68E+06 | Meningioma | 99.37 | | Meningioma | 99.05 | | |
| **Unknown 43** | Meningioma | 12 | 6.73E+06 | Meningioma | 99.25 | | Meningioma | 99.24 | | |
| **Unknown 44** | Metastatic | 11 | 3.26E+06 | Metastatic | 99.35 | 100.00 | Metastatic | 99.31 | 100.00 | 2.41E+05 |
| **Unknown 44** | Metastatic | 11 | 2.84E+06 | Metastatic | 99.19 | | Metastatic | 99.27 | | |
| **Unknown 44** | Metastatic | 6 | 1.31E+06 | Metastatic | 99.35 | | Metastatic | 99 | | |
| **Unknown 44** | Metastatic | 5 | 5.05E+05 | Metastatic | 99.2 | | Metastatic | 99.14 | | |
| **Unknown 44** | Metastatic | 13 | 1.21E+06 | Metastatic | 99.26 | | Metastatic | 98.43 | | |
| **Unknown 44** | Metastatic | 10 | 8.64E+05 | Metastatic | 99.32 | | Metastatic | 99.27 | | |
| **Unknown 44** | Metastatic | 8 | 1.38E+06 | Metastatic | 99.33 | | Metastatic | 97.95 | | |
| **Unknown 44** | Metastatic | 12 | 2.75E+06 | Metastatic | 99.16 | | Metastatic | 99.01 | | |
| **Unknown 44** | Metastatic | 11 | 3.49E+06 | Metastatic | 99.1 | | Metastatic | 99.16 | | |
| **Unknown 44** | Metastatic | 11 | 5.27E+06 | Metastatic | 99.25 | | Metastatic | 99.15 | | |
| **Unknown 45** | Metastatic | 8 | 1.20E+06 | Metastatic | 99.09 | 100.00 | Metastatic | 98.44 | 100.00 | 2.38E+05 |
| **Unknown 45** | Metastatic | 9 | 2.36E+06 | Metastatic | 98.95 | | Metastatic | 99.08 | | |
| **Unknown 45** | Metastatic | 8 | 1.58E+06 | Metastatic | 99.21 | | Metastatic | 99.36 | | |
| **Unknown 45** | Metastatic | 9 | 1.41E+06 | Metastatic | 99.23 | | Metastatic | 99.29 | | |
| **Unknown 45** | Metastatic | 10 | 1.64E+06 | Metastatic | 99.18 | | Metastatic | 99.34 | | |
| **Unknown 45** | Metastatic | 9 | 1.85E+06 | Metastatic | 99.3 | | Metastatic | 99.12 | | |
| **Unknown 45** | Metastatic | 10 | 1.50E+06 | Metastatic | 98.9 | | Metastatic | 98.89 | | |
| **Unknown 45** | Metastatic | 10 | 1.69E+06 | Metastatic | 99.07 | | Metastatic | 98.95 | | |
| **Unknown 45** | Metastatic | 10 | 1.32E+06 | Metastatic | 99.21 | | Metastatic | 99.32 | | |
| **Unknown 45** | Metastatic | 3 | 4.35E+05 | Bad Data | - | | Bad Data | - | | |
| **Unknown 45** | Metastatic | 5 | 7.55E+05 | Metastatic | 99.26 | | Metastatic | 99.23 | | |
| **Unknown 46** | Schwannoma | 8 | 4.80E+06 | Schwannoma | 99.31 | 100.00 | Schwannoma | 99.34 | 100.00 | N/A |
| **Unknown 46** | Schwannoma | 10 | 6.30E+06 | Schwannoma | 99.36 | | Schwannoma | 99.21 | | |
| **Unknown 47** | Metastatic | 9 | 3.83E+06 | Metastatic | 99.36 | 100.00 | Metastatic | 99.2 | 100.00 | 3.54E+05 |
| **Unknown 47** | Metastatic | 11 | 4.98E+06 | Metastatic | 99.34 | | Metastatic | 98.81 | | |
| **Unknown 47** | Metastatic | 11 | 5.14E+06 | Metastatic | 99.3 | | Metastatic | 97.92 | | |
| **Unknown 47** | Metastatic | 10 | 5.26E+06 | Metastatic | 99.35 | | Metastatic | 99.02 | | |
| **Unknown 47** | Metastatic | 11 | 3.35E+06 | Metastatic | 99.37 | | Metastatic | 98.79 | | |
| **Unknown 48** | Metastatic | 11 | 2.88E+06 | Metastatic | 99.27 | 100.00 | Metastatic | 94.68 | 62.50 | 3.12E+05 |
| **Unknown 48** | Metastatic | 11 | 3.88E+06 | Metastatic | 99.19 | | Metastatic | 98.64 | | |
| **Unknown 48** | Metastatic | 1 | 1.11E+05 | Bad Data | - | | Bad Data | - | | |
| **Unknown 48** | Metastatic | 8 | 1.34E+06 | Metastatic | 98.83 | | Schwannoma | 94.13 | | |
| **Unknown 48** | Metastatic | 8 | 1.54E+06 | Metastatic | 99.3 | | Metastatic | 98.89 | | |
| **Unknown 48** | Metastatic | 10 | 1.87E+06 | Metastatic | 99.18 | | Metastatic | 92.11 | | |
| **Unknown 48** | Metastatic | 9 | 1.21E+06 | Metastatic | 99.31 | | Schwannoma | 97.67 | | |
| **Unknown 48** | Metastatic | 8 | 1.99E+06 | Metastatic | 99.01 | | Schwannoma | 91.96 | | |
| **Unknown 48** | Metastatic | 8 | 1.12E+06 | Metastatic | 99.29 | | Metastatic | 92.45 | | |
| **Unknown 48** | Metastatic | 1 | 2.29E+05 | Bad Data | - | | Bad Data | - | | |
| **Unknown 49** | Meningioma | 11 | 2.87E+06 | Meningioma | 99.32 | 100.00 | Meningioma | 97.82 | 100.00 | 2.85E+05 |
| **Unknown 49** | Meningioma | 9 | 2.13E+06 | Meningioma | 99.33 | | Meningioma | 99.26 | | |
| **Unknown 49** | Meningioma | 12 | 1.86E+06 | Meningioma | 99.31 | | Meningioma | 99.36 | | |
| **Unknown 49** | Meningioma | 11 | 2.35E+06 | Meningioma | 99.3 | | Meningioma | 99.31 | | |
| **Unknown 49** | Meningioma | 13 | 1.17E+06 | Meningioma | 99.23 | | Meningioma | 98.94 | | |
| **Unknown 49** | Meningioma | 7 | 1.98E+06 | Meningioma | 99.33 | | Meningioma | 99.27 | | |
| **Unknown 49** | Meningioma | 10 | 2.17E+06 | Meningioma | 99.14 | | Meningioma | 99.1 | | |
| **Unknown 49** | Meningioma | 10 | 2.38E+06 | Meningioma | 98.98 | | Meningioma | 96.45 | | |

| | | | | | | | | | | |
|---|---|---|---|---|---|---|---|---|---|---|
| **Unknown 50** | Metastatic | 10 | 3.63E+06 | Metastatic | 99.38 | 100.00 | Metastatic | 99.2 | 87.50 | 2.63E+05 |
| **Unknown 50** | Metastatic | 11 | 3.75E+06 | Metastatic | 99.15 | | Metastatic | 81.54 | | |
| **Unknown 50** | Metastatic | 10 | 2.87E+06 | Metastatic | 99.31 | | Schwannoma | 88.73 | | |
| **Unknown 50** | Metastatic | 12 | 4.35E+06 | Metastatic | 99.35 | | Metastatic | 99.34 | | |
| **Unknown 50** | Metastatic | 11 | 3.20E+06 | Metastatic | 99.27 | | Metastatic | 99.3 | | |
| **Unknown 50** | Metastatic | 10 | 5.74E+06 | Metastatic | 99.29 | | Metastatic | 99.31 | | |
| **Unknown 50** | Metastatic | 12 | 4.84E+06 | Metastatic | 98.88 | | Metastatic | 99.39 | | |
| **Unknown 50** | Metastatic | 11 | 4.13E+06 | Metastatic | 99.27 | | Metastatic | 96.17 | | |
| **Unknown 51** | Schwannoma | 9 | 1.16E+06 | Metastatic | 53.79 | 80.00 | Schwannoma | 89.34 | 100.00 | 3.30E+05 |
| **Unknown 51** | Schwannoma | 12 | 3.83E+06 | Schwannoma | 99.26 | | Schwannoma | 99.25 | | |
| **Unknown 51** | Schwannoma | 11 | 1.66E+06 | Schwannoma | 98.71 | | Schwannoma | 98.71 | | |
| **Unknown 51** | Schwannoma | 7 | 6.29E+05 | Schwannoma | 95.54 | | Schwannoma | 99.17 | | |
| **Unknown 51** | Schwannoma | 11 | 3.57E+06 | Schwannoma | 99.2 | | Schwannoma | 98.98 | | |
| **Unknown 51** | Schwannoma | 12 | 6.82E+06 | Schwannoma | 99.29 | | Schwannoma | 99.21 | | |
| **Unknown 52** | Meningioma | 11 | 6.11E+06 | Meningioma | 99.33 | 100.00 | Meningioma | 99.17 | 100.00 | 4.00E+05 |
| **Unknown 52** | Meningioma | 11 | 7.42E+06 | Meningioma | 99.3 | | Meningioma | 99.38 | | |
| **Unknown 52** | Meningioma | 11 | 5.61E+06 | Meningioma | 99.35 | | Meningioma | 99.36 | | |
| **Unknown 52** | Meningioma | 10 | 4.71E+06 | Meningioma | 99.15 | | Meningioma | 99.35 | | |
| **Unknown 53** | Metastatic | 9 | 4.24E+06 | Metastatic | 99.33 | 100.00 | Metastatic | 95.6 | 88.89 | 2.43E+05 |
| **Unknown 53** | Metastatic | 12 | 3.24E+06 | Metastatic | 99.16 | | Metastatic | 99.07 | | |
| **Unknown 53** | Metastatic | 11 | 2.25E+06 | Metastatic | 99.3 | | Metastatic | 98.32 | | |
| **Unknown 53** | Metastatic | 11 | 3.23E+06 | Metastatic | 99.36 | | Metastatic | 98.36 | | |
| **Unknown 53** | Metastatic | 10 | 3.01E+06 | Metastatic | 99.3 | | Metastatic | 91.43 | | |
| **Unknown 53** | Metastatic | 11 | 2.94E+06 | Metastatic | 99.38 | | Schwannoma | 64.41 | | |
| **Unknown 53** | Metastatic | 10 | 1.71E+06 | Metastatic | 99.36 | | Metastatic | 99.38 | | |
| **Unknown 53** | Metastatic | 11 | 2.56E+06 | Metastatic | 99.36 | | Metastatic | 99.35 | | |
| **Unknown 53** | Metastatic | 10 | 2.45E+06 | Metastatic | 99.2 | | Metastatic | 98.57 | | |
| **Unknown 54** | Metastatic | 13 | 2.25E+06 | Metastatic | 99.38 | 100.00 | Metastatic | 99.28 | 100.00 | 2.98E+05 |
| **Unknown 54** | Metastatic | 11 | 4.51E+06 | Metastatic | 99.3 | | Metastatic | 99.27 | | |
| **Unknown 54** | Metastatic | 10 | 1.38E+06 | Metastatic | 99.38 | | Metastatic | 98.68 | | |
| **Unknown 54** | Metastatic | 7 | 1.54E+06 | Metastatic | 99.39 | | Metastatic | 98.75 | | |
| **Unknown 54** | Metastatic | 12 | 2.23E+06 | Metastatic | 99.38 | | Metastatic | 99.12 | | |
| **Unknown 54** | Metastatic | 10 | 4.34E+06 | Metastatic | 99.13 | | Metastatic | 98.9 | | |
| **Unknown 54** | Metastatic | 11 | 2.49E+06 | Metastatic | 99.33 | | Metastatic | 99.35 | | |
| **Unknown 54** | Metastatic | 1 | 1.62E+05 | Bad Data | - | | Bad Data | - | | |
| **Unknown 54** | Metastatic | 10 | 5.21E+06 | Metastatic | 99.35 | | Metastatic | 99.18 | | |
| **Unknown 55** | Metastatic | 12 | 4.85E+06 | Metastatic | 98.99 | 100.00 | Schwannoma | 99.09 | 91.67 | 2.18E+05 |
| **Unknown 55** | Metastatic | 12 | 5.09E+06 | Metastatic | 99.21 | | Metastatic | 99.17 | | |
| **Unknown 55** | Metastatic | 10 | 4.60E+06 | Metastatic | 99.22 | | Metastatic | 99.1 | | |
| **Unknown 55** | Metastatic | 10 | 3.76E+06 | Metastatic | 98.94 | | Metastatic | 99.2 | | |
| **Unknown 55** | Metastatic | 10 | 2.24E+06 | Unclassifiable | - | | Metastatic | 99 | | |
| **Unknown 55** | Metastatic | 11 | 5.22E+06 | Metastatic | 99.28 | | Metastatic | 99.33 | | |
| **Unknown 55** | Metastatic | 10 | 1.66E+06 | Unclassifiable | - | | Metastatic | 99 | | |
| **Unknown 55** | Metastatic | 10 | 4.66E+06 | Metastatic | 99.1 | | Metastatic | 99.37 | | |
| **Unknown 55** | Metastatic | 10 | 5.31E+06 | Unclassifiable | - | | Metastatic | 98.85 | | |
| **Unknown 55** | Metastatic | 11 | 6.31E+06 | Metastatic | 99.25 | | Metastatic | 99.2 | | |
| **Unknown 55** | Metastatic | 10 | 2.03E+06 | Metastatic | 99.2 | | Metastatic | 99.31 | | |
| **Unknown 55** | Metastatic | 10 | 2.39E+06 | Metastatic | 99.31 | | Metastatic | 99.26 | | |
| **Unknown 56** | Schwannoma | 1 | 2.31E+05 | Bad Data | - | 100.00 | Bad Data | - | 100.00 | 3.12E+05 |
| **Unknown 56** | Schwannoma | 11 | 4.65E+06 | Schwannoma | 99.09 | | Schwannoma | 99.24 | | |
| **Unknown 56** | Schwannoma | 11 | 4.71E+06 | Schwannoma | 99.14 | | Schwannoma | 99.31 | | |
| **Unknown 56** | Schwannoma | 11 | 6.76E+06 | Schwannoma | 99.27 | | Schwannoma | 99.32 | | |
| **Unknown 56** | Schwannoma | 11 | 8.57E+06 | Schwannoma | 99 | | Schwannoma | 99.11 | | |
| **Unknown 56** | Schwannoma | 13 | 2.76E+06 | Schwannoma | 99.37 | | Schwannoma | 98.9 | | |
| **Unknown 56** | Schwannoma | 10 | 5.18E+06 | Schwannoma | 99.37 | | Schwannoma | 98.92 | | |
| **Unknown 56** | Schwannoma | 13 | 3.27E+06 | Schwannoma | 99.32 | | Schwannoma | 99.16 | | |
| **Unknown 56** | Schwannoma | 10 | 6.18E+06 | Schwannoma | 99.33 | | Schwannoma | 99.05 | | |
| **Unknown 56** | Schwannoma | 11 | 7.98E+06 | Schwannoma | 99.28 | | Schwannoma | 96.67 | | |
| **Unknown 56** | Schwannoma | 12 | 9.01E+06 | Schwannoma | 99.32 | | Schwannoma | 99.09 | | |
| **Unknown 57** | Meningioma | 10 | 2.39E+06 | Meningioma | 99.19 | 100.00 | Meningioma | 98.43 | 100.00 | 3.11E+05 |
| **Unknown 57** | Meningioma | 11 | 5.59E+06 | Meningioma | 99.22 | | Meningioma | 98.22 | | |
| **Unknown 57** | Meningioma | 11 | 3.55E+06 | Meningioma | 99.27 | | Meningioma | 99.14 | | |
| **Unknown 57** | Meningioma | 10 | 4.19E+06 | Meningioma | 99.37 | | Meningioma | 98.47 | | |
| **Unknown 57** | Meningioma | 11 | 2.84E+06 | Meningioma | 99.25 | | Meningioma | 98.91 | | |
| **Unknown 57** | Meningioma | 10 | 2.99E+06 | Meningioma | 99.15 | | Meningioma | 99.3 | | |
| **Unknown 58** | Meningioma | 11 | 4.66E+06 | Meningioma | 99.34 | 100.00 | Meningioma | 97.94 | 90.91 | 2.28E+05 |
| **Unknown 58** | Meningioma | 10 | 3.26E+06 | Meningioma | 99.29 | | Meningioma | 92.42 | | |
| **Unknown 58** | Meningioma | 11 | 3.72E+06 | Meningioma | 99.32 | | Meningioma | 95.81 | | |
| **Unknown 58** | Meningioma | 11 | 3.76E+06 | Meningioma | 99.37 | | Meningioma | 99.15 | | |
| **Unknown 58** | Meningioma | 11 | 2.44E+06 | Meningioma | 99.35 | | Meningioma | 97.92 | | |
| **Unknown 58** | Meningioma | 9 | 3.01E+06 | Meningioma | 99.38 | | Meningioma | 95.74 | | |
| **Unknown 58** | Meningioma | 11 | 1.82E+06 | Meningioma | 99.36 | | Meningioma | 97.9 | | |
| **Unknown 58** | Meningioma | 11 | 2.47E+06 | Meningioma | 99.25 | | Meningioma | 99.18 | | |
| **Unknown 58** | Meningioma | 10 | 2.40E+06 | Meningioma | 99.38 | | Meningioma | 98.74 | | |
| **Unknown 58** | Meningioma | 9 | 1.30E+06 | Meningioma | 99.33 | | Schwannoma | 70.21 | | |
| **Unknown 58** | Meningioma | 11 | 2.00E+06 | Meningioma | 99.35 | | Meningioma | 99.22 | | |
| **Unknown 59** | Metastatic | 9 | 1.35E+06 | Metastatic | 99.32 | 100.00 | Metastatic | 95.63 | 83.33 | 3.10E+05 |
| **Unknown 59** | Metastatic | 10 | 1.29E+06 | Metastatic | 99.35 | | Metastatic | 99.18 | | |
| **Unknown 59** | Metastatic | 9 | 1.01E+06 | Metastatic | 98.51 | | Metastatic | 94.13 | | |
| **Unknown 59** | Metastatic | 6 | 1.20E+06 | Metastatic | 99.19 | | Metastatic | 93.59 | | |
| **Unknown 59** | Metastatic | 7 | 1.11E+06 | Metastatic | 99.34 | | Metastatic | 99.3 | | |
| **Unknown 59** | Metastatic | 8 | 1.86E+06 | Metastatic | 99.32 | | Metastatic | 99.11 | | |
| **Unknown 59** | Metastatic | 8 | 2.09E+06 | Metastatic | 98.73 | | Schwannoma | 62.83 | | |
| **Unknown 60** | Meningioma | 12 | 6.08E+06 | Meningioma | 99.23 | 100.00 | Meningioma | 99.19 | 100.00 | 4.14E+05 |
| **Unknown 60** | Meningioma | 10 | 2.78E+06 | Meningioma | 99.26 | | Meningioma | 99.26 | | |
| **Unknown 60** | Meningioma | 11 | 5.63E+06 | Meningioma | 99.37 | | Meningioma | 99.37 | | |
| **Unknown 60** | Meningioma | 10 | 2.05E+06 | Meningioma | 99.34 | | Meningioma | 99.02 | | |
| **Unknown 60** | Meningioma | 1 | 2.13E+05 | Bad Data | - | | Bad Data | - | | |
| **Unknown 60** | Meningioma | 1 | 1.09E+05 | Bad Data | - | | Bad Data | - | | |

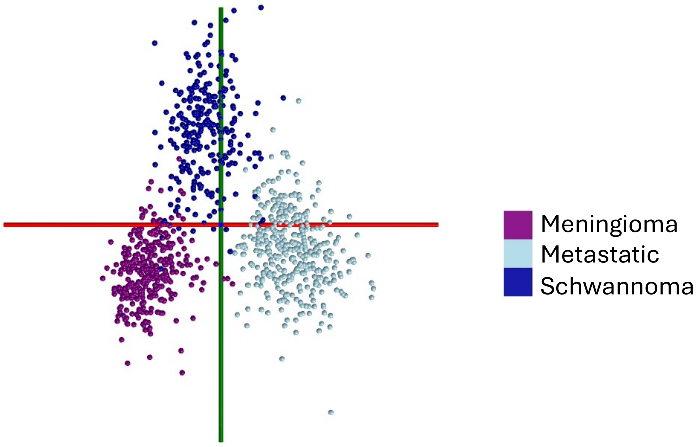

**Table S4. The results of blind sample predictions using the Fig. 2A PCA-LDA model.** Here, are showing the expected (from pathology) and observed (from PIRL-MS modeling, cluster overlap and Mahalanobis distance mapping[1] of the spectral profiles) annotations alongside signal duration (in seconds), signal strength (as in TIC averaged over said duration) as well as probability values for each prediction made from Mahalanobis distance mapping[1]. In addition, we have reported a rate of spatially invariant correct predictions for each independent specimen analyzed. The overall rate of spatially concordant correct prediction was 92.34%. This calculation excluded bad data and unclassified data based on which no clinical decision is made, only affecting the duty cycle. In this table we have provided the results from n=487 sampling events from n=60 independent blind samples (n=20 per classes of meningioma, schwannoma and metastatic tumours) across full *m/z* 100-1,000 Da range (for Table 1 sensitivity and specificity) as well as sparse analysis using the ions reported in Table 2. There are n=16 unclassifiable sampling events out of the n=487 attempted and n=24 sampling attempts resulted in bad data defined as possessing signal duration of less than (or equal to) 3 seconds. Therefore, the total of classifiable events (n=447) resulted in a duty cycle of ~92% with 8% of the sampling attempts not resulting in useful data. In addition, n=25 events (underlined) possessed Mahalanobis class prediction values of less than 95% confidence, resulting in a dataset of n=422 for those most rigorously predicted with >95% confidence in probability for PIRL-MS predictions. We are also showing the sparse predictions (feature-based prediction results of same specimens using the 41-ion biomarker array shown in Table 2). These results were subsequently used in calculations of feature-based sensitivity and specificity values presented in Table 3 only utilizing the said identified n=41 ions. Sampling events with less than 95% confidence in prediction values from Mahalanobis distance mapping[1] are also underlined (n=56). The sparse dataset possessed similar spatially invariant prediction rate of ~88.91% (compared to 92.34% for full length model). This table also shows spectral similarity metrics calculated from the Pearson correlation coefficient for each unknown specimen used for blind sample classifications compared to those included in the model. For this test, only the specimens with more than n=2 sampling events were included in the analysis. The average (±1 standard deviation) value for this coefficient for correctly classified meningioma, schwannoma and metastatic unknowns were $(3.20 \pm 0.60) \cdot 10^5$, $(3.05 \pm 0.40) \cdot 10^5$, $(2.85 \pm 0.70) \cdot 10^5$, respectively and used as the baseline. Additionally, for select misclassified cases wherein we have performed Sox10 immunohistochemistry, the IHC status is also listed in the final column. For clarity, we are also presenting the PCA-LDA scores plot of the sparse variant of Fig. 2A used for sparse analysis (feature-based classifications) provided in this table as well. Compared to Fig. 2A, slightly closer grouping of meningioma, schwannoma and metastatic tumour clusters were seen but this model did not drastically underperform the Fig. 2A in cross-validation assessment possessing 20% leave out and full-group leave out cross-validation accuracies of 96.55% and 93.20%, respectively (confusion matrix not shown for brevity). Comparing these performance values to Table S2 results of 98.84% and 96.27% accuracies suggest that the identified 41 ions (Table 2) do sufficiently capture the information needed to distinguish meningioma, schwannoma and metastatic tumours with 10-second PIRL-MS analysis.

| Top 100 Binned *m/z* | PIRL-MS Nominal *m/z* |
|---|---|
| 126.95 | 126.9019 |
| 127.05 | 127.0381 |
| 135.05 | 135.0517 |
| 142.05 | 142.0392 |
| 143.05 | 143.0296 |
| 164.05 | 164.0413 |
| 215.05 | 215.0872 |
| 271.25 | 271.2226 |
| 277.05 | 277.0662 |
| 329.25 | 329.2461 |
| 331.25 | 331.2614 |
| 347.15 | 347.1445 |
| 355.35 | 355.3198 |
| 365.35 | 365.3411 |
| 375.15 | 375.1805 |
| 377.25 | 377.2494 |
| 391.25 | 391.2248 |
| 415.25 | 415.2253 |
| 439.25 | 439.2256 |
| 441.25 | 441.2233 |
| 463.35 | 463.2226 |
| 570.45 | 570.4614 |
| 574.55 | 574.4844 |
| 588.45 | 588.4729 |
| 598.45 | 598.4959 |
| 598.55 | 598.5139 |
| 599.55 | 599.4998 |
| 603.45 | 603.4675 |
| 626.55 | 626.5344 |
| 627.45 | 627.4737 |
| 628.45 | 628.4791 |
| 629.45 | 629.4892 |
| 630.45 | 630.4879 |
| 631.45 | 631.4893 |
| 637.55 | 637.5255 |
| 642.55 | 642.5573 |
| 654.55 | 654.5626 |
| 655.55 | 655.5022 |
| 656.55 | 656.5726 |
| 658.55 | 658.5789 |
| 659.45 | 659.4606 |
| 659.55 | 659.5202 |
| 660.55 | 660.5223 |
| 661.45 | 661.4802 |
| 668.55 | 668.5808 |
| 672.55 | 672.5754 |
| 673.45 | 673.4678 |
| 677.45 | 677.4861 |
| 679.55 | 679.5051 |
| 685.45 | 685.4817 |
| 686.55 | 686.5285 |
| 686.65 | 686.6032 |
| 687.45 | 687.4952 |
| 687.65 | 687.6076 |
| 689.65 | 689.6230 |
| 691.45 | 691.4921 |
| 693.55 | 693.5770 |
| 694.55 | 694.5801 |
| 696.65 | 696.6058 |
| 698.55 | 698.5864 |
| 698.65 | 698.6232 |
| 699.55 | 699.5968 |
| 703.45 | 703.4164 |
| 703.55 | 703.5101 |
| 704.55 | 704.5112 |
| 705.55 | 705.5159 |
| 708.55 | 708.5795 |
| 709.45 | 709.4800 |
| 715.45 | 715.4051 |
| 717.55 | 715.5127 |
| 718.55 | 718.5203 |
| 719.45 | 719.4260 |
| 719.55 | 719.5198 |
| 721.55 | 721.5138 |
| 722.55 | 722.5268 |
| 723.45 | 723.5016 |
| 733.55 | 733.5588 |
| 734.55 | 734.5410 |
| 735.55 | 735.5809 |
| 736.65 | 736.6438 |
| 744.55 | 744.5493 |
| 745.55 | 745.5457 |
| 746.55 | 746.5496 |
| 747.45 | 747.4841 |
| 750.55 | 750.5397 |
| 763.55 | 763.5667 |
| 764.55 | 764.5359 |
| 767.55 | 767.5276 |
| 768.55 | 768.5399 |
| 770.55 | 770.5630 |
| 777.65 | 777.6687 |
| 794.55 | 794.5651 |
| 816.55 | 816.5457 |
| 818.65 | 818.6290 |
| 826.65 | 826.6313 |
| 827.65 | 827.6282 |
| 830.65 | 830.6498 |
| 844.65 | 844.6404 |
| 846.65 | 846.6531 |
| 860.65 | 860.6646 |

**Table S5. The top 100 tumour type identifying *m/z* values.** In this table we are listing the top 100 *m/z* lead features from analysis of the so-called 'loading plots' of Fig. 2A PCA-LDA model (Fig. S2). This list was comprised of 50 most positively and 50 most negatively discriminating features across both linear discriminant dimensions shown in Fig. 2A (25 features each). This list was used to guide the targeted identification of as many of these ions as possible by UPLC-MS/MS analysis, leading to the discovery of Table 2 biomarker ions for spinal tumour classification with 10-second PIRL-MS. To generate this list, the ions' contributions were ranked independently across each LD (top 25 positively influencing and top 25 negatively influencing based on their rank order contribution (to statistical discrimination) values). Here, the results across each LD axis were ranked independently to increase the comprehensives of the lead *m/z* subjected to targeted identification by UPLC-MS/MS. In addition to ion IDs reported as spectral bins (at 0.1 Da) based on which all PCA-LDA multivariate modeling in this work has been performed, we have also included the nominal, lock-mass corrected PIRL-MS *m/z* values for each of the identified most discriminating ions listed in this table.

**20% Leave Out**

| | Data point groups | | | | | Correct Classification Rate | |
|---|---|---|---|---|---|---|---|
| | PIRL-MS spectra data points | Correctly classified data points | Misclassified data points | Unclassifiable data points | Classifiable data points | Per classifiable data points | Per all (classifiable and unclassifiable) data points |
| **Total** | 2148 | 2060 | 26 | 62 | **2086** | **98.75%** | **95.90%** |

| | Meningioma | Myxopapillary Ependymoma | Neurofibroma | Paranganglioma | Schwannoma | Solitary Fibrous Tumour | Unclassifiable | Total |
|---|---|---|---|---|---|---|---|---|
| Meningioma | 923 | 0 | 0 | 0 | 10 | 0 | 16 | 949 |
| Myxopapillary Ependymoma | 0 | 134 | 1 | 0 | 0 | 0 | 6 | 141 |
| Neurofibroma | 0 | 0 | 160 | 0 | 0 | 0 | 6 | 166 |
| Paranganglioma | 0 | 0 | 0 | 88 | 0 | 0 | 6 | 94 |
| Schwannoma | 15 | 0 | 0 | 0 | 656 | 0 | 24 | 695 |
| Solitary Fibrous Tumour | 0 | 0 | 0 | 0 | 0 | 99 | 4 | 103 |
| **Total** | **938** | **134** | **161** | **88** | **666** | **99** | **62** | **2148** |

**Full group leave out**

| | Data point groups | | | | | Correct Classification Rate | |
|---|---|---|---|---|---|---|---|
| Group | PIRL-MS spectra data points | Correctly classified data points | Misclassified data points | Unclassifiable data points | Classifiable data points | Per classifiable data points | Per all (classifiable and unclassifiable) data points |
| 1 | 390 | 367 | 4 | 19 | 371 | 98.92% | 94.10% |
| 2 | 460 | 411 | 6 | 43 | 417 | 98.56% | 89.35% |
| 3 | 450 | 407 | 25 | 18 | 432 | 94.21% | 90.44% |
| 4 | 422 | 365 | 15 | 42 | 380 | 96.05% | 86.49% |
| 5 | 426 | 367 | 22 | 37 | 389 | 94.34% | 86.15% |
| **Total** | **2148** | **1917** | **72** | **159** | **1989** | **96.38%** | **89.25%** |

| | Meningioma | Myxopapillary Ependymoma | Neurofibroma | Paranganglioma | Schwannoma | Solitary Fibrous Tumour | Unclassifiable | Total |
|---|---|---|---|---|---|---|---|---|
| Meningioma | 863 | 0 | 0 | 0 | 38 | 0 | 48 | 949 |
| Myxopapillary Ependymoma | 0 | 109 | 3 | 0 | 0 | 0 | 29 | 141 |
| Neurofibroma | 0 | 0 | 150 | 0 | 0 | 0 | 16 | 166 |
| Paranganglioma | 0 | 0 | 0 | 79 | 0 | 0 | 15 | 94 |
| Schwannoma | 29 | 0 | 2 | 0 | 623 | 0 | 41 | 695 |
| Solitary Fibrous Tumour | 0 | 0 | 0 | 0 | 0 | 93 | 10 | 103 |
| **Total** | **892** | **109** | **155** | **79** | **661** | **93** | **159** | **2148** |

**Table S6. Cross-validation statistics of Fig. 3 PCA-LDA model containing additional spinal tumour types.** The cross-validation statistics across both 20% and full group leave-out tests for intradural extramedullary spinal cancer types are calculated as described in the experimental section. The results suggest strong differentiation between these tumour types is possible with 10-second PIRL-MS profiling, high cross-validation accuracies have been obtained. Except for a few schwannoma and meningioma 10-second spectra that misclassified with one another there were no significant other model failures noted in the intradural extramedullary group. The results in this table are based on $n$=2,148, 10-second PIRL-MS spectra from $n$=257 specimens with the following breakdowns: meningioma ($n$=97), schwannoma ($n$=106), myxopapillary ependymoma ($n$=18), neurofibroma ($n$=18), paraganglioma ($n$=9), solitary fibrous tumours also known as hemangiopericytoma ($n$=9)

**20% leave out**

| | Data point groups | | | | | Correct Classification Rate | |
|---|---|---|---|---|---|---|---|
| | PIRL-MS spectra data points | Correctly classified data points | Misclassified data points | Unclassifiable data points | Classifiable data points | Per classifiable data points | Per all (classifiable and unclassifiable) data points |
| **Total** | 2148 | 1987 | 150 | 11 | **2137** | **92.98%** | **92.50%** |

| | Meningioma | Myxopapillary Ependymoma | Neurofibroma | Paraganglioma | Schwannoma | Solitary Fibrous Tumour | Unclassifiable | Total |
|---|---|---|---|---|---|---|---|---|
| Meningioma | 906 | 0 | 5 | 9 | 22 | 4 | 3 | **949** |
| Myxopapillary Ependymc | 0 | 122 | 16 | 2 | 0 | 0 | 1 | **141** |
| Neurofibroma | 2 | 0 | 156 | 0 | 4 | 1 | 3 | **166** |
| Paranganglioma | 1 | 0 | 1 | 90 | 1 | 0 | 1 | **94** |
| Schwannoma | 36 | 0 | 29 | 7 | 618 | 2 | 3 | **695** |
| Solitary Fibrous Tumour | 0 | 0 | 3 | 5 | 0 | 95 | 0 | **103** |
| **Total** | **945** | **122** | **210** | **113** | **645** | **102** | **11** | **2148** |

**Full group leave out**

| | Data point groups | | | | | Correct Classification Rate | |
|---|---|---|---|---|---|---|---|
| Group | PIRL-MS spectra data points | Correctly classified data points | Misclassified data points | Unclassifiable data points | Classifiable data points | Per classifiable data points | Per all (classifiable and unclassifiable) data points |
| 1 | 390 | 368 | 20 | 2 | 388 | 94.85% | 94.36% |
| 2 | 460 | 414 | 42 | 4 | 456 | 90.79% | 90.00% |
| 3 | 450 | 410 | 39 | 1 | 449 | 91.31% | 91.11% |
| 4 | 422 | 370 | 45 | 7 | 415 | 89.16% | 87.68% |
| 5 | 426 | 377 | 47 | 2 | 424 | 88.92% | 88.50% |
| **Total** | **2148** | **1939** | **193** | **16** | **2132** | **90.95%** | **90.27%** |

| | Meningioma | Myxopapillary Ependymoma | Neurofibroma | Paranganglioma | Schwannoma | Solitary Fibrous Tumour | Unclassifiable | Total |
|---|---|---|---|---|---|---|---|---|
| Meningioma | 885 | 0 | 4 | 15 | 36 | 4 | 5 | **949** |
| Myxopapillary Ependymc | 0 | 117 | 19 | 2 | 0 | 0 | 3 | **141** |
| Neurofibroma | 3 | 0 | 155 | 0 | 4 | 1 | 3 | **166** |
| Paranganglioma | 6 | 0 | 2 | 83 | 0 | 2 | 1 | **94** |
| Schwannoma | 38 | 0 | 33 | 9 | 610 | 1 | 4 | **695** |
| Solitary Fibrous Tumour | 0 | 0 | 4 | 10 | 0 | 89 | 0 | **103** |
| **Total** | **932** | **117** | **217** | **119** | **650** | **97** | **16** | **2148** |

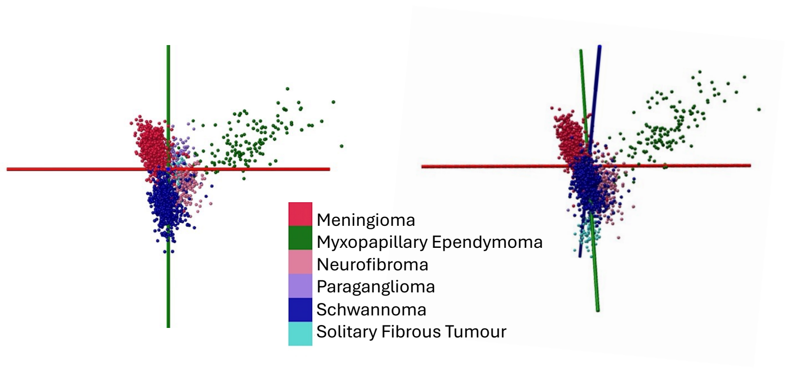


**Table S7. Cross-validation statistics of Fig. 3 PCA-LDA model using Table 2 biomarker ion list.** This table summarizes the cross-validation (20% leave-out) statistics of Fig. 3 dataset using sparse PCA-LDA analysis only utilizing the Table 2 biomarker ions. The statistics are provided alongside confusion matrices from both 20% (sampling event based) and full group leave-out (specimen based) 5-fold cross-validations and are not drastically lower than those presented in Table S6 for full *m/z* feature utilization. We deliberately used the Table 2 features extracted for schwannoma, meningioma and metastatic cancer differentiation in this assessment to verify the robustness of its use in the presence of additional neurofibroma, myxopapillary ependymoma, solitary fibrous tumour and paraganglioma. As shown, presence of additional data from tumours not seen by the array/Fig. 2A model did not have a drastic effect on the ability of the said molecular array to mediate the distinction between meningioma and schwannoma. However, as slight misclassifications between the added neurofibroma, paraganglioma and solitary fibrous tumours were seen, we must revisit a new list of markers to enable their differentiation. For clarity, the PCA-LDA scores plot of this sparse model is also shown (two angles).

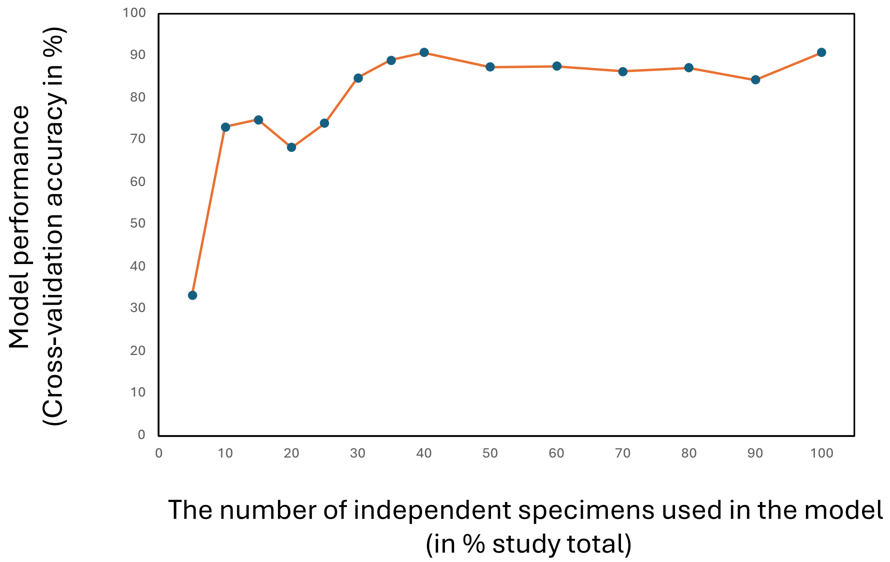


**Figure S1. The learning curve of the PCA-LDA model of Fig. 2A.** Here, we have plotted the cross-validation (via a modified 20% leave-out wherein we have preselected at random only one sampling event per specimen) statistics for a series of PCA-LDA models that were made at 10% total data (i.e. independent specimen) usage. The Fig. 2A model is created from n=122 independent patient specimens (n=39 meningiomas, n=41 schwannoma and n=42 metastatic tumours) producing n=959 sampling events. The cross-validation accuracy reaches a plateau at ~50% total independent specimen used, which brings our cohort size within the realm of what is needed to reach statistical significance. Having said this, however, large(r) data cohorts may be needed to assess the mass spectral signal variance across the population level. Nevertheless, we believe at 100% data usage we are capturing a reasonable level of this noise for said model to be relatively generalizable with safe assumptions. The plateau reached at beyond 50% data usage continues to hold steady (within 5% variance) up to top 100% data usage. Perhaps, by way of extrapolating this trend, including additional specimens beyond the 100% may not drastically enhance the statistical performance. In keeping with previous stipulations[2], however, at <20% data usage (very few independent specimens) likely 'overfitting' in the cross-validation may result in uninterpretable data. To ensure, interpretable results however as published by our group recently [2] we utilized the following n number of PCA components for each model at data usage % of 5% (n=2), 10% (n=3), 15% (n=4), 20% (n=4), 25% (n=5), 30% (n=6), 35% (n=8), 40% (n=8), 50% (n=11), 60% (n=13), 70% (n=16), 80% (n=19), 90% (n=21) and 100% (n=25). For this learning curve we used one representative mass spectrum for each independent specimen to avoid bias in cross-validation accuracies.

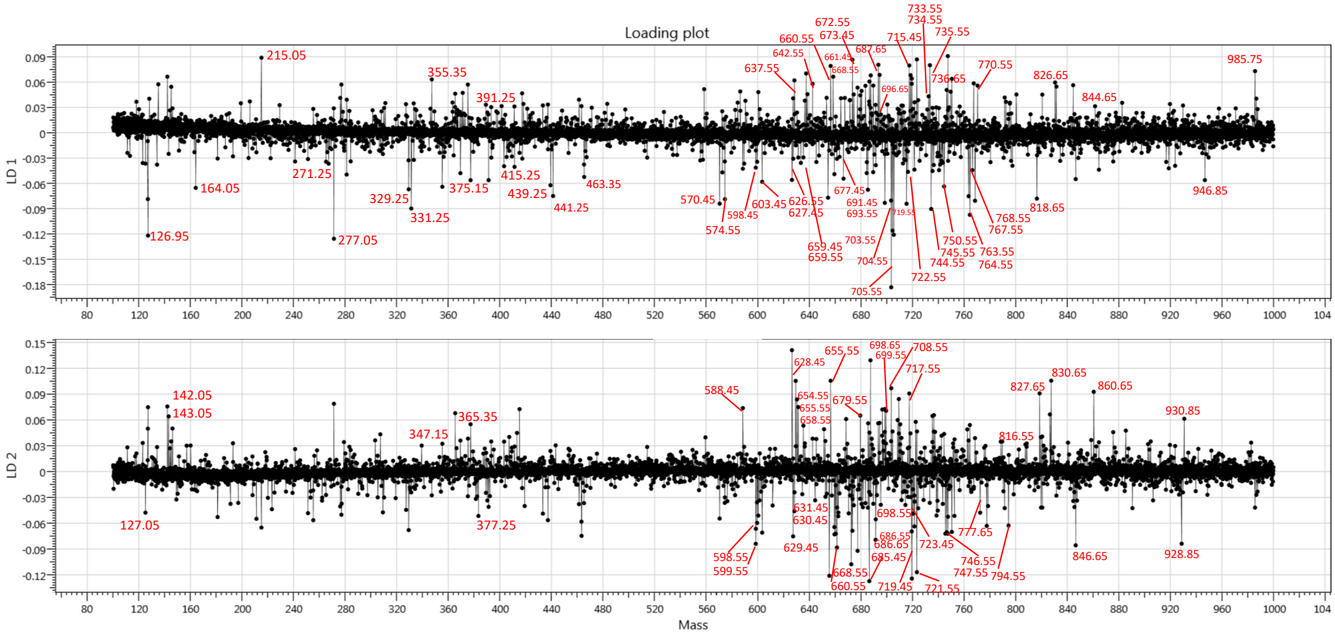


**Figure S2. The loading plot analysis of Fig. 2A PCA-LDA scores plot for major spinal tumour differentiation with 10-second PIRL-MS profiling.** The loading plots were generated with AMX [3] across both linear dimensions of LD1 and LD2. The top 100 *m/z* values listed in Table S5 used as leads for targeted identification with UPLC-MS/MS are highlighted in red. To generate these leads, we sorted the PIRL-MS mass spectral bins (0.1 Da) across both positively and negatively contributing directions (along both linear discriminant LD1 and LD2 axes), thus selecting top 25 features in each permutation of the direction of contribution and LD (1 or 2) axis. For clarity of the presentation, we have taken the liberty to plot the ions that overlapped between LD1 and LD2 from the list of top 100 on the LD component where space was more favourable, permitting legibility. As such, for display purposes only more or less than 25 ions per LD axis per direction may have been shown.

*Specimens and Histology*

The table below provides a summary of the histopathologic evaluation of the specimens used in this study alongside notes by a practicing neuropathologist who performed evaluations of post PIRL-MS hematoxylin and eosin (H&E) slides as well as clinical records to provide final diagnosis. The PIRL-MS operator documented the spatial distribution (or pattern) of all sampling events performed for each specimen. This pattern is largely visible in the post PIRL-MS H&E images (available for each specimen) and allows us to link the PIRL-MS data of each sampling event to tissue histologic heterogeneity near sampling site. The ability to trace histologic heterogeneity to problematic sampling event data has proven useful in rationalizing misclassified sampling events in a previous study on pediatric brain cancers[4]. In our work here, however, most of the problematic specimens had all their sampling events misclassified (See Table S4 results). As such, tracing the sampling patterns to rationalize model's behaviour in terms of local (near the site of sampling) histological heterogeneity was not deemed to be informative and thus not pursued. As discussed (see below), the problematic specimens often possessed overall PIRL-MS signatures that matched other tumour classes with all of their sampling events being misclassified. Where appropriate, we have performed additional immunohistochemistry (IHC) analysis to confirm the diagnosis (discussed in the text with results reported in Table S4). In this table 'expected class' refers to the specimen based on histologic ground truth used in the model (for PCA-LDA) and for scoring the PIRL-MS predictions (in blind sample validation).

| Specimen ID | Expected Class | Histologic Class | WHO Grade | Metastatic Origin | Pathology Notes |
|---|---|---|---|---|---|
| *PCA-LDA Model* | | | | | |
| ABT SMH-104 | Meningioma | Meningioma | I | n/a | unremarkable |
| ABT SMH-105 | Meningioma | Psammomatous Meningioma | I | n/a | unremarkable |
| ABT SMH-107 | Meningioma | Meningothelial Meningioma | I | n/a | unremarkable |
| ABT SMH-108 | Meningioma | Meningioma | I | n/a | unremarkable |
| ABT SMH-109 | Meningioma | Meningioma | I | n/a | unremarkable |
| ABT SMH-110 | Meningioma | Fibrous Meningioma | I | n/a | unremarkable |
| ABT SMH-111 | Meningioma | Meningioma | I | n/a | unremarkable |
| ABT SMH-112 | Meningioma | Atypical Meningioma | II | n/a | unremarkable |
| ABT SMH-113 | Meningioma | Meningothelial Meningioma | I | n/a | unremarkable |
| ABT SMH-154 | Meningioma | Meningioma | I | n/a | unremarkable |
| ABT SMH-157 | Meningioma | Meningioma | I | n/a | unremarkable |
| ABT SMH-158 | Meningioma | Atypical meningioma | II | n/a | with necrosis |
| ABT SMH-164 | Meningioma | Meningioma | I | n/a | unremarkable |
| ABT SMH-166 | Meningioma | Meningioma | I | n/a | unremarkable |
| ABT SMH-167 | Meningioma | Atypical meningioma | II | n/a | unremarkable |
| ABT SMH-168 | Meningioma | Meningioma, not otherwise characterized | I | n/a | unremarkable |
| ABT SMH-173 | Meningioma | Atypical meningioma | II | n/a | Hylanized tissue present |
| ABT SMH-174 | Meningioma | Atypical meningioma | II | n/a | unremarkable |
| ABT SMH-175 | Meningioma | Atypical meningioma | II | n/a | unremarkable |
| ABT SMH-176 | Meningioma | Atypical meningioma | II | n/a | unremarkable |
| ABT SMH-184 | Meningioma | Atypical meningioma | II | n/a | unremarkable |
| ABT SMH-188 | Meningioma | Meningioma, fibrous | I | n/a | Intersecting bundles; familial syndrome |
| ABT SMH-189 | Meningioma | Meningioma, fibrous | I | n/a | unremarkable |
| ABT SMH-190 | Meningioma | Meningioma, transitional | I | n/a | unremarkable |
| ABT SMH-191 | Meningioma | Atypical meningioma | II | n/a | Mixture of meningioma and blood |
| ABT SMH-59 | Meningioma | Atypical meningioma | II | n/a | unremarkable |
| ABT SMH-60 | Meningioma | Transitional Meningioma | I | n/a | unremarkable |
| ABT SMH-61 | Meningioma | Meningothelial Meningioma | I | n/a | unremarkable |
| ABT SMH-62 | Meningioma | Meningioma | I | n/a | unremarkable |
| ABT SMH-64 | Meningioma | Meningioma | I | n/a | unremarkable |
| ABT SMH-65 | Meningioma | Atypical meningioma | II | n/a | unremarkable |
| ABT SMH-66 | Meningioma | Meningioma | I | n/a | unremarkable |
| ABT SMH-67 | Meningioma | Meningioma | I | n/a | unremarkable |
| ABT SMH-68 | Meningioma | Atypical meningioma | II | n/a | unremarkable |
| ABT SMH-69 | Meningioma | Meningioma | I | n/a | unremarkable |
| ABT SMH-70 | Meningioma | Meningioma | I | n/a | patient records used |
| ABT SMH-71 | Meningioma | Atypical meningioma | II | n/a | unremarkable |
| ABT SMH-72 | Meningioma | Meningioma | I | n/a | unremarkable |
| ABT SMH-73 | Meningioma | Transitional Meningioma | I | n/a | unremarkable |
| ABT SMH-19 | Metastatic | n/a | n/a | Breast | unremarkable |
| ABT SMH-195 | Metastatic | n/a | n/a | Lung | unremarkable |
| ABT SMH-197 | Metastatic | n/a | n/a | Lung | unremarkable |
| ABT SMH-205 | Metastatic | n/a | n/a | Lung | unremarkable |
| ABT SMH-207 | Metastatic | n/a | n/a | Lung | unremarkable |
| ABT SMH-211 | Metastatic | n/a | n/a | Lung | unremarkable |
| ABT SMH-212 | Metastatic | n/a | n/a | Lung | unremarkable |
| ABT SMH-218 | Metastatic | n/a | n/a | Lung | unremarkable |
| ABT SMH-220 | Metastatic | n/a | n/a | Lung | unremarkable |
| ABT SMH-221 | Metastatic | n/a | n/a | Lung | unremarkable |
| ABT SMH-222 | Metastatic | n/a | n/a | Lung | unremarkable |
| ABT SMH-229 | Metastatic | n/a | n/a | Lung | unremarkable |
| ABT SMH-23 | Metastatic | n/a | n/a | Esophagus | unremarkable |

| | | | | | |
|---|---|---|---|---|---|
| ABT SMH-230 | Metastatic | n/a | n/a | Lung | unremarkable |
| ABT SMH-232 | Metastatic | n/a | n/a | Lung | unremarkable |
| ABT SMH-235 | Metastatic | n/a | n/a | Lung | unremarkable |
| ABT SMH-239 | Metastatic | n/a | n/a | Lung | unremarkable |
| ABT SMH-25 | Metastatic | n/a | n/a | Renal | unremarkable |
| ABT SMH-253 | Metastatic | n/a | n/a | Breast | unremarkable |
| ABT SMH-255 | Metastatic | n/a | n/a | Breast | unremarkable |
| ABT SMH-257 | Metastatic | n/a | n/a | Breast | unremarkable |
| ABT SMH-263 | Metastatic | n/a | n/a | Breast | unremarkable |
| ABT SMH-266 | Metastatic | n/a | n/a | Breast | unremarkable |
| ABT SMH-268 | Metastatic | n/a | n/a | Breast | unremarkable |
| ABT SMH-270 | Metastatic | n/a | n/a | Colon | unremarkable |
| ABT SMH-275 | Metastatic | n/a | n/a | Colon | unremarkable |
| ABT SMH-277 | Metastatic | n/a | n/a | Colon | unremarkable |
| ABT SMH-28 | Metastatic | n/a | n/a | Renal | unremarkable |
| ABT SMH-375 | Metastatic | n/a | n/a | esophagus | unremarkable |
| ABT SMH-380 | Metastatic | n/a | n/a | esophagus | unremarkable |
| ABT SMH-385 | Metastatic | n/a | n/a | biliary tract | unremarkable |
| ABT SMH-387 | Metastatic | n/a | n/a | prostate | unremarkable |
| ABT SMH-400 | Metastatic | n/a | n/a | uterus (fallopian tubes) | unremarkable |
| ABT SMH-402 | Metastatic | n/a | n/a | ovary | unremarkable |
| ABT SMH-404 | Metastatic | n/a | n/a | unsure | unremarkable |
| ABT SMH-407 | Metastatic | n/a | n/a | unsure | unremarkable |
| ABT SMH-408 | Metastatic | n/a | n/a | unsure | unremarkable |
| ABT SMH-477 | Metastatic | n/a | n/a | unsure | unremarkable |
| ABT SMH-77 | Metastatic | n/a | n/a | not provided | unremarkable |
| ABT SMH-78 | Metastatic | n/a | n/a | not provided | unremarkable |
| ABT SMH-81 | Metastatic | n/a | n/a | not provided | unremarkable |
| ABT SMH-83 | Metastatic | n/a | n/a | not provided | unremarkable |
| ABT SMH-118 | Schwannoma | Schwannoma | I | n/a | unremarkable |
| ABT SMH-121 | Schwannoma | | I | n/a | unremarkable |
| ABT SMH-122 | Schwannoma | Schwannoma | I | n/a | unremarkable |
| ABT SMH-123 | Schwannoma | | I | n/a | Schwannoma, abundant hemosiderin |
| ABT SMH-125 | Schwannoma | Schwannoma | I | n/a | unremarkable |
| ABT SMH-126B | Schwannoma | | I | n/a | unremarkable |
| ABT SMH-128 | Schwannoma | Schwannoma | I | n/a | unremarkable |
| ABT SMH-129B | Schwannoma | | I | n/a | unremarkable |
| ABT SMH-131 | Schwannoma | Schwannoma | I | n/a | unremarkable |
| ABT SMH-131B | Schwannoma | | I | n/a | unremarkable |
| ABT SMH-133B | Schwannoma | Schwannoma | I | n/a | unremarkable |
| ABT SMH-134 | Schwannoma | | I | n/a | unremarkable |
| ABT SMH-136 | Schwannoma | Schwannoma | I | n/a | unremarkable |
| ABT SMH-137 | Schwannoma | | I | n/a | unremarkable |
| ABT SMH-139 | Schwannoma | Schwannoma | I | n/a | unremarkable |
| ABT SMH-141 | Schwannoma | | I | n/a | unremarkable |
| ABT SMH-142B | Schwannoma | Schwannoma | I | n/a | unremarkable |
| ABT SMH-142BR | Schwannoma | | I | n/a | unremarkable |
| ABT SMH-144 | Schwannoma | Schwannoma | I | n/a | Schwannoma, high cellularity. SOX10 + |
| ABT SMH-145B | Schwannoma | | I | n/a | unremarkable |
| ABT SMH-146B | Schwannoma | Schwannoma | I | n/a | unremarkable |
| ABT SMH-147B | Schwannoma | Schwanoma | I | n/a | unremarkable |
| ABT SMH-149B | Schwannoma | Schwanoma | I | n/a | unremarkable |
| ABT SMH-149R | Schwannoma | Schwanoma | I | n/a | unremarkable |
| ABT SMH-150B | Schwannoma | Schwanoma | I | n/a | unremarkable |
| ABT SMH-29 | Schwannoma | Schwanoma | I | n/a | unremarkable |
| ABT SMH-31 | Schwannoma | Schwanoma | I | n/a | unremarkable |
| ABT SMH-33 | Schwannoma | Schwanoma | I | n/a | unremarkable |
| ABT SMH-34A | Schwannoma | Schwanoma | I | n/a | unremarkable |
| ABT SMH-35 | Schwannoma | Cellular schwannoma | I | n/a | unremarkable |
| ABT SMH-38 | Schwannoma | Schwanoma | I | n/a | unremarkable |
| ABT SMH-39 | Schwannoma | Schwanoma | I | n/a | unremarkable |
| ABT SMH-42 | Schwannoma | Schwanoma | I | n/a | unremarkable |
| ABT SMH-43 | Schwannoma | Schwanoma | I | n/a | unremarkable |
| ABT SMH-84 | Schwannoma | Schwanoma | I | n/a | unremarkable |
| ABT SMH-85 | Schwannoma | Schwanoma | I | n/a | unremarkable |
| ABT SMH-87 | Schwannoma | Schwanoma | I | n/a | unremarkable |
| ABT SMH-89 | Schwannoma | Schwanoma | I | n/a | unremarkable |
| ABT SMH-90 | Schwannoma | Schwanoma | I | n/a | patient records used |
| ABT SMH-92 | Schwannoma | Schwanoma | I | n/a | unremarkable |
| ABT SMH-93 | Schwannoma | Schwanoma | I | n/a | unremarkable |

| Specimen ID | Expected Class | Histologic Class | Pathology Notes |
|---|---|---|---|
| *Blind Validation* | | | |
| Unknown 1 | Meningioma | Atypical meningioma | unremarkable |
| Unknown 10 | Meningioma | Meningothelial Meningioma | Attached nerve roots/ Very hemorrhagic |
| Unknown 11 | Meningioma | Atypical meningioma | unremarkable |
| Unknown 12 | Meningioma | Atypical meningioma | unremarkable |
| Unknown 14 | Meningioma | Atypical meningioma | unremarkable |
| Unknown 15 | Meningioma | Meningioma | Severe electrocuatery |
| Unknown 18 | Meningioma | Microcystic Meningioma | unremarkable |
| Unknown 19 | Meningioma | Meningioma | unremarkable |
| Unknown 20 | Meningioma | Meningioma | Fibrous with pallisading |
| Unknown 23 | Meningioma | Atypical meningioma | Mostly dura mater, minimal amount of tumor |
| Unknown 3 | Meningioma | Atypical meningioma | unremarkable |
| Unknown 4 | Meningioma | Atypical meningioma | patient records used |
| Unknown 42 | Meningioma | Meningioma | Fibrous with pallisading |
| Unknown 43 | Meningioma | Atypical meningioma | patient records used |
| Unknown 49 | Meningioma | Meningothelial Meningioma | unremarkable |
| Unknown 5 | Meningioma | Meningioma | unremarkable |
| Unknown 52 | Meningioma | Atypical meningioma | unremarkable |
| Unknown 57 | Meningioma | Meningioma | unremarkable |
| Unknown 58 | Meningioma | Meningothelial Meningioma | unremarkable |
| Unknown 60 | Meningioma | Atypical meningioma | unremarkable |
| Unknown 13 | Metastatic | n/a | unremarkable |
| Unknown 17 | Metastatic | n/a | unremarkable |
| Unknown 2 | Metastatic | n/a | unremarkable |
| Unknown 24 | Metastatic | n/a | unremarkable |
| Unknown 29 | Metastatic | n/a | unremarkable |
| Unknown 33 | Metastatic | n/a | unremarkable |
| Unknown 35 | Metastatic | n/a | unremarkable |
| Unknown 38 | Metastatic | n/a | unremarkable |
| Unknown 41 | Metastatic | n/a | unremarkable |
| Unknown 44 | Metastatic | n/a | unremarkable |
| Unknown 45 | Metastatic | n/a | unremarkable |
| Unknown 47 | Metastatic | n/a | unremarkable |
| Unknown 48 | Metastatic | n/a | unremarkable |
| Unknown 50 | Metastatic | n/a | unremarkable |
| Unknown 53 | Metastatic | n/a | unremarkable |
| Unknown 54 | Metastatic | n/a | unremarkable |
| Unknown 55 | Metastatic | n/a | unremarkable |
| Unknown 59 | Metastatic | n/a | unremarkable |
| Unknown 7 | Metastatic | n/a | patient records used |
| Unknown 9 | Metastatic | n/a | unremarkable |
| Unknown 16 | Schwannoma | Schwannoma | patient records used |
| Unknown 21 | Schwannoma | Schwannoma | unremarkable |
| Unknown 22 | Schwannoma | Schwannoma | unremarkable |
| Unknown 25 | Schwannoma | Schwannoma | unremarkable |
| Unknown 26 | Schwannoma | Schwannoma | unremarkable |
| Unknown 27 | Schwannoma | Schwannoma | unremarkable |
| Unknown 28 | Schwannoma | Schwannoma | unremarkable |
| Unknown 30 | Schwannoma | Schwannoma | unremarkable |
| Unknown 31 | Schwannoma | Schwannoma | Densely cellular. Hemosiderin |
| Unknown 32 | Schwannoma | Schwannoma | unremarkable |
| Unknown 34 | Schwannoma | Schwannoma | patient records used |
| Unknown 36 | Schwannoma | Schwannoma | unremarkable |
| Unknown 37 | Schwannoma | Schwannoma | Densely cellular. Hemosiderin |
| Unknown 39 | Schwannoma | Schwannoma | unremarkable |
| Unknown 40 | Schwannoma | Schwannoma | unremarkable |
| Unknown 46 | Schwannoma | Schwannoma | unremarkable |
| Unknown 51 | Schwannoma | Schwannoma | unremarkable |
| Unknown 56 | Schwannoma | Schwannoma | unremarkable |
| Unknown 6 | Schwannoma | Schwannoma | unremarkable |
| Unknown 8 | Schwannoma | Schwannoma | Severe electrocuatery |

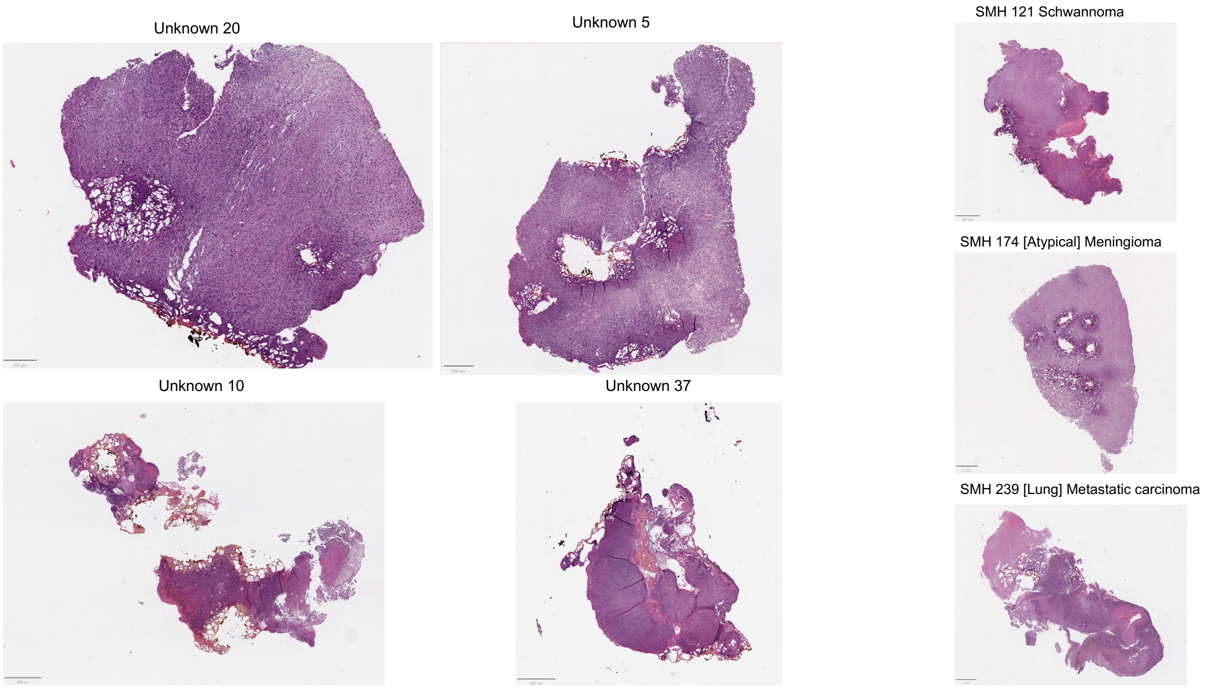


**Figure S3. Post PIRL-MS hematoxylin and eosin (H&E) images of select unknowns with misclassifications as reported in Table S4.** Here we show the digitized H&E images of unknowns 5, 20, 10 and 37. These specimens contained a variety of heterogeneity such as hemorrhages, nerve roots, hemosiderin and dense cellularity as well as fibrous with palisading types (see Table above). For comparison, we have included representative H&E images of meningioma (SMH 174, atypical), schwannoma (SMH 121) and metastatic carcinoma (SMH 239, lung). The laser sampling spots are particularly visible in this level section of SMH 174, enabling the tracking of sampling event data to locations in the tumour.

*Rationalization of the method failures*

The distinctions between schwannoma, meningioma and metastatic cancers (as control) discussed in the main text, however, were not flawless and misclassifications between schwannoma and meningiomas existed (See Table S4). Schwannoma unknowns 21, 26, 28 and 51 had only a few (1-3) of their sampling events misclassified, but each had more correctly classified events than misclassified. However, all of the 7 schwannoma sampling events belonging to unknown 37 were misclassified as meningioma (Table S4). A slightly different pattern existed for meningiomas with unknowns 5,10 and 20 possessing most of the misclassifications with unknown 23 contributing only slightly (1 misclassified event out of 9 total for this specimen). All of the misclassified metastatic tumour data came from unknown 38 with 3 of its total 9 sampling events misclassifying as schwannoma. No metastatic cancer was misclassified as meningioma.

Unknown 20 with significant misclassifications as schwannoma (Table S4) was a fibrous meningioma (See SI, Specimens and Histology section) which has the highest propensity to mimic schwannoma on a frozen section assessment [5,6]. However, unknown 42 which was also a fibrous meningioma was classified correctly based on its PIRL-MS profile (Table S4). To further investigate the misclassifications, we resorted to more detailed histological analyses and evaluation by a neuropathologist using both hematoxylin and eosin (H&E) as well as immunohistochemistry (IHC) utilizing antibody reactivity against Sox10 which is a suitable method for definitive diagnosis of meningiomas[7]. In this assay, meningiomas are deemed negative for Sox10 reactivity and schwannomas show a strong positive signal. Here, post PIRL-MS assessment of H&E slides for all of the misclassified data, however, failed to recover any aberrant heterogeneity not present in the model to rationalize the method failure (see the above section Specimens and Histology).The misclassified unknowns 5, 10,20 and 37 possessed Sox10 staining levels (positive for schwannoma and negative for meningioma) consistent with final pathology diagnoses from H&E (See Table S4). Therefore, no discordant gross pathology annotations existed for these specimens to rationalize their misclassifications by PIRL-MS. However, unknown 10 possessed attached nerve roots and was hemorrhagic (See SI, Specimens and Histology section). The unknown 37 on the other hand was densely cellular and hemosiderin. Having said this, however, the unknown 31 was also densely cellular and hemosiderin but did not show significant misclassifications (Table S4).

With histopathology not providing a clearcut rationale for the misclassifications seen, we resorted to a rigours assessment of the quality of the mass spectral data obtained for the misclassified unknowns and comparing those to correctly classified specimens. Here, a notable difference correlated with potential misclassified cases emerged based on specimen's signal intensity which was used for data normalization. This is merely a hypothesis that must be validated through the analysis of additional specimens, modeling low TIC (intensity) data and/or use of alternative data normalization strategies to lessen the impact of overall signal intensity variations during data normalization. Here, the correctly classified meningiomas and schwannomas possessed average (± standard deviation) TIC values of $(4\pm2)\cdot10^{6}$ and $(5\pm2)\cdot10^{6}$ respectively. The misclassified meningiomas and schwannomas had TIC values of $(5\pm2)\cdot10^{6}$ and $(3\pm1)\cdot10^{6}$, respectively. As most meningioma and schwannomas were misclassified with one another, the reversal of TIC trend between correctly classified and incorrectly classified intradural extramedullary tumours cancers is nevertheless notable. In a similar vein, through inspecting the coefficient of correlation (spectral similarity between unknown and model specimens), it is noted that the misclassified unknown 20 possesses PIRL-MS spectra that are

distinct from its true class (actual $4.02 \cdot 10^5$ compared to the expected value of $(3.20 \pm 0.60) \cdot 10^5$). Similarly, out of range (for expected true class) coefficient of correlation was seen for unknown 5 ($2.48 \cdot 10^5$ versus $(3.20 \pm 0.60) \cdot 10^5$). Surprisingly, no notable difference was seen for unknown 10 meningioma with the observed coefficient of correlation of $3.25 \cdot 10^5$ being in range for the expected true meningioma class possessing the value of $(3.20 \pm 0.60) \cdot 10^5$. Likewise, the coefficient of correlation coefficient for unknown 37 schwannoma of $3.01 \cdot 10^5$ was in range for expected value of $(3.05 \pm 0.40) \cdot 10^5$ for schwannoma tumours. These observations did not provide a rationale for why the unknowns 10 and 37 misclassified. However, they provided an orthogonal assessment for why unknowns 20 and 5 contained markedly different chemical information in their mass spectra, potentially leading to their misclassifications using PCA-LDA comparisons after dimensionality reduction. It is important to note that even the correctly classified meningioma unknowns, however, possessed 5 out-of-range specimens (unknowns 3,14,52,58,60) compared to schwannomas with only 2 (unknowns 25 and 30). Here, only one metastatic tumour specimen (unknown 7) was correctly classified despite possessing out of range coefficient of correlation value ($5.44 \cdot 10^5$ compared to $(3.05 \pm 0.90) \cdot 10^5$), albeit using only one sampling event. The heterogeneity of meningioma signal may be in line with the histologic heterogeneity of meningioma tumours used in this study across grades 1 and 2 as well as histological types of fibrous, meningothelial, atypical or microcystic in the unknown cohort among additional classes of psammomatous and transitional present in the PCA-LDA model (See SI, Specimens and Histology section). Here, the metastatic cohort possessed the largest standard deviation amongst the group, reflecting on its heterogeneity arising from different organs of origin. Taking these results into consideration, the coefficient of correlation analysis may not have sufficient 'sensitivity' to unequivocally rationalize the misclassification of meningiomas (and within the standard deviation appears only most rigorous in assessing metastatic tumours that are expected to be much more distinct than either of the schwannoma and meningioma tumours anyway).

While the coefficient of spectral similarity results above suggested spectral dissimilarity (compared to expected classes) for unknowns 20 and 5, they failed to rationalize why unknowns 10 and 37 showed such strong misclassifications. To further investigate this, we performed 'qualitative' mass spectral covariance analysis for these two specimens as well as for unknowns 5 and 20, qualitatively comparing the patterns of their covariance maps to those of their respective classes comprised of correctly classified unknowns listed in Table S4. Consistent with the coefficient of similarity results (albeit against specimens of the model), however, unknown 20 possessed drastically different covariance map compared to its expected pathology class (Fig. S4). Unknown 5, on the other hand possessed both meningioma and schwannoma like patterns in its covariance map (Fig. S4) and as such demonstrated classifications as both these tumour types across its multiple sampling events. For this specimen, utilizing the sparse analysis improved the classification accuracy from 36.4% to 91.0 % (Table S4) suggesting that meningioma-specific *m/z* values existed in its spectra, utilized by the sparse model potentially leading to this improvement. Hemorrhagic unknown 10 with the attached nerve root exhibited a covariance map that did not resemble either of its misclassified classes, and as such its classification could not be improved by use of sparse modeling as it did for unknown 5 (Table S4). Unknown 37, possessed a covariance map that most closely resembled that of meningioma (Fig. S4) which could explain the misclassifications seen. Having said all these, an open question still remains; why the unknowns 5,10,20 and 37 possessed such aberrant PIRL-MS signatures in the absence of any gross pathologic abnormality? At present we are unable to answer this question. However, we have demonstrated that if aberrant (e.g., based on covariance maps) mass spectral signals exist, then

misclassifications could take place. Additional work is required to investigate the origin of this aberrant signal, aside from the potential contributions of local heterogeneity (e.g., presence of nerve roots, hemorrhaging areas among others). Our observations, nevertheless, may further supports the need for a data-dependent spectral quality assessment implemented as a 'filtering' step prior to commitment to classification. This way, the data from specimens with drastically different (or unexpected) mass spectra are not progressed to classification against the model with inexplicable prediction results (and thus unintended clinical consequences). Further mining of mass spectral data is required to decipher a ruleset for such an effort, if at all feasible. At the very least, the feature-based classification as demonstrated for the unknown 5 may constitute a veritable approach to obtaining a rigorous classification for mass spectra that are 'contaminated' with undesirable signals. In a similar vein, we also investigated the 4 specimens that constituted 11 misclassified sampling events in the cross-validation of the Fig. 2A PCA-LDA model (Table S2). While high cellularity and necrosis was noted in two of these four specimens (ABT SMH-144 and ABT SMH-158), the pathological assessment of the other two (ABT SMH-166 and ABT SMH-191) was unremarkable. Here, however, Table S1 results suggest that ABT SMH-158 and ABT SMH-166 specimens possessed signal intensities on the lower range of acceptability for their class and ABT-SMH-191's signal intensity was typical of those seen for schwannomas. These observations may further reenforce the need for mass spectral quality assessment before classification as speculated above.

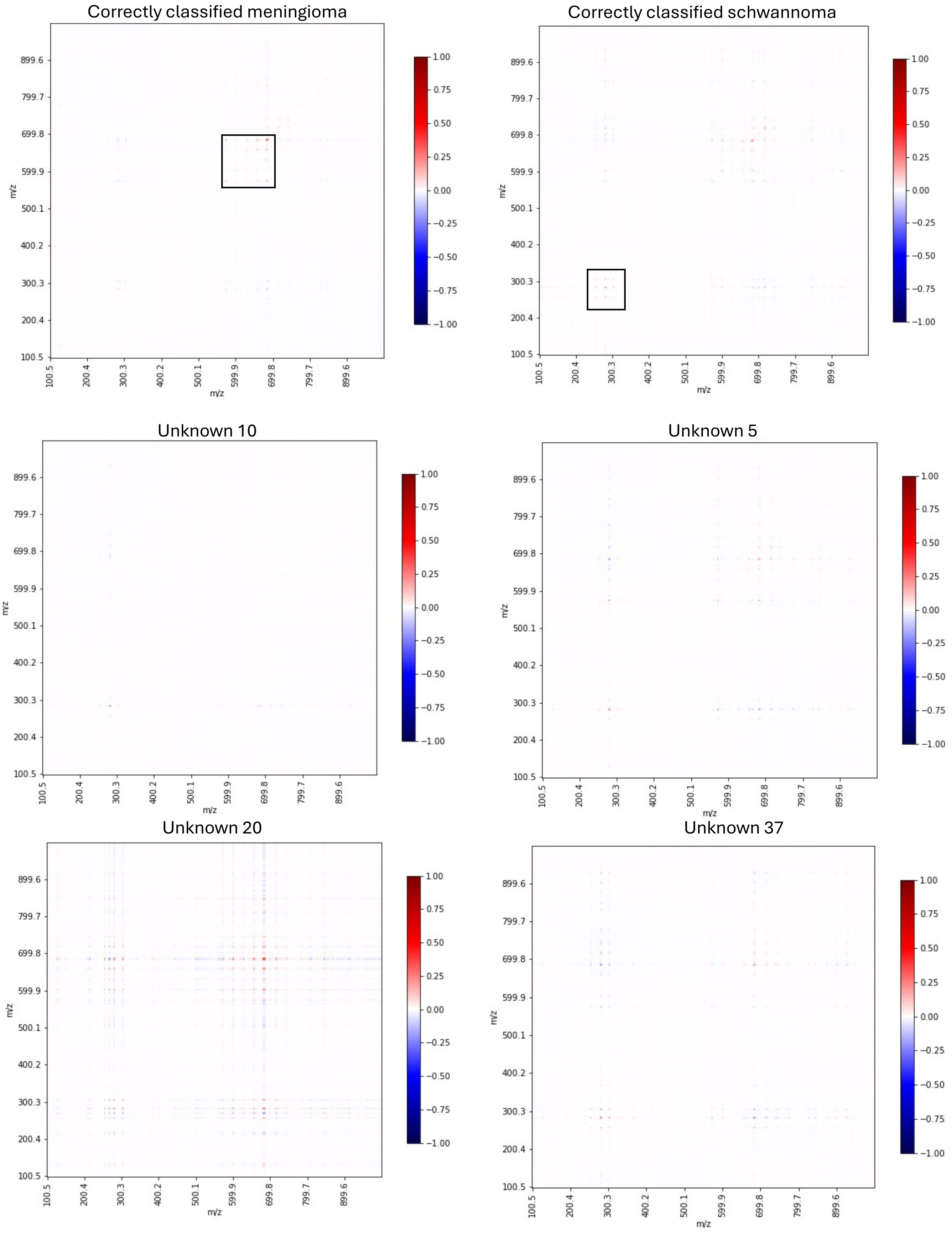
Correctly classified meningioma
Correctly classified schwannoma
Unknown 10
Unknown 5
Unknown 20
Unknown 37
m/z
m/z
100.5
200.4
300.3
400.2
500.1
599.9
699.8
799.7
899.6
1.00
0.75
0.50
0.25
0.00
−0.25
−0.50
−0.75
−1.00

**Figure S4. Qualitative covariance map analysis of select unknowns with misclassifications as reported in Table S4.** Covariance maps for unknowns 5,10,10 and 37 were created as described in the text and compared to those of correctly classified meningioma and schwannoma unknowns. Typical fingerprint areas characteristic for correctly classified meningioma and schwannomas are highlighted by two squares in the first (control) panels.

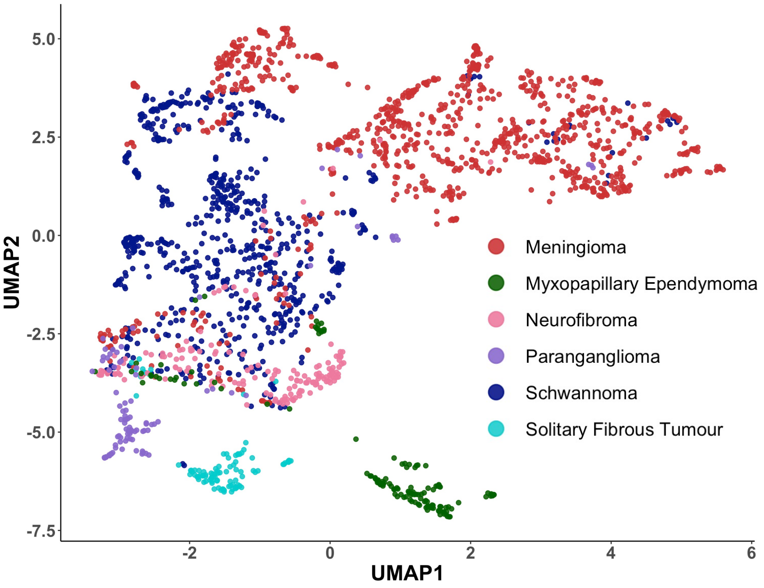


**Figure S5. Unsupervised analysis of PIRL-MS signatures of intradural extramedullary spinal tumours in differential diagnosis.** In this figure we show an unsupervised clustering of PIRL-MS data using dimensionality reduction with Uniform Manifold Approximation and Projection (UMAP) generated from n=257 independent tumours listed in the legend in Fig 3 (using n=2,148 mass spectra). These tumours are in differential diagnosis with one another[8,9].

**Additional information: Experimental methods**

*Specimens and pathology*

The specimens obtained from the bank were transported on dry ice (compliant with provisions of the Transport of Dangerous Goods act), stored at -80° C prior to use, thawed at room temperature and subjected to PIRL-MS analysis inside a biological safety cabinet. True (ground truth) pathology annotations were created by a neuropathologist evaluating a hematoxylin and eosin (H&E) stained slide of each specimen post PIRL-MS sampling, as well as paraffin embedded sections and immunohistochemistry from other blocks of the same specimen where appropriate. Prior to H&E staining, the specimens were formalin fixed and paraffin embedded. In few cases where such slides were of poor diagnostic quality or cases in which dispensed tissue pieces were small (< $2mm^2$) and thus entirely used up during multiple PIRL-MS sampling (each utilizing ~ $0.5mm^3$ of tissue) preventing post-sampling pathology verification, patient records were consulted for permanent pathology results, taken as ground truth. The ground truth pathology assessment for additional specimens in Fig. 3 were based on a combination of post PIRL-MS assessment by neuropathologist and patient records. In 2 of the additional meningiomas used in this figure, significant connective tissue and in one instant pieces of bone were present. Immunohistochemistry (IHC) for Sox10 was performed on fixed tissue slices using standard protocols [7], upon deparaffinization with EZ prep solution (Ventana) utilizing Sox 10 clone SP267 (Primary Ab) and Universal (secondary Ab) with HRP for detection. All slides were digitized using an Aperio AT2 Brightfield scanner at 20X with images presented without further processing.

*PIRL-MS data analysis*

We divided each mass spectrum into 9,000 bins of 0.1 Da width. Therefore, each mass spectrum forms a data point comprised of 9,000 *m/z* features subjected to multivariate statistical analysis. The multivariate modeling, cross-validation and classification of blind samples used principal component analysis, linear discriminant analysis (PCA-LDA) on Abstract Model Builder (AMX) [3] (version 1.0.1360.0, Waters Research Centre, Budapest, Hungary)[3] previously utilized in PIRL-MS research[4,10]. The 'full group' leave-out function ensures that the rest of dependent sampling events from same specimen are excluded from test and validation sets. However, in our approach we used only 5-fold iterations of the full group leave-out (for a 20% leave-out scheme) and did not further iteratively perform a *k*-fold or *v*-fold type cross-validation test[2,11]. Therefore, our observations may be limited. However, all sensitivity and specificity calculations used an independent specimen test set (in a blind manner) and considering this we are not concerned regarding the limited scope of cross-validation tests performed herein. We utilized the following AMX parameters[3]: (1) number of scans per spectrum was set to 13, (2) wait for good spectrum timeout was also 13 seconds, (3) the total ion count (TIC) to commence counting scans was $>10^4$. The Mahalanobis[1] probability thresholds from AMX[3] used the 'Standard Deviation' mode with a value of 4 for modeling, and for recognition in a blind manner (i.e., classification of test specimens of established histopathology annotations to which the MS operator and the data analyst were both blinded to). PIRL-MS data points with less than or equal to 3 seconds of signal duration were excluded from the analysis as done previously [4]. Sparse analysis used the 'low-complexity' peak list mode on AMX[3], and all spectra were lock-mass corrected using the internal *m/z* 717.5070 present in all the specimens. The calculations of sensitivity and specificity used standard definitions of true positive and true negative rates, respectively, and the probability in prediction

values from Mahalanobis distance mapping[1] was reported as output by AMX. The cross-validation statistics are reported in two formats of 'including' and 'excluding' outlier (unclassifiable) data points. As no clinical decision will be made based on unclassifiable data, we have excluded them from our calculations of the sensitivity and specificity. Here, it must be emphasized that multiple sampling events from the same specimen is encouraged during deployment to address unclassifiable data, should they occur. While n=24 sampling events out of the n=487 attempts resulted in data of unacceptable quality (defined as signal duration of less than or equal to 3 seconds), only n=16 sampling attempts out of n=487 resulted in unclassifiable data. Therefore, the duty cycle of the method in rendering classification is high. The sensitivity and specificity values as well as cross-validation statistics reported in the text and tables are raw values obtained from calculations and included 'as is' for transparency. The average values are reported with ±1 standard deviation and have used scientific and decimal reporting conventions for ease of interpretation and comparison.

Mass spectral graphics were created with MassLynx (Waters, Milford, MA, USA). UMAP plots were generated with R utilizing the package "UMAP" with Euclidian distance measurements. The visualization utilized "ggplot2". The number of components was set to 2, and the number of "epochs" 200. The number of negative sample rate was 5. The minimum distance (min_dist) was 0.1, and number of neighbours being set to 25 to provide reasonable local structure differentiation between the groups based on world health organization (WHO) handbook of differential diagnosis[9]. We additionally, used the random seed of 123 for our plots, and the number of iterations (n_iterations) was set to 250.

Mass spectral similarity was assessed from the Pearson correlation coefficient of spectral features between a specimen and its respective baseline class. To avoid a significant number of "bins" carrying a zero variance we averaged the PIRL-MS spectra over a 1 Da range. Mass spectral similarly was then calculated as the sum of the absolute value of the difference between coefficient of correlation for each feature in a 900 by 900 matrix comprised of samplings events from each unknown compared to its respective baseline class matrix from the model used to classify the unknowns. The Pearson correlation coefficient was calculated in Python using corrcoef() from the Numpy package. Covariance analysis was performed in a similar manner as described above, with the covariance between each binned *m/z* value being calculated from all sampling events for a given sample or all correctly classified samples for a given class, generating a 900 x 900 matrix. This analysis was completed using the cov() function in the pandas package and visualized in a heatmap using the plotly package.

*Identification of tumour type classifying ions*

The loading plots generated across all linear discriminant (LD) dimensions (on AMX[3]) using PCA-LDA informed the top 100 most important ions for the multivariate discriminations across meningioma, schwannoma, and metastatic tumours (25 positive influencing and 25 negative influencing across each LD for 2 LDs in total). Binned PIRL-MS spectral data with less than 50 mDa shift between Xevo time-of-flight (resolving power ~15,000-20,000 full width at half maximum (FWHM), for PIRL-MS) and Synapt time-of-flight (resolving power ~100,000 FWHM, for UPLC) were considered a match to identify $MS^1$ peaks on Synapt spectra for identification of retention time (RT) and fragment patterns as detailed below. UPLC-MS/MS analysis of lipid extracts (one representative specimen for each of the 3 tumour types examined) utilized an

ACQUITY UPLC (Waters Milford, MA, USA) coupled to a Synapt qTOF (Waters, Milford, MA, USA). Electrospray ionization (ESI) in the negative ion mode was used to` collect the lipid profiles.

As laser-mediated desorption of tissue lipids (trapped laser plume on filter paper) and solvent extraction directly from tissue were shown largely compatible [29,12], we prepared (and subsequently dried with $N_2$) lipid extracts from ~100 $mm^3$ of tumour tissue (in 150 µL of water with 190 µL of methanol and 370 µL of chloroform, subjected to vortexing for 2 min and centrifugation at 13,000 rpm for 5 min, and separation of apolar phase followed with additional centrifugation). Reconstitution used a 4:3:1 (volumetric) isopropanol:acetonitrile:water. Subsequent vortexing for 2 min and ultrasonication for 10 min (repeated twice), the content was subjected to analysis in the negative ion mode over *m/z* 100-1,000. The 'lock-mass' mode used Leucine-Enkephalin (injection volume of ~2 µL at 10 °C). The ESI capillary and cone voltages were ~2.0 kV and 30 V, respectively with source and desolvation temperatures of 150 °C and 500 °C. The cone and desolvation gas flow rates were 150 and 600 L/h. Spectra were collected using MassLynx 4.2 (Waters). The scan time in the ms/ms mode was 300 ms, and the collision energy ramp was between 30-55 V. A BEH C18 column (2.1 X 100mm, 1.7µm, Waters) was used (at 55 °C). A gradient (0.4 mL/min) between mobile phase A (water:acetonitrile 2:3, 10 mM ammonium formate, 0.1% formic acid) and mobile phase B (isopropanol:acetonitrile 9:1, 10 mM ammonium formate, 0.1% formic acid) was established. The parameters were: 100-50 % mobile phase A (5 min), 50-30% (12 min), 30-0% (0.1 min) with a hold at 100% B (0.4 min). LipidMaps online analysis tool[13] was used wherein we considered various ion adduct forms as well as tolerances of ±0.005 or ±0.01 Da (with respect to observed accurate mass values and isotopic patterns of the parent ions) in the process of identifying positive hits. Additional application of $MS^2$ pattern was used to more accurately identify lipids or lipid classes of interest. The positive hits with results < 5ppm (expected compound mass versus UPLC-MS accurate mass) were reported. Additionally, where the database search above suggested multiple hits satisfying the criteria used, we solely reported the hits with the smallest mass shift compared to the expected PIRL-MS *m/z* value for that hit.

## References


1. Yao L, Lin TB. Evolutionary Mahalanobis Distance-Based Oversampling for Multi-Class Imbalanced Data Classification. *Sensors (Basel).* 2021; 21(19).
2. Fiorante A, Ye LA, Tata A, et al. A Workflow for Meaningful Interpretation of Classification Results from Handheld Ambient Mass Spectrometry Analysis Probes. *International Journal of Molecular Sciences.* 2024; 25(6):3491.
3. Bodai Z, Cameron S, Bolt F, et al. Effect of Electrode Geometry on the Classification Performance of Rapid Evaporative Ionization Mass Spectrometric (REIMS) Bacterial Identification. *J Am Soc Mass Spectrom.* 2018; 29(1):26-33.
4. Woolman M, Kuzan-Fischer CM, Ferry I, et al. Picosecond Infrared Laser Desorption Mass Spectrometry Identifies Medulloblastoma Subgroups on Intrasurgical Timescales. *Cancer Res.* 2019; 79(9):2426-2434.
5. Chand P, Amit S, Gupta R, Agarwal A. Errors, limitations, and pitfalls in the diagnosis of central and peripheral nervous system lesions in intraoperative cytology and frozen sections. *J Cytol.* 2016; 33(2):93-97.
6. Rao S, Rajkumar A, Ehtesham MD, Duvuru P. Challenges in neurosurgical intraoperative consultation. *Neurol India.* 2009; 57(4):464-468.
7. Ng J, Celebre A, Munoz DG, Keith JL, Karamchandani JR. Sox10 is superior to S100 in the diagnosis of meningioma. *Appl Immunohistochem Mol Morphol.* 2015; 23(3):215-219.
8. Figarella-Branger D, Appay R, Metais A, et al. [The 2021 WHO classification of tumours of the central nervous system]. *Ann Pathol.* 2022; 42(5):367-382.
9. Louis DN, Perry A, Wesseling P, et al. The 2021 WHO Classification of Tumors of the Central Nervous System: a summary. *Neuro Oncol.* 2021; 23(8):1231-1251.
10. Katz LW, Michael; Kiyota,Taira ; Pires, Layla; Zaidi, Mark; Hofer, Stefan O.P.; Leong, Wey; Wouters, Brad G.; Ghazarian,Danny; Chan, An-Wen; Ginsberg, Howard J.; Aman, Ahmed; Wilson, Brian C.; Berman, Hal K.; Zarrine-Afsar, Arash. Picosecond infrared laser mass spectrometry identifies a metabolite array for 10-second diagnosis of select skin cancer types: A translational proof of concept study. *Anal Chem* 2022; Accepted.
11. Arlot S, Celisse A. A survey of cross-validation procedures for model selection. *Statistics Surveys.* 2010; 4(none):40-79, 40.
12. Woolman M, Kiyota T, Belgadi SA, et al. Lipidomic-Based Approach to 10 s Classification of Major Pediatric Brain Cancer Types with Picosecond Infrared Laser Mass Spectrometry. *Anal Chem.* 2024; 96(3):1019-1028.
13. Fahy E, Sud M, Cotter D, Subramaniam S. LIPID MAPS online tools for lipid research. *Nucleic Acids Res.* 2007; 35(Web Server issue):W606-612.
